# Mutation Spectra of the MRN (MRE11, RAD50, NBS1/NBN) Break Sensor in Cancer Cells

**DOI:** 10.3390/cancers12123794

**Published:** 2020-12-16

**Authors:** Matthew T. McPherson, Ashton S. Holub, Aman Y. Husbands, Ruben C. Petreaca

**Affiliations:** Department of Molecular Genetics, The Ohio State University, Columbus, OH 43215, USA; mcpherson.166@buckeyemail.osu.edu (M.T.M.); holub.28@buckeyemail.osu.edu (A.S.H.); husbands.6@osu.edu (A.Y.H.)

**Keywords:** DNA double strand break (DSB), COSMIC, break sensor, MRN, genetic recombination

## Abstract

**Simple Summary:**

A DNA double strand break cuts a chromosome in two and is one of the most dangerous forms of DNA damage. Improper repair can lead to various chromosomal re-arrangements that have been detected in almost all cancer cells. A complex of three proteins (MRE11, RAD50, NBS1 or NBN) detects chromosome breaks and orchestrates repair processes. Mutations in these “break sensor” genes have been described in a multitude of cancers. Here, we provide a comprehensive analysis of reported mutations from data deposited on the Catalogue of Somatic Mutations in Cancer (COSMIC) archive. We also undertake an evolutionary analysis of these genes with the aim to understand whether these mutations preferentially accumulate in conserved residues. Interestingly, we find that mutations are overrepresented in evolutionarily conserved residues of RAD50 and NBS1/NBN but not MRE11.

**Abstract:**

The MRN complex (MRE11, RAD50, NBS1/NBN) is a DNA double strand break sensor in eukaryotes. The complex directly participates in, or coordinates, several activities at the break such as DNA resection, activation of the DNA damage checkpoint, chromatin remodeling and recruitment of the repair machinery. Mutations in components of the MRN complex have been described in cancer cells for several decades. Using the Catalogue of Somatic Mutations in Cancer (COSMIC) database, we characterized all the reported MRN mutations. This analysis revealed several hotspot frameshift mutations in all three genes that introduce premature stop codons and truncate large regions of the C-termini. We also found through evolutionary analyses that COSMIC mutations are enriched in conserved residues of NBS1/NBN and RAD50 but not in MRE11. Given that all three genes are important to carcinogenesis, we propose these differential enrichment patterns may reflect a more severe pleiotropic role for MRE11.

## 1. Introduction

Cancers are characterized by structural chromosomal instability such as translocations, deletions, inversions, duplications, and other forms of copy number variations. These aberrations which may affect the function of cell cycle regulators arise from inappropriately repaired mitotic DNA double strand breaks (DSBs) [1]. Several pathways of DSB repair have evolved in eukaryotic cells including homologous recombination (HR) and non-homologous end-joining (NHEJ) [2,3]. HR is cell cycle regulated with functions mainly during S-phase and G2. In fact, recombination evolved in bacteria precisely to deal with damage arising from replicating long genomes [4]. The process is essential for genome integrity and has been conserved in eukaryotes. It was subsequently also adapted for meiosis.

**Figure 1:**
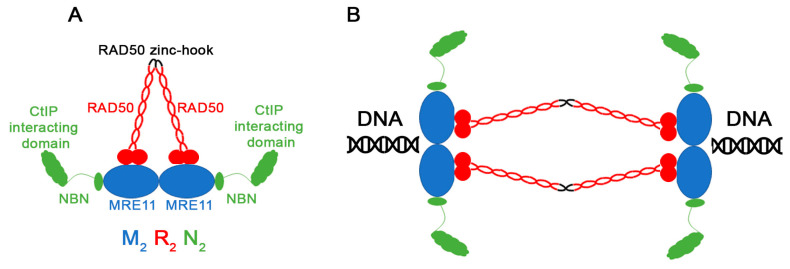
MRE11, RAD50 and NBS1/NBN form a hexameric complex. (**A**) Two subunits each of MRE11, RAD50, and NBN assemble in a hexameric complex to form a ring. The coil-coiled domains of RAD50 (red rope like diagrams) facilitates this ring structure. (**B**) A different structure assumed by the MRN complex to tether the broken chromosome ends together. Adapted from Lafrance-Vanasse et al., 2015.

The MRN complex (MRE11, RAD50, NBS1) is a break sensor and adaptor in eukaryotes [5,6,7]. MRE11 and RAD50 are conserved from bacteria to humans [8] while NBS1 is only found in Eukaryota [9,10]. NBS1 is also known as NBN in humans and XRS2 in *S. cerevisiae* The yeast XRS2 gene has some significant differences from NBS1 [11,12]. MRE11 and RAD50 were initially shown in *S. cerevisiae* to facilitate meiotic recombination [13,14]. MRE11 was later shown to be have both endo- and exonuclease activities involved in localized resection of double stranded DNA ends to prepare single stranded DNA substrates for repair [15,16]. Crystal structures of MRE11, RAD50 and NBN/NBS1 in both humans and more-basal eukaryotes revealed that the three proteins form a hexameric complex containing two subunits of each MRE11, RAD50 and NBS1 [17,18,19,20,21,22,23] (Figure 1).

The functional complexity of MRN in recognizing the double strand break has been actively studied for the last few decades [24]. Structure and sequence conservation analysis has revealed that the complex forms a ring similar to the Structural Maintenance of Chromosome (SMC) proteins [25,26,27,28] (Figure 1A). RAD50 has two coil-coiled domains that allow it to fold on itself so that both the N- and C-termini interact with MRE11 while the middle part of the protein forms a dimer through a zinc-hook domain [29,30]. Two NBS1/NBN monomers interact with the MRE11 subunits. The NBS1/NBN N-terminus interacts with CtIP/RBBP8, an adaptor that facilitates MRN function [31,32,33,34,35]. Interaction of RAD50 with ATP (not shown in the figure) induces a conformational change in the ring which facilitates the enzymatic activity of the MRN complex [18,20,36,37,38,39,40].

The MRN complex recognizes both exogeneous DSBs as well as damage that might arise in S-phase during DNA replication [3,41,42]. Homologous recombination requires long range resection to expose tracts of single stranded DNA which participate in homology search and strand invasion [43]. The MRN complex initiates localized resection but Exo1 as well as several other factors discussed elsewhere facilitate long-range resection [24,44,45]. Competition between the MRN complex and the other “break sensor”, the NHEJ Ku70/80 heterodimer, can bias repair towards NHEJ and other related pathways [24,46,47,48,49,50]. The MRN complex also function to tether the two broken ends together which is mediated by the flexible coil-coiled regions of RAD50 [51,52,53] (Figure 1B). In addition, to its resection activities, the MRN complex mediates communication among DNA damage checkpoint factors, chromatin remodeling factors, and DNA damage repair machinery [26,54,55]. Thus, the MRN complex is central to DSB repair.

Mutation of any MRN subunit can severely compromise DSB repair [56]. Phenotypes may range from inappropriate repair producing undesirable structural chromosomal instability to no repair, which may kill the cell. These undesirable chromosome products are hallmarks of cancer cells [57]. Several diseases directly linked to MRN mutations such as the *Nijmegen Breakage Syndrome* have been identified and are reviewed elsewhere [56,58,59]. Additionally, many cancer cells are characterized by MRN mutations ranging from amino acid substitution to complete deletion of the gene [56].

The Catalogue of Somatic Mutations in Cancer (COSMIC) database is a repository of genetic mutations in cancers from various sources including primary tumors and cell lines [60]. Some COSMIC data have been previously reported in the literature while others are novel findings. The goal of this study was to query this database and map all MRE11, RAD50 and NBS1/NBN mutations appearing in cancer cells. This analysis presents a mutation spectrum map of MRN and highlights several mutational hotspots predicted to abolish interactions within the complex. In addition to identifying mutational hotspots in MRN, we find that, unlike RAD50 and NBS1/NBN, mutations in MRE11 do not primarily fall in evolutionarily conserved residues. This suggests that mutations in conserved residues of MRE11 are less tolerated than those of RAD50 or NBS1/NBN, possibly because these mutations are not advantageous to cellular transformation or cancer progression. Finally, we identify a mutational hotspot in the MRN-interacting partner CtIP/RBBP8. Interestingly, this hotspot is also predicted to lead to truncations that abolish interaction with members of the MRN complex.

## 2. Materials and Methods

### 2.1. COSMIC Data

**Figure 2:**
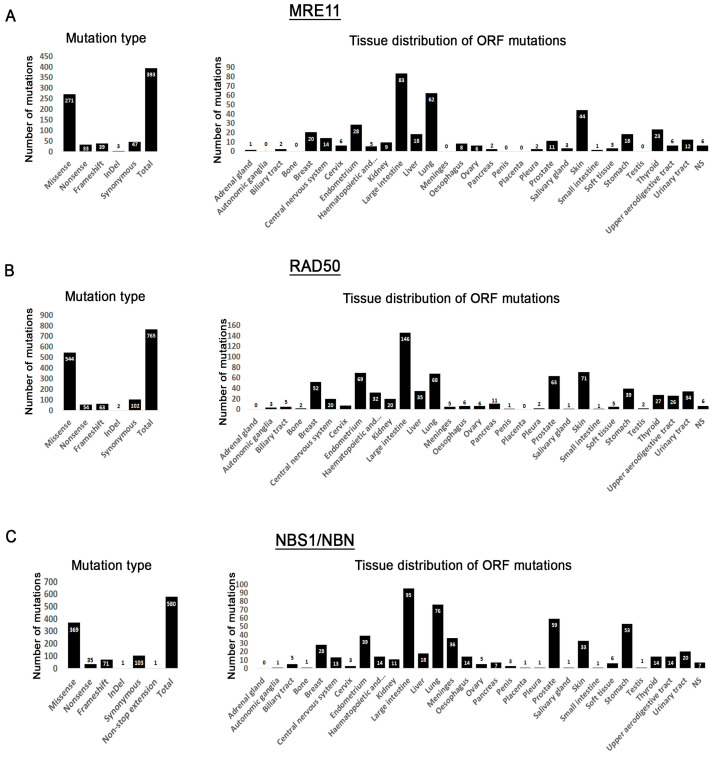
COSMIC mutation distribution for MRE11, RAD50 and NBS1/NBN. (**A**–**C**) Graphs to the left show the mutation distribution by type. Intronic and 5′ and 3′ UTR mutations have been removed but are reported in Supplementary Table S1. The right graphs show distribution of ORF mutations by tissue. For some tissues, no mutations are shown, and it is indicated in these graphs with a “0”.

Three Excel files with MRN mutations and one with CtIP/RBBP8 mutations were downloaded from https://cancer.sanger.ac.uk/cosmic (version 91, hg38). This is a publicly available repository for cancer mutations and the data is freely available. COSMIC deposits both primary patient data and cell line data from various sources including the NIH The Cancer Genome Atlas (TCGA), The Cell Lines Project, as well as other independent studies [60]. For most samples, COSMIC lists PubMed IDs which we used to compile Table S1. COSMIC provides Sample IDs for each mutation and therefore it is possible to determine how many times the same mutation has been identified in different patients or cell lines. The tissue from which the mutation is identified (tumor type) is also indicated. These data are represented in Figure 2 and Figure 8. To ensure that we did not overcount mutations, we visually inspected the data and deleted duplicates that may have been reported independently by two different studies.

SPSS (version 25) under Ohio State University (OSU) license was used for most graphs. Some graphs were made in Excel. Mutation lollipops were made using previously described software [61]. All diagrams and figures were made in Photoshop.

### 2.2. Evolutionary Analyses

Using NCBI’s Blastp program and the reference protein database, 27, 25, and 25 full-length sequences of MRE11, RAD50 and NBS1/NBN, respectively, were extracted, spanning >1 billion years of evolution [60]. Sequences were aligned using MAFFT version 7 with the default settings [62,63]. Conserved residues were analyzed using R and the “msa” package from Bioconductor using a stringent 98% consensus threshold [64]. This threshold accounts for rare mutations that may represent most sequences [65]. Residues were shaded according to similarity and a consensus logo added using R and Bioconductor. The file was then exported in .tex, compiled using TeXworks and saved as a pdf (Figure S1).

## 3. Results and Discussion

### 3.1. MRN Mutation Spectrum in Cancer Cells

To investigate the mutation spectrum of the MRN complex in cancer cells, we queried the COSMIC database. All three genes (MRE11, RAD50 and NBS1/NBN) are characterized by a larger number of missense mutations while non-sense, frameshift, InDel, and synonymous mutations comprise a smaller fraction (Figure 2A–C, Table S1). We excluded intronic and 5′ and 3′ UTR mutations from further characterization as they are not likely to change the protein function. Conversely, synonymous mutations were included as some recent reports suggest that they may affect the mRNA stability [66]. Generally, MRN non-intronic mutations are found in all tissues queried but the large intestine is more represented than all other tissues. It is not clear if cancers of the large intestine are more likely to have MRN mutations or if more studies from these cancers have been reported. The liver, lung, breast, prostrate, skin and endometrium are several other tissues with a higher incidence of mutations.

### 3.2. Mutation Distribution in MRE11, RAD50 and NBS1/NBN

**Figure 3:**
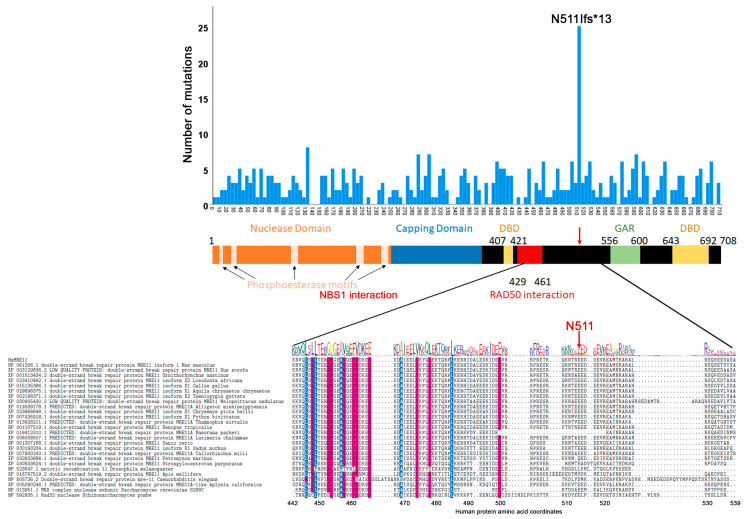
Distribution of MRE11 ORF mutations by amino acid position. The distribution graph is overlayed on a cartoon of MRE11. The N511Ifs*13 mutation falls between the RAD50 interacting domain and the GAR domain. The multiple sequence alignment shows that this mutation is not in a conserved region of MRE11.

MRE11 and RAD50 are conserved from bacteria to humans [28]. An alignment of several MRE11 and RAD50 eukaryotic species sequences further highlights the high level of conservation within key functional domains (Supplemental Figure S1). Crystal structures of MRE11 from several species including humans show that the N-terminal domain contains a nuclease domain which constitutes the catalytic site and a capping domain that modulates the active site interaction with the substrate [17,18,19,20,21,22,23] (Figure 3). A DNA binding domain and the region that interacts with RAD50 are found in the central part of the protein [17,53,67]. The C-terminus of the protein is characterized by a glycine-arginine-rich (GAR) domain. At least one study showed that in humans methylation of several arginine residues within this domain by the PRMT1 methylase facilitates MRE11 localization to DNA double strand breaks [68]. Finally, MRE11 is characterized by a second DNA binding domain in its C-terminal region. Graphing the amino acid positions of all MRE11 ORF mutations reported on COSMIC revealed a hotspot (Figure 3). The N511fs*13 frameshift mutation [69,70,71,72,73,74,75,76] introduces a stop codon deleting the C-terminal region of the protein that includes the GAR and the second DBD domain. This mutation is therefore predicted to severely affect MRE11 interaction with DNA double strand breaks but not with RAD50.

**Figure 4:**
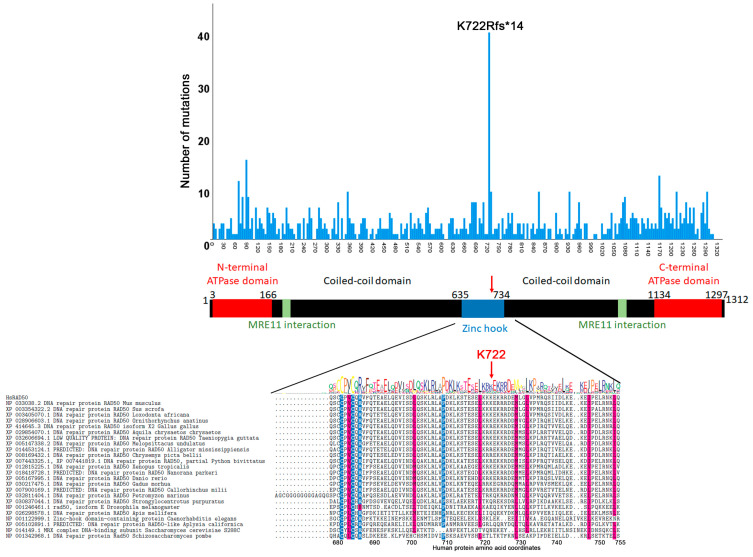
Distribution of RAD50 ORF mutations by amino acid position. The distribution graph is overlayed on a cartoon of RAD50. The K722Rfs*14 mutation falls in the Zn hook domain. The multiple sequence alignment shows that this amino acid is not conserved.

The crystal structure of RAD50 shows that it has two ATPase domains, one at the N-terminus and one at the C-terminus [17,18,20,52,77]. An MRE11 interacting region is found immediately adjacent to the ATPase domains (Figure 4). Two coil-coiled domains allow the protein to fold on itself so that both ATPase domains are close to the MRE11 dimer (Figure 1). The position in the middle of the protein where the two coiled-coil domains fold on themselves is characterized by a zinc hook domain. This zinc hook domain also facilitates dimerization of the RAD50 protein [29,78,79]. Our analysis of COSMIC mutations revealed a hotspot frameshift mutation within the Zn finger domain (K722Rfs*14) [75,80,81,82] (Figure 4). This frameshift mutation also introduces a stop codon causing loss of the C-terminal half of the protein. At least one study in yeast has shown that RAD50 can still associate with MRE11 and NBS1 even in the absence of the hook domain although DNA repair is impaired [83]. Thus, this mutation is likely to affect the function of the complex.

**Figure 5:**
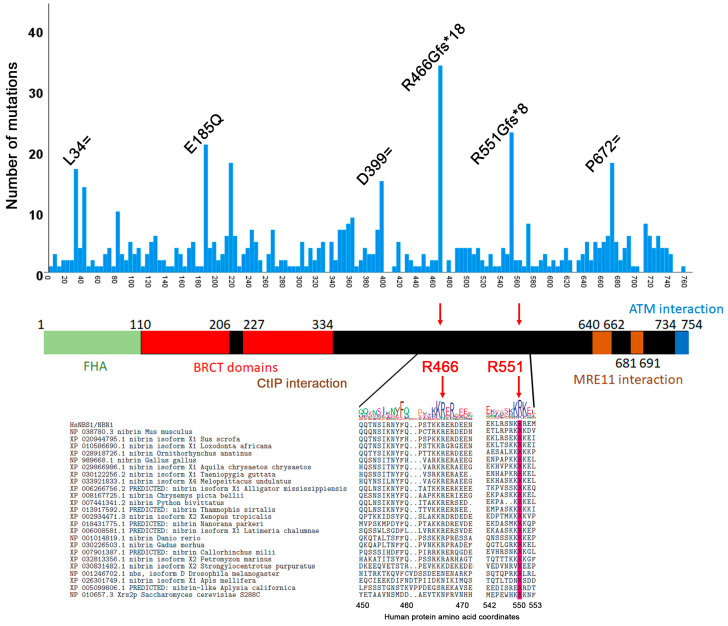
Distribution of NBS1/NBN ORF mutations by amino acid position. The distribution graph is overlayed on a cartoon of NBS1/NBN. The R466Ffs*18 and R551Gfs*8 frameshift mutations fall in the region between the BRCT domains and the MRE11 interaction domains. The multiple sequence alignment shows R551 but not R466 is a conserved amino acid.

NBS1 evolved after eukaryotes and prokaryotes diverged, and there is little conservation between human NBS1/NBN and the *S. cerevisiae* yeast XRS2 homologue (Supplementary Figure S1) [10,84]. In fact, NBS1/NBN shows the lowest level of conservation of all three MRN members [85]. The N-terminus of NBS1 is characterized by two domains that interact with phosphorylated-amino acids [86]: a fork-head associated (FHA) domain that binds phosphoresidues [87] and a BRCA1 C-terminus (BRCT) domain specific for the pSXXF phosphorylated sequence [88] (Figure 5). MRE11 and ATM interaction regions are found in the C-terminus of the protein [41,89]. The N-terminus interacts with CTIP/RBBP8. We found two frameshift hotspots in reported COSMIC mutations (R466Gfs*18 [71,74,75,81,82,90,91,92,93] and R551Gfs*8 [71,74,92,94,95,96,97,98]) that introduce termination codons in the middle region between the BRCT domains and the MRE11 interaction domains (Figure 5).

Several frameshift mutation of MRE11, RAD50 and NBS1 have been independently identified by various studies [56] or detected in large cancer mutation screens (Supplementary Table S1) but to our knowledge the fact that N511Ifs*13, K722Rfs*14, R466Gfs*18, R551Gfs*8 form hotspots have not been shown. A recent report cataloging disease associated MRN mutations also does not list them [59]. The MRE11 N511fs*13 (c.1532del) [69,70,71,72,73,74,75,76] position always produces the same frameshift signature. The RAD50 K722 residue is characterized primarily by the Rfs*14 (c.2165del) frameshift [75,80,81,82,99] but a K722R (c.2165A>G) substitution and a different K722Gfs*5 (c.2164_2165del) frameshift have also been detected [71]. The NBS1/NBN R466Gfs*18 (c.1396del) is the most often occurring mutation [71,74,75,81,82,90,91,92,93] with R466K (c.1397G>A) [100] and R466Kfs*5 (c.1396dup) [92] detected sporadically. Similarly, the NBS1/NBN R551Gfs*8 (c.1651del) mutation is also the most represented [71,74,92,94,95,96,97,98], but an R555K (c.1652G>A) substitution [101] and a different R551Kfs*5 (c.1651dup) [71] frameshift have also been identified.

Some of the other NBS1/NBN mutations have also been described previously [56]. The E185Q mutation is a common polymorphism that occurs in breast, prostate, lung, and several other cancers [59,102,103,104]. The significance of synonymous mutations is debatable, but studies suggest that they may affect mRNA stability and therefore could lead to changes in gene expression [66,105]. COSMIC provides gene expression levels for some TCGA samples. We found one TCGA sample (TCGA-AM-5820-01) that has all three synonymous mutations (L34=, D399= and P672=) and for which NBS1/NBN gene expression levels have been determined. This sample has a gene expression Z-score [106] of 1.307 and is characterized by COSMIC as “normal” (i.e., not under or over-expressed). Thus, we cannot clearly determine the significance of these mutations.

In addition, we analyzed all expression data reported for TCGA samples to determine whether there are changes in expression levels for these genes. We found no statistically significant differences in expression levels (data now shown). However, almost all mutations are heterozygous or the zygocity is unknown/unreported (see below), and the data analyzed does not differentiate between expression of the mutant versus wild type alleles. Therefore, a conclusion about how MRN expression levels could affect the function of the complex cannot be drawn from this study.

### 3.3. Other Non-Sense and Frameshift Mutations

**Figure 6:**
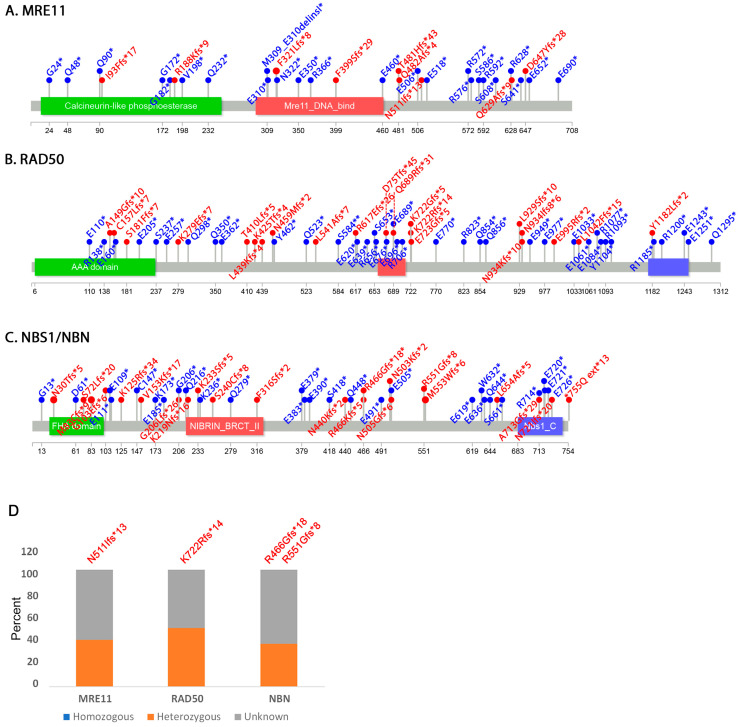
Distribution of other MRN nonsense and frameshift mutations. (**A**–**C**) Lollipops showing distribution of non-sense (blue labels) and frameshift (red labels) mutations. (**D**) Zygosity of the four hotspot frameshift mutations identified in MRE11, RAD50 and NBS1/NBN.

A reasonable prediction is that the closer to the N-terminus a truncating mutation is, the more profound the phenotype would be, presumably because it deletes larger regions of the protein. A visual inspection shows that these truncating mutations are distributed evenly for RAD50 and NBS1/NBN but appear to be less represented in the N-terminus for MRE11 (Figure 6). The possible implications of this is discussed in the next section. Unfortunately, COSMIC has limited information on the zygosity of these mutations, with most of them listed as unknown. For the samples for which zygosity is listed, we found that none of the non-sense or frameshift hotspot mutations are listed as homozygous (Figure 6D). Similarly, none of the other frameshift mutations listed in Figure 6 are homozygous. In fact, the only homozygous mutation is RAD50 Q689* found in a patient with malignant melanoma [107].

The lack of homozygosity of these mutations in cancer samples is not surprising. Although yeast can tolerate deletions of any of the MRN components, homozygous deletion of MRE11, RAD50 and NBS1/NBN has been shown to be embryonic lethal in humans and mice [108,109,110]. Further, heterozygous mutations have been reported in cancer cells and other etiologies for MRE11 [111,112,113,114], RAD50 [115] and most often NBN [110,116,117,118,119,120]. Thus, inactivation of only one allele appears to be sufficient to dysregulate DSB repair which could cause genomic instability.

### 3.4. Correlation between Evolutionary Conservation and Point Mutation Spectrum

Like non-sense and frameshift mutations, missense mutations are also distributed uniformly throughout the ORF of the MRN components. Given the importance of the MRN complex to carcinogenesis, we reasoned that these mutations may be disproportionately affecting functional residues, despite the lack of clear mutational hotspots. Evolutionary conservation is a robust predictor of key functional residues within proteins [121]. We thus investigated the extent to which mutations occur in evolutionary conserved residues using a multiple sequence alignment (MSA) (Table 1). Sequences of MRN complex members were selected from 25–27 species at key evolutionary nodes spanning the range between yeast and human. We find that MRE11 shows the highest level of conservation (113 conserved and 595 non-conserved or 16% conservation) while fewer residues are conserved in RAD50 (136 conserved and 1312 non-conserved or 10.4% conservation) and NBS1/NBN (38 conserved and 716 non-conserved or 5.3% conservation) (Table 1). Remarkably, in MRE11, the Chi-Square statistical analysis found no correlation between residue conservation and the position of COSMIC mutations (*p* = 0.15). Thus, there is no preference for mutations to occur within conserved residues in MRE11. By contrast, mutations in RAD50 and NBS1/NBN show a clear enrichment within conserved residues (*p* = 0.0006 and *p* = 0.0058, respectively). The fact that MRE11 mutations are not over-represented in conserved residues in cancer cells suggests that such mutations may not give an advantage to either cellular transformation or subsequent cancer cell proliferation. Supporting this, MRE11 shows the highest conservation of residues in the N-terminus (Figure S1) and truncating mutations skew towards the C-terminus, far downstream of its cluster of conserved residues (Figure 6A).

### 3.5. Tissue Distribution of Mutations

**Figure 7:**
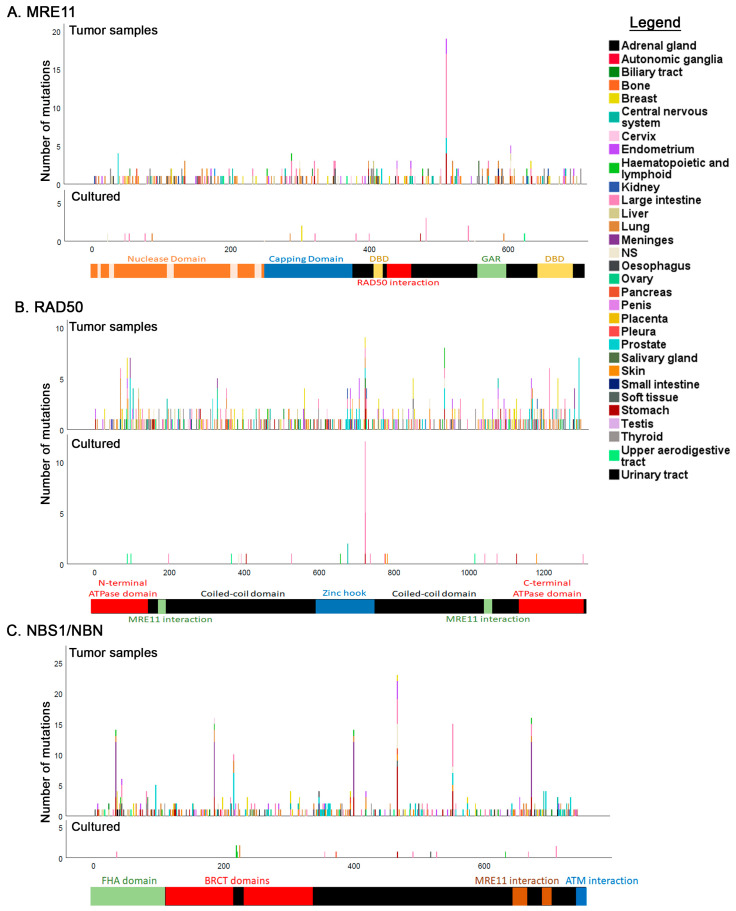
Tissue and positional distribution of MRE11, RAD50 and NBS1/NBN mutations. (**A**–**C**) Tissue and positional distribution of mutations segregated by primary tissue and cell lines.

We next investigated the tissue distribution of MRN mutations. COSMIC segregates most mutations by tissue, though for some the origin is unknown (Supplementary Table S1). The COSMIC database also lists analyzed cancer genomes from tumor samples as well as cultured cells. Because in vitro propagation of cells or cell lines may lead to further cellular transformation we separated these samples from primary tissue samples (Figure 7). For some samples, the origin is not known (Figure S2). This analysis shows that all tissues are characterized by mutations in the MRN genes. There also does not appear to be any tissue specificity for mutations in any region of the three genes with a few exceptions. The MRE11 N511Ifs*13 hotspot is over-represented in the large intestine in tumor samples but does not appear in cell lines (Figure 7A). The RAD50 K722Rfs*14 is also over-represented in the large intestine and interestingly also appears in cell lines (Figure 7B). As mentioned above, it is not immediately clear why this mutational hotspot appears in the large intestine but at least one report showed that MRE11 protein levels are elevated in colorectal cancers [122]. This increase is even more drastic in patients who have received radiation treatment. Mutations in MRE11 have been proposed to increase genomic instability, contributing to cellular transformation and metastasis [122]. Unfortunately, COSMIC does not provide data on the treatment regimen of patients. However, RAD50 protein levels are also elevated in colon cancers due to an increase in double strand breaks [123]. We speculate that disabling the function of MRE11 and RAD50 in these cancers may promote further cellular transformation. The multiple hotspots detected in NBS1/NBN do not appear to correlate with specific tissues.

### 3.6. Mutations in CtIP/RBBP8

**Figure 8:**
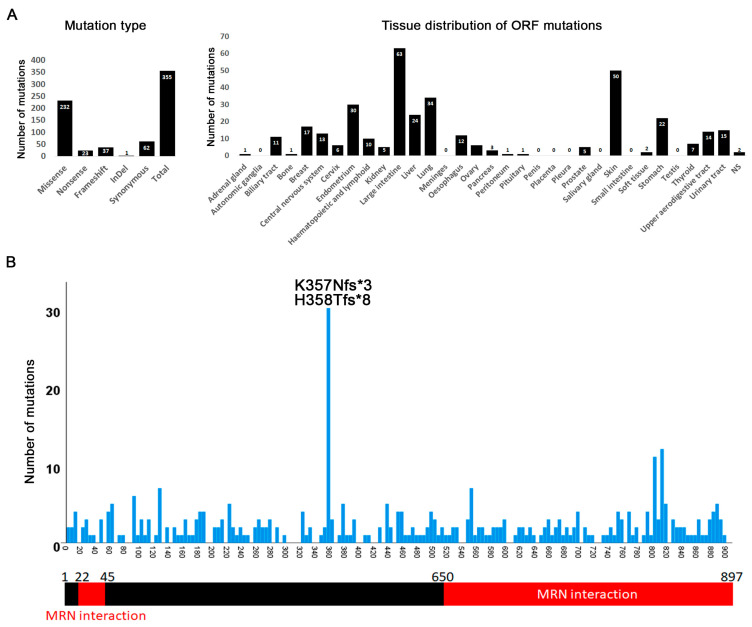
Distribution of CtIP/RBBP8 mutations. (**A**) CtIP mutation and tissue distribution. Intronic and 5′ or 3′ UTR mutations are not included. (**B**) Distribution of CtIP mutations over the ORF of the gene. A “hotspot” is formed by two alleles.

The function of MRN is dependent on CtIP also known as RBBP8 [124]. In humans CtIP interacts with NBS1/NBN and facilitates a complex between MRN and BRCA1 [124,125,126]. Mutations in CtIP may thus resemble those seen for MRE11, RAD50, or NBS1/NBN. We find that the CtIP mutation distribution indeed largely mirrors those of the MRN components (Figure 8A). A truncation hotspot created by two different alleles (K357Nfs*3 and H358Tfs*8) was identified that truncates half of the protein (Figure 8B). These truncating mutations have been previously reported in various studies [70,75,81,82,98,101,127,128,129,130,131,132]. CtIP interacts with different proteins but the MRN interacting regions are found primarily at the C-terminus with a small sequence at the N-terminus [31,32,33]. As such, these truncations abrogate the MRN-interacting region which is predicted to severely affect MRN/CtIP function.

## 4. Conclusions

**Figure 9:**
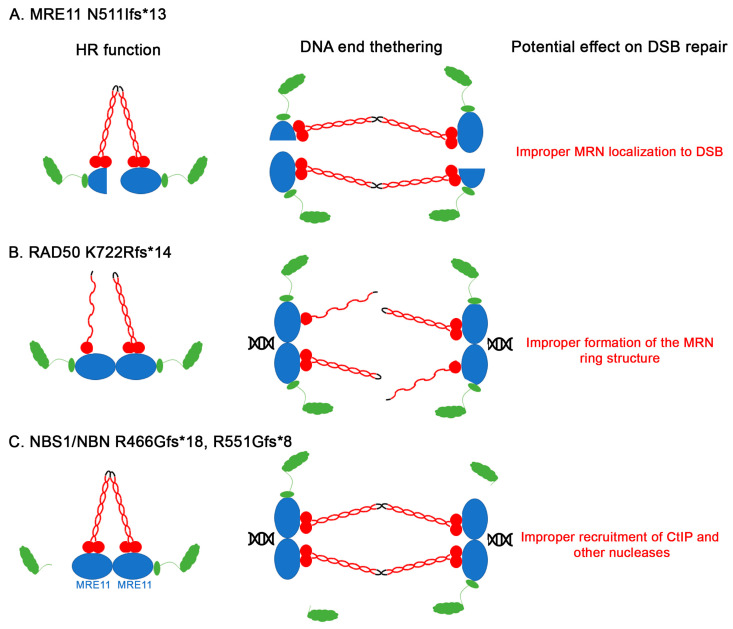
Cartoon models illustrating how the hotspot frameshift mutations disrupt MRN function. (**A**) MRE11 N511LFS*13; (**B**) RAD50 K722Rfs*14; (**C**) NBS1/NBN R466Gfs*18, R551Gfs*8.

Here we identify mutation hotspots in MRE11, RAD50, NBS1/NBN, and CtIP from analyzed cancer data on COSMIC. All hotspots identified truncate the C-terminus of the proteins which almost certainly affects the function of the MRN complex. Because functional MRN requires assembly of two subunits of each protein (Figure 1), one can envision how even heterozygous mutations may still affect function. The MRE11 C-terminal truncation (N511Ifs*13) deletes one of the DNA binding domains and the GAR domain. This mutated protein is likely to affect MRN localization to the DNA break (Figure 9A). The RAD50 K722Rfs*14 falls within the Zinc hook domain and deletes half of the protein (Figure 9B). This will likely compromise the ring structure of the MRN complex. The NBN mutations delete the MRE11 and ATM interacting domains (Figure 9C). Supporting the importance of the intact MRN complex, mutations in its required partner CtIP are predicted to abolish this interaction.

Our evolutionary analysis also shows that mutations in conserved residues of RAD50 and NBS1/NBN are overrepresented in COSMIC, and that this is not the case for MRE11. As all three genes are involved in DSB repair, one interpretation is that mutations abolishing MRE11 function have strong pleiotropic effects and would not be recovered in sampling of patient tumors. By contrast, mutations in conserved residues of RAD50 and NBS1/NBN do not have as great an effect on cellular transformation, allowing them to be represented in samples collected for COSMIC. As the progression of cancer selects for advantageous mutations [133], disruption of some MRE11 conserved residues might not be beneficial. Supporting this, there is evidence that these three genes may also have independent functions [125,134]. Thus, some components of the MRN complex may be more critical for DSB repair than others.

The MRN complex has been extensively studied for several decades. The data presented here highlights differences in the evolution of MRE11, RAD50 and NBS1/NBN and identify key mutation hotspots within cancer samples. Targeted inhibition of the MRN complex as well as certain mutations have been shown to sensitize cells to radiation and chemotherapy, two important cancer treatments [115,135,136,137]. Some potentially interesting future directions include investigating whether some of the mutations described here affect the function of cancer therapeutic agents.

## Figures and Tables

**Table 1:** MRE11, RAD50 and NBS1/NBN residue conservation.

Gene	Residue Conservation from Alignment Data ^1^		Statistical Analysis of Mutations from COSMIC (Chi Square) ^2^				
	Residue Type	Number of Residues	Observed	Expected	Chi-Square Value	Chi-Square Sum	*p*-Value
**MRE11**	Identity	113	52	43.36	1.72	2.05	0.152206
	Non-conserved	595	219	227.64	0.33		
**RAD50**	Identity	136	81	56.58	10.54	11.76	**0.000605**
	Non-conserved	1176	463	487.42	1.22		
**NBN**	Identity	38	30	18.45	7.23	7.61	**0.005805**
	Non-conserved	716	339	350.55	0.38		

^1^ These data represent the numbers (identical vs. non-conserved) from alignment data (Supplementary Figure S1). ^2^ These data represent the COSMIC point mutations that fall within the conserved or non-conserved residues.

## Supplementary Materials

The following are available online at https://www.mdpi.com/2072-6694/12/12/3794/s1, Figure S1: Sequence alignment of MRE11, RAD50 and NBN, Figure S2: Tissue distribution of MRN mutation of unknown origin, Table S1: Summary of MRE11, NBS1 and RAD50 mutations catalogued on COSMIC.
