# Supplementary material for: Mutation Spectra of the MRN (MRE11, RAD50, NBS1/NBN) Break Sensor in Cancer Cells
Source: Cancers (Basel). 2020 Dec 16;12(12):3794. doi: 10.3390/cancers12123794 (PMC7765586; doi:10.3390/cancers12123794)

Mutation spectra of the MRN (MRE11, RAD50, NBS1/NBN) break sensor in cancer cells

Matthew T. McPherson†, Ashton S. Holub†, Aman Y. Husbands and Ruben C. Petreaca*

Department of Molecular Genetics, The Ohio State University, Columbus, OH, 43215, USA.

*Corresponding author: petreaca.1@osu.edu.

†Denotes equal contribution.

Supplementary Table S1. Summary of MRE11, NBS1 and RAD50 mutations catalogued on COSMIC. For some mutations COSMIC provides references which are listed in the right column. For more details, please visit the COSMIC database.

Tissue	MRE11 ORF Mutations	References
Biliary Tract	c.163A>G, c.952C>T	[1]
Breast	p.A440T, p.E451Q, p.Q679K, p.R503C, p.N115D, p.Q218K p.R402K, p.H302Y, p.V429=, p.S632F	[2]
Central Nervous System	p.G683V, p.Q459E, p.V462E, p.D650Y, p.R483Q	[3-5]
Cervix	p.E374Q, p.S641*, p.P318A, p.L274V, p.E466=, p.R503C	N/A
Endometrium	p.G415=, p.S547I, p.V150A, p.K255E, p.M200L, p.R502C, p.T36=, p.D394Y, p.R366Q, p.G172*, p.S681=, p.E494K, p.G285C, p.R708I, p.E518*, p.G58D, p.I497=, p.E460*, p.A440T, p.R188Kfs*9, p.R69M, p.A528T, p.D131N, p.R604C, p.I15L	[6]
Esophagus	p.G172E, p.G371E, p.N699=, p.Q591E, p.R351H, p.S563L, p.V31=	[7, 8] [9, 10]
Hematopoetic and Lymphoid	p.T408K, p.Q477E, p.T627A	[11]
Kidney	p.V304M, p.I125T, p.A595S, p.T658=, p.R628*, p.T627A, p.L44V, p.L94=, p.A4S	[12, 13]
Large Intestine	p.R488H, p.E51K, p.N322*, p.F321S, p.N511Ifs*13, p.E494K, p.A542=, p.N511Ifs*13, p.V458A, p.A440T, p.S586L, p.D53G, p.T481Hfs*43, p.M157V, p.G180C, p.K66=, p.R576*, p.T507=, p.K298N, p.E494K, p.R488H, p.A528=, p.L286=, p.K281N, p.N316=, p.A542=, p.L76=, p.R349W, p.R288H, p.N195=, p.D394Y, p.A539=, p.R380H, p.S649L, p.N617H, p.G114D, p.R380C, p.R366*, p.A390=, p.E77K, p.E460*, p.A528T, p.R572Q, p.F399Sfs*29, p.R380H, p.D517=, p.D236=, p.A47S	[14-19]
Liver	p.D29E, p.E406K, p.E500D, p.G114D, p.G24=, p.G291E, p.I118V, p.I263V, p.I674N, p.K609=, p.L40I, p.L79F, p.N699K, p.Q232*, p.R502S, p.R604S, p.R87=, p.Y82C	[20, 21]
Lung	p.A177G, p.A551T, p.D206Y, p.D368Y, p.D53Y, p.D695Y, p.D86N, p.E10Q, p.E248K, p.E310*, p.E348V, p.E350*, p.G114A, p.G182*, p.G24*, p.G24V, p.G285C, p.G453W, p.G593E/K/R, p.K405E, p.K609N, p.L181F, p.N354I, p.P132H, p.P166L, p.P657T, p.Q48L, p.Q582=, p.R520P, p.R525S, p.R572*, p.S209F, p.S563L, p.T19A, p.T303A, p.V122=, p.V159L, p.Y111S	[22-28]
Ovary	p.A526Gfs*16, p.T199=	[29]
Pancreas	p.D694G, p.N65I	[30]
Pleura	p.K442N	[31]
Prostate	p.A526V, p.D131V, p.I418=, p.I418T, p.N511Ifs*13 p.R572G	[32, 33]
Skin	p.E506*, p.E652K, p.F237= p.G134V/W, p.G182*, p.H397Y, p.H73N, p.I497=, p.K196N, p.K66R, p.L485F, p.L57=, p.P121S, p.P696S, p.Q330K, p.Q357K, p.Q48*, p.R520C p.R606K, p.S273F, p.S563L, p.S676=, p.T603I, p.V519=, p.V643=	[34-37]
Stomach	p.A6T, p.D543=, p.E10G, p.F321Lfs*8, p.F399Sfs*29, p.G134R, p.G154E, p.I395T, p.L473S, p.N511Ifs*13, p.N634S, p.Q477=, p.R364*, p.R604S, p.S156P, p.S586=	[38-40]
Thyroid	p.D130G, p.E27V, p.E403K, p.E406K, p.E652*, p.E690*, p.M84V, p.Q404K, p.R505I, p.R592*, p.R705I	N/A
Upper Aerodigestive Tract	p.A623V, p.E494K, p.G154*, p.S608*, p.V31I, p.Y190H	[41-43]
Urinary Tract	p.F399=, p.H152N, p.H297=, p.N413S, p.Q438E, p.R349L, p.R502G, p.S119L, p.S558F, p.V109=, p.V31G	[44]
Not Specified	p.D695del, p.H22Y, p.R388W	[45, 46]

Tissue	MRE11 Intronic, 5' and 3' UTR Mutations	References
Biliary Tract	c.315-4del, c.*1743del, c.*1743dup, c.1500+1225dup, c.20+643del, c.1017+175del, c.1995-1978dup, c.1500+1225dup, c.1501-178dup, c.1018-730A>G, c.1563+2202dup	N/A
Breast	c.545-641C>A, c.1327-467C>A, c.20+28G>A, c.2071-853dup, c.1564-887G>T, c.660-155A>T, c.1501-467G>T, c.1500+1299A>G, c.1226-1435C>G, c.403-6G>A, c.315-1487C>T, c.544+32A>G, c.1225+916G>A, c.1868-4177C>T, c.2070+3509A>G, c.1784-66A>G, c.315-1422G>T, c.153+2260G>C, c.1500+654A>C, c.1994+1176G>A, c.1225+197T>G, c.1564-3285C>G, c.1994+2772G>T, c.21-112del, c.660-2033A>T, c.153+722C>G, c.2071-2087G>A, c.1501-949A>T, c.545-1101A>G, c.1564-1469_1564-1468del, c.1564-44A>G, c.660-1431G>C, c.315-2931G>A, c.1017+290_1017+308del, c.1500+1225A>C, c.154-614C>G, c.*725_*743del, c.1563+2106G>C, c.2070+2028C>G, c.2070+3170C>A, c.1327-673G>A, c.545-971C>G, c.2071-2932C>T, c.1867+4147T>C, c.2071-91C>T, c.1226-713C>T, c.20+141G>A, c.1994+674C>G	N/A
Central Nervous System	c.*656_*659dup, c.*1743dup, c.2070+1820G>T, c.403-1G>A, c.1868-2284C>T, c.20+50C>A	[5]
Endometrium	c.20+139G>T, c.*119A>C, c.-80C>T, c.20+73G>T, c.1098+1287A>G	N/A
Esophagus	c.1018-217G>A, c.1995-2021G>T, c.845+241G>C, c.2070+716A>T, c.1995-1167A>T, c.1327-115A>G, c.1500+1148A>C, c.659+1066C>T, c.1868-3390G>A, c.1226-627C>G, c.315-264T>G, c.1867+681C>T, c.660-	N/A

	2135C>T, c.1994+1531A>C, c.403-6G>A, c.1995-1061C>G, c.315-1527C>T, c.2070+2905G>T, c.1868-2055T>G, c.545-671A>T, c.315-2918T>G, c.1226-221C>T, c.2071-2821G>A, c.315-2593C>T, c.153+1322C>T	
Hematopoietic and Lymphoid	c.1017+74T>C, c.659+127T>G, c.*726G>A, c.21-798T>A, c.1563+130G>A, c.1867+1890_1867+1891insC, c.1784-66A>G, c.2070+3011del, c.1500+1276T>G, c.1500+1517T>A, c.1563+2208T>G, c.1225+19T>C, c.1500+1519T>A, c.403-84T>G, c.2070+4650C>T, c.2071-945A>C c.*682C>G, c.-120T>C, c.2071-2209C>T, c.1501-596A>G, c.315-96A>G, c.314+1516C>T c.*2667A>G, c.659+1084T>G, c.1564-4304G>A, c.20+28G>A, c.403-6G>A, c.1926+27T>G, c.1017+2T>C c.1867+2757A>C, c.21-279T>A, c.1868-771A>G	[47-50]
Kidney	c.1995-1781A>T, c.1867+1889A>C, c.2070+1724C>T, c.*3832T>G, c.1994+2601C>G, c.1994+2600G>A, c.1783+450del, c.660-2190T>G, c.153+2133C>T, c.1994+993G>T, c.660-531A>T, c.154-1966A>T, c.1500+1519T>A, c.2071-1173A>T, c.1867+230A>T, c.-106+70G>A, c.1995-1867A>G	[51]
Large Intestine	c.1564-134A>T, c.1225+19T>C, c.1225+24T>C, c.2071-167G>T, c.20+73G>T, c.2070+2592C>A, c.314+126A>T, c.*82dup, c.659+96C>A, c.1994+195T>C, c.660-31C>T, c.1563+188C>T, c.1867+1410G>A, c.1099-218C>T, c.1564-22T>G, c.845+25A>G, c.545-163del, c.1868-90A>G, c.1017+2T>C, c.1868-3211A>T, c.1564-1939T>A, c.660-158T>C, c.1098+1378T>G, c.154-76del c.1225+31del, c.*12G>A, c.1099-27C>T, c.1017+174_1017+175del, c.403-64C>T, c.154-71C>T, c.1867+84T>C, c.659+50A>T, c.1098+2T>A, c.402+107C>T, c.1098+1211G>A, c.1326+148T>C, c.846-28A>G, c.1226-568A>T, c.1099-94G>A, c.1225+39A>G, c.1994+48A>C, c.2071-4386T>G, c.845+35A>C, c.153+863C>T, c.845+92G>A, c.1926+170del, c.1868-85_1868-84del	[14, 15, 52, 53]
Liver	c.1226-1298C>T, c.21-601A>G, c.1564-2517A>G, c.1327-120A>T, c.545-797_545-791del, c.1327-114G>T, c.153+1078C>T, c.1564-1058G>A, c.2070+4011G>A, c.659+33G>A, c.1995-1068C>T, c.2071-865C>T, c.1099-1659T>A, c.1564-1748T>C, c.1564-1689A>G, c.1994+1772A>G, c.315-4del, c.2071-709C>A, c.1563+60A>G, c.1563+2029A>G, c.*2419A>G, c.1564-308C>T, c.2071-2732T>C, c.1563+4202G>A, c.1868-1320A>G, c.845+38C>A, c.1995-2003A>G, c.1867+801A>G, c.2070+4072G>C, c.659+19A>G, c.1500+249del, c.545-958A>G, c.1868-2681A>C, c.154-267A>C, c.1927-271A>G, c.1995-111G>T, c.1501-1059T>C, c.1017+30C>G, c.1098+10A>G, c.1867+2850A>G, c.1098+10A>G, c.1500+1467A>G, c.1501-153G>A, c.1017+55C>A, c.544+1038A>G, c.1564-2A>T, c.*660C>T, c.846-448A>G, c.1994+113A>C, c.1868-4162T>C, c.153+23C>G, c.1995-1142T>A, c.1326+353A>G, c.2071-347G>T, c.659+19A>G, c.20+273T>C, c.1563+684C>A, c.1995-1238G>A, c.2070+4662A>G, c.1994+113A>C, c.1098+829A>G, c.154-355G>T, c.1563+3177dup c.1225+850A>G, c.314+6T>C, c.1867+668A>C, c.1867+1888A>C, c.1868-2966G>T, c.659+21A>G, c.1868-4229A>T, c.*2837del, c.846-448A>G, c.1017+391A>G, c.660-356A>G, c.1868-3485G>A, c.153+1071C>T, c.315-145del, c.402+370T>C, c.544+1038A>G, c.545-1057A>G, c.1098+777G>T, c.154-945G>A, c.153+735A>G, c.1564-2594T>C, c.1867+597T>G, c.1564-1238T>G, c.2070+1308G>C, c.402+184A>G, c.545-624C>A, c.1225+167A>G, c.314+6T>C, c.153+288C>G, c.1927-136del, c.1563+3003G>C, c.1098+1683C>G, c.1563+587A>G	[54]
Lung	c.-65G>T, c.545-18A>C, c.1099-1G>T, c.544+34G>T, c.20+37G>T, c.1326+146T>C, c.545-116A>C, c.846-60T>A	[55]
Meninges	c.1868-419G>T	[56]
Ovary	c.314+2799A>G, c.314+13A>G, c.1994+25A>G, c.*656T>C, c.1868-1527A>T, c.315-4del, c.1564-2307A>T, c.1783+505C>T, c.659+981C>G, c.314+13A>G, c.2070+2110T>C, c.1327-2A>G, c.659+709C>A, c.1564-893G>A, c.154-11C>T, c.659+344G>C, c.403-322T>C	[57]
Pancreas	c.402+174T>G, c.1017+1185T>G, c.154-870C>G, c.314+268C>T, c.1995-1799G>C, c.154-1790T>A, c.1867+1885A>C, c.1326+220G>A, c.1017+1185T>G, c.1995-1756C>T, c.1018-31G>A, c.1868-1565T>A, c.660-337T>G, c.1017+1185T>G, c.1099-357G>T, c.1225+419G>T, c.660-1178A>T, c.1500+1344C>T, c.1564-931C>G, c.1868-1565T>A, c.1018-736G>A, c.659+1095A>T, c.1868-1565T>A, c.154-17C>T, c.2071-826G>A, c.1017+1185T>G, c.1563+288C>T, c.659+977T>C, c.545-75T>A	N/A
Prostate	c.1564-4417C>T, c.2071-4640dup, c.1018-958C>A, c.*4355C>G, c.2071-4640dup, c.1867+2223C>G, c.1564-4417C>T, c.1867+3908G>A, c.20+826C>T, c.1225+231G>A, c.2071-4640dup, c.1500+1491G>T, c.154-76del, c.1563+2934A>T, c.1225+231G>A, c.1563+2327T>C, c.1564-4417C>T, c.154-1959C>A, c.1564-4417C>T, c.314+1619A>G, c.1225+390A>G, c.2071-4640dup, c.1500+1517T>A, c.1500+1491G>T, c.2071-1830T>C, c.2070+103C>G, c.154-2367A>T, c.2071-4640dup, c.1926+219A>G, c.21-592G>T, c.314+805T>C, c.1017+1012G>A, c.1994+1886G>T, c.1994+1730G>T, c.1927-326C>T, c.1017+1012G>A, c.2070+4696T>A c.2070+4510T>G, c.154-2302C>G, c.1501-1517_1501-1510delinsGAAAGA, c.314+574G>T, c.2071-4640dup, c.1926+351T>C, c.1500+1491G>T, c.2070+3038G>C	[32]
Skin	c.154-3C>G, c.20+23C>T, c.20+50C>A, c.20+78C>G, c.315-3050T>C, c.2070+3962C>T, c.*94C>T, c.-53C>A	[36]
Soft Tissue	c.1868-904_1868-895del	[58]
Stomach	c.153+51A>G, c.1098+481C>G, c.845+82T>G, c.-47T>A, c.315-5_315-4del, c.1994+46_1994+52del, c.154-76del, c.2071-3745C>T, c.1099-202T>C, c.1326+55T>A, c.153+105C>A, c.315-75A>G, c.1501-337T>G, c.154-76del, c.1226-1408C>A, c.1500+1519T>A, c.1994+99C>A, c.1098+1088A>G, c.154-76del, c.659+617G>T	[59]
Upper Aerodigestive Tract	c.153+1del, c.403-44A>T	[60]
Urinary Tract	c.*44G>C, c.1326+1G>C, c.1326+1G>C	N/A
NS	c.545-1G>A	[45]

Tissue	RAD50 ORF Mutations	References
Autonomic Ganglia	p.N934Kfs*10, p.L622F, p.L475P	[61, 62]
Biliary Tract	p.G382V, p.I505V, p.E75V, p.L84V	[62, 63]
Bone	p.V842A, p.R1260C	[64]
Breast	p.E362Q, p.S373A, p.N395K, p.E444K, p.E463K, p.V497A, p.S560R, p.D675=, p.L719=, p.K722Rfs*14, p.Q74E, p.E75del, p.R759I, p.Q811R, p.L848=, p.R850C, p.R87C, p.E962D, p.E1037Q, p.E1056=, p.R1069I, p.R1077Q, p.M1107I, p.D1120N, p.D1122E, p.T1127S, p.R1198Q, p.T121I, p.D1231E, p.L1237V, p.L151=, p.L251F, p.T26S, p.E291D, p.E328K	[62, 65-71]
Central Nervous System	p.E468G, p.K545E, p.S635N, p.I649=, p.E676*, p.E834K, p.D855G, p.R1077*, p.C1222S, p.E1232D, p.M140I, p.E257*	[5, 62, 72, 73]
Cervix	p.I1227=, p.E283D, p.G41=	[62]
Endometrium	p.I443L, p.I45T, p.E526D, p.S557P, p.S557Y, p.R558M, p.D561E, p.R686K, p.E693D, p.R706*, p.E770*, p.A785V, p.D804Y, p.R807=, p.K838N, p.R884C, p.N953D, p.E962D, p.V101A, p.E1033*, p.E1062D, p.R1069I, p.A1074T, p.R1077Q, p.K1079T, p.R1093*, p.R1098W, p.Y1124D, p.E1171K, p.L1223P, p.D1294N, p.S143Y, p.V150A, p.E205*, p.D21Y, p.E222K	[62, 74]
Esophagus	p.I45V, p.R593T, p.R850H, p.K973M, p.M1140I, p.R1166W	[7, 62, 75]
Hematopoietic and Lymphoid Tissue	p.D358H, p.F396V, p.M415I, p.Y462N, p.Y462F, p.Y462*, p.D549E, p.L615R, p.R656*, p.D69Y, p.M735K, p.V78L, p.P780S, p.T793R, p.A908T, p.L929Sfs*10, p.N934Kfs*10, p.I954V, p.K959=, p.E1061*, p.N1072S, p.F111Y, p.I1165V, p.A1218V, p.P1233S, p.L1276F, p.A188G	[48, 62, 72, 76-82]
Kidney	p.T44=, p.C48=, p.L567M, p.S624G, p.D675Tfs*45, p.R726C, p.N758=, p.T879=, p.M1004L, p.A1074=, p.N1172K, p.T1193A, p.L1208=, p.I1253L, p.R254H, p.K279Efs*7	[13, 51, 62, 83]
Large Intestine	p.S336Y, p.Q350*, p.R352H, p.E362*, p.R385H, p.S389G, p.E390=, p.R391=, p.F396L, p.T410Lfs*5, p.T437A, p.K501R, p.I505V, p.R519H, p.E526D, p.S582=, p.N598=, p.E626K, p.S653*, p.R656Q, p.A657S, p.F66S, p.D69N, p.Q689Rfs*31, p.K721Q, p.K722Gfs*5, p.K722Rfs*14, p.E723Gfs*5, p.R725W, p.R726C, p.R726H, p.R736=, p.K745N, p.L774=, p.V78M, p.S784R, p.A813E, p.I860T, p.R87C, p.E897K, p.E92K, p.N934Kfs*10, p.E949*, p.H955N, p.Y957H, p.M958I, p.E977*, p.R98I, p.E995Rfs*2, p.E1029D, p.E1033*, p.R1038K, p.L1042V, p.K1043N, p.E1062D, p.R1069I, p.A1074S, p.R1077*, p.E1084*, p.H1087N, p.K1089N, p.R1093*, p.R1093Q, p.R1098W, p.E110D, p.I1109T, p.M1111I, p.K1134E, p.H1136R, p.I1146S, p.R1148C, p.R1152Q, p.R1156C, p.R1166Q, p.R1185Q, p.R1198Q, p.R1200Q, p.R120I, p.K1206R, p.L1211F, p.R1214C, p.R1214H, p.T1220=, p.A1246V, p.R1256C, p.F1286L, p.N1292S, p.V1304A, p.R193W, p.Q197=, p.R254C, p.R254H, p.E259*, p.R278Q, p.E291D, p.E302D	[14-17, 52, 62, 84, 85]
Liver	p.Q392H, p.K446E, p.C48F, p.N494K, p.L541P, p.A660S, p.E75K, p.V757=, p.R763H, p.M800V, p.R823L, p.L839F, p.Q856*, p.V101=, p.C102Y, p.H1041Y, p.Q1052K, p.R1077Q, p.L114V, p.V117F, p.S1174L, p.Y1182Lfs*2, p.C1222Y, p.N1236D, p.E1243*, p.E1251*, p.A134S, p.G16E, p.T228A	[20, 86]
Lung	p.L339=, p.L347P, p.R352H, p.Y51C, p.L541Afs*7, p.L566P, p.T61P, p.E620K, p.E639*, p.K651I, p.Q672E, p.D69Y, p.M7T, p.K722R, p.R725W, p.E744K, p.I747K, p.G776S, p.K838T, p.V89G, p.K973=, p.L1042Ffs*15, p.H1071Y, p.N1072S, p.K1126N, p.S1137I, p.R1166Q, p.A1169S, p.K1190=, p.K1190N, p.C1201F, p.A1216=, p.E1232K, p.G124C, p.L1245F, p.G1278R, p.E1281V, p.Q1295*, p.E1298Q, p.I141F, p.V154=, p.T191I, p.R196L, p.D21E, p.T228S, p.S287G, p.V315L, p.R320W	[22, 23, 25-27, 62, 87, 88]
NS	p.R352C, p.N934Ifs*6, p.H158Y, p.R385H, p.A1246S, p.S895F	[45, 46, 62]
Ovary	p.E431D, p.L742S, p.A1248G, p.R1288S, p.R327H	[29, 62]
Pancreas	p.L439Kfs*4, p.I587=, p.T773I, p.K845M, p.R87C, p.N1144S, p.E160K, p.H326Y	[62, 89, 90]
Penis	p.R365Q	[62]
Pleura	p.R278Q, p.A182E	[31]
Prostate	p.A357P, p.Y462C, p.I505V, p.K722Rfs*14, p.L875V, p.N934Ifs*6, p.E1044A, p.A1169T, p.A1169V, p.Y1182C, p.P58S, p.S1137G, p.R1180Q, p.R1185Q, p.E1240=, p.I155S, p.M282I	[34, 38, 62, 91-93]
Salivary Gland	p.K722Rfs*14	[94]
Skin	p.R352C, p.R359C, p.I364=, p.K394E, p.K452=, p.Q523*, p.T536I, p.Q523*, p.T536I, p.P58S, p.A662=, p.Q689*, p.P70L, p.E796=, p.R823Q, p.T867S, p.R87C, p.R87H, p.S895F, p.E897K, p.E920K, p.A940E, p.H955Y, p.L1024S, p.V1050I, p.R1077Q, p.E110K, p.R1098L, p.D1170N, p.R1180=, p.R1200*, p.R1214C, p.L1223F, p.G1226S, p.R1239Q, p.V127=, p.F1286L, p.P250L, p.Q298*	[34-37, 62, 95-100]
Small Intestine	p.R1279H	[62]
Soft Tissue	p.L629Q, p.E239K, p.I272S	[62, 101]
Stomach	p.D417G, p.M502T, p.L594H, p.R617Efs*26, p.K628M, p.C681=, p.V683I, p.K722Rfs*14, p.E723Gfs*5, p.R726C, p.V78M, p.P780=, p.R1077*, p.E1084K, p.D1120E	[38, 62, 102],[75]
Testis	p.M282I	[62]
Thyroid	p.Q465L, p.H559Q, p.E562Q, p.Q589H, p.N598S, p.R850S, p.W1017R, p.K1103=, p.Y1104C, p.Y1104*, p.Q1158H, p.L1237H, p.A171S, p.V203L, p.S237*, p.N310S	[62, 103]
Upper Aerodigestive System	p.R365Q, p.R519H, p.F86V, p.R87H, p.I94L, p.V96L, p.E1015V, p.E1106Q, p.Q207E, p.K238M	[41-43, 62, 104, 105]
Urinary Tract	p.E431Q, p.Q454=, p.L457=, p.E468K, p.E492K, p.L518Q, p.S604F, p.Q685H, p.D69N, p.R79T, p.A810P, p.R823*, p.E869Q, p.K906N, p.Q924K, p.N934Ifs*6, p.V951F, p.E977*, p.M1133I, p.E1161Q,p.S1174L, p.D1177H, p.L1189=, p.R1256C, p.R138*, p.G145R, p.A149Gfs*10, p.E259Q	[44, 62, 71, 106]

Tissue	RAD50 Intronic, 5' and 3' UTR Mutations	References
Autonomic Ganglia	c.*317C>A	N/A
Biliary Tract	c.129+567del, c.213+7825del	N/A
Breast	c.1051+234G>C, c.1452+121A>T, c.1452+547C>T, c.1453-531G>C, c.1794-1269G>A, c.213+166C>A, c.213+242A>T, c.213+3398G>A, c.213+4182T>A, c.213+4441G>A, c.213+5551C>A, c.213+7826A>T, c.213+8000G>C, c.214-4567C>A, c.2207+1283G>C, c.2207+1482G>A, c.2207+889_2207+890insTAATGGTCT, c.2208-1985A>T, c.2208-340G>C, c.2718+1238C>A, c.2718+1382A>C, c.2719-940G>A, c.3036+313G>C, c.3036+997C>G, c.3037-2061A>G, c.3037-2958A>G, c.3037-3170_3037-3169insAT, c.3165-391G>A, c.3165-4A>T, c.3165-692A>T, c.3165-83A>G, c.3389+1671C>T, c.3389+2132T>A, c.3389+2579A>T, c.3389+5475_3389+5476del, c.3389+5496C>T, c.3389+8768G>C, c.3389+8776G>C, c.3390-1106T>C, c.3390-3095C>G, c.3390-3901C>G, c.3390-7535A>T, c.3390-801G>T, c.3390-8448G>T, c.3390-8963A>C, c.3390-9282G>C, c.-355G>C, c.757-2819C>T, c.757-2886C>A, c.757-665G>A, c.757-81C>T, c.757-896A>G	[65]
Central Nervous System	c.*2078G>A, c.*2163T>A, c.2923-1G>A, c.3165-1G>A, c.3619-1G>A	[62, 107]
Endometrium	c.*2826C>G, c.214-6058C>T, c.3390-6351G>A, c.366-875G>C, c.756+479T>C	N/A
Esophagus	c.*286C>T, c.1052-125G>A, c.1052-126dup, c.1052-175C>T, c.213+120G>A, c.213+1357T>G, c.213+4843A>T, c.2207+1514A>C, c.2207+1563A>T, c.2208-3565T>G, c.2718+697C>T, c.3037-2537C>G, c.3389+1132del, c.3389+1593G>T, c.3389+2424T>C, c.3389+6090T>G, c.3389+6656G>T, c.3476-110T>C, c.366-96del, c.757-139_757-138insCTG, c.757-298C>G	[75]
Hematopoietic and Lymphoid Tissue	c.*2898T>C, c.1051+58A>T, c.1245+19A>T, c.1246-198A>G, c.129+141G>A, c.1452+402G>A, c.1636-196T>G, c.214-7850C>T, c.2207+1467T>G, c.2208-3557T>A, c.2524+166T>G, c.2718+644T>A, c.2719-1699A>G, c.3037-1301A>T, c.3037-1343A>T, c.3037-3156_3037-3155del, c.3037-665G>A, c.3164+515A>G, c.3165-528G>A, c.3389+1542C>A, c.3389+1587T>G, c.3389+1589T>G, c.3389+6143C>T, c.3475+62A>G, c.3618+1149G>A, c.3618+857G>T, c.551+123T>C	[48]
Kidney	c.*1249A>G, c.1636-32_1636-31dup, c.214-2566T>A, c.214-992G>A, c.2208-74C>G, c.3036+25C>A, c.3619-687G>T, c.365+460A>G, c.365+997C>G, c.756+1488C>A	[51]
Large Intestine	c.*1596A>T, c.*623_*625del, c.1052-6del, c.1246-136T>C, c.1246-149A>C, c.1636-9del, c.2207+2256G>T, c.2524+192A>C, c.3165-2A>G, c.-32G>A, c.3390-1471T>G, c.3475+322del, c.3475+331T>C, c.3475+38G>T, c.3476-1G>A, c.3619-73A>G, c.366-96del, c.3753-86T>C	[14, 52, 62]
Liver	c.*1078A>G, c.*1132A>G, c.*40C>T, c.*559G>T, c.*996C>T, c.1052-237A>G, c.129+252C>A, c.130-616G>A, c.1452+207A>G, c.1635+12C>A, c.1793+1351A>G, c.1793+771A>G, c.1794-190T>G, c.1794-571T>A, c.213+1794A>G, c.213+2057C>T, c.213+233C>G, c.213+2848A>G, c.213+3061A>G, c.213+3805G>T, c.213+5224T>A, c.213+6024G>A, c.213+6877T>G, c.213+7121A>G, c.213+714T>C, c.213+7599A>G, c.214-125C>T, c.214-285A>T, c.214-3682A>G, c.214-3900C>G, c.214-452A>G, c.214-5308G>T, c.214-5309G>T, c.214-6150A>G, c.2207+3176G>A, c.2207+3409A>C, c.2207+3423T>G, c.2208-1301G>C, c.2208-1526C>T, c.2208-1985A>G, c.2208-2814_2208-2812dup, c.2718+1459G>A, c.2718+960C>G, c.2719-202A>G, c.3036+3101A>G, c.3036+3161G>A, c.3036+3177G>T, c.3037-1197del, c.3037-3102A>G, c.3165-171G>T, c.3165-203A>G, c.3165-210G>A, c.3165-234G>T, c.3165-252A>G, c.3165-33del, c.3165-476G>A, c.3165-511T>G, c.3389+1542C>A, c.3389+3778A>T, c.3389+39A>G, c.3389+609C>T, c.3389+67C>G, c.3389+7035G>T, c.3389+733A>C, c.3389+8796A>G, c.3389+8963A>G, c.3390-2303A>G, c.3390-2A>T, c.3390-3488G>T, c.3390-3542G>T, c.3390-395T>C, c.3390-6651G>T, c.3390-6938G>A, c.3390-7711A>T, c.3475+107A>G, c.3475+128A>G, c.3475+245T>C, c.3475+329C>T, c.3618+762A>T, c.3619-1070A>G, c.3619-1091A>G, c.3619-1123A>G, c.365+1408A>G, c.366-911A>C, c.3752+151C>A, c.3752+294C>T, c.3752+640C>G, c.3753-638T>C, c.551+11C>G, c.551+28G>T, c.756+1218A>T, c.756+1692G>C, c.756+1738G>A, c.756+1854A>G, c.756+2044A>G, c.756+3120A>C, c.756+3268T>A, c.756+3587A>G, c.756+777A>G, c.757-2456A>G, c.757-790G>A	N/A
Lung	c.1052-16A>T, c.1635+21C>G, c.1636-1G>T, c.214-151A>G, c.2525-152A>C, c.2719-154A>C, c.2922+1G>A, c.3476-110T>C, c.3618+95G>C, c.551+78A>G, c.552-1G>T, c.885+11G>A	[23, 45, 62]
Ovary	c.*1029C>T, c.213+2875T>G, c.213+3829A>G, c.2208-3629C>T, c.2718+322G>T, c.2719-1568T>C, c.3389+5851A>G, c.3389+6657G>C, c.3390-8621T>C, c.3618+1G>A, c.365+1582T>G, c.757-1665A>T	[62]
Pancreas	c.*1269C>T, c.*2842C>A, c.1051+292C>G, c.1453-528A>T, c.1794-1261A>C, c.213+2301T>C, c.213+4334G>A, c.213+6024G>A, c.214-2611G>A, c.214-7675A>G, c.2208-2840A>G, c.2208-389A>C, c.2718+590G>C, c.3165-145T>C, c.3389+1542C>A, c.3389+1546A>C, c.3389+1591T>G, c.3389+2639C>T, c.3389+9348G>T, c.3390-2082A>G, c.3390-3857C>T, c.3390-8020C>T, c.3475+323C>T, c.3618+918G>T, c.366-1410C>T, c.3752+481G>A, c.3753-145G>A, c.756+1280C>T, c.756+3006G>A, c.757-332A>T, c.757-3646C>T	N/A
Prostate	c.*1027C>T, c.1246-4_1248del, c.1636-14T>G, c.1794-852T>G, c.213+3944C>A, c.213+5936A>G, c.213+5T>G, c.214-231A>G, c.2207+3143G>T, c.2719-1619G>A, c.2719-582C>G, c.3036+112G>A, c.3036+2454C>T, c.3036+2530A>G, c.3037-1086C>T, c.3165-476G>A, c.3389+1077A>G, c.3389+1589T>G, c.3389+1593G>T, c.3389+4511del, c.3389+6097del, c.3389+7466G>A, c.3389+9123A>C, c.3389+9395A>T, c.3390-4270G>A, c.3390-4882A>G, c.3390-6843G>C, c.3390-7019C>A, c.3390-8256dup, c.366-1663del, c.366-96del, c.366-96dup, c.756+1553G>T, c.756+3030G>C, c.756+448_756+450del, c.756+978G>A, c.757-103C>T, c.757-994T>A	[62, 108]
Skin	c.*2958A>C, c.130-520T>G, c.1453-560C>T, c.1636-1G>A, c.1453-560C>T, c.1636-1G>A, c.3036+1994_3036+1998del, c.3389+5426C>T, c.3619-82G>T, c.552-2A>G, c.757-1971C>T, c.757-2597A>G	[36, 109]
Soft Tissue	c.214-7703T>A	[58]

Stomach	c.1636-9del, c.2207+2256G>T, c.2525-26T>C, c.-32G>T, c.3390-5944A>G, c.3390-7436C>A, c.885+11G>A	[59, 110]
Thyroid	c.1793+879G>T	N/A
Upper Aerodigestive System	c.3390-3042del, c.3752+29G>C	N/A

Tissue	NBN ORF Mutations	References
Autonomic Ganglia	p.S195R	[111]
Biliary Tract	p.T178I, p.M83Cfs*9	[62]
Bone	p.V314M	[64]
Breast	p.C119S, p.L302V, p.E36D, p.D586Y, p.I290F, p.E610K, p.K236*, p.K29R, p.H540N, p.S213L, p.H540Y, p.S123P, p.K236N	[62, 112, 113]
Central Nervous System	p.L723P, p.L302F, p.E610K, p.V243I, p.E726K, p.V314M, p.G246D, p.S486F	[62, 114]
Cervix	p.E185K	N/A
Endometrium	p.P354S, p.S355I, p.S418*, p.R43Q, p.S447Y, p.R466Gfs*18, p.T526I, p.E543K, p.P6S, p.R624C, p.E636*, p.L654Afs*5, p.F685V, p.R714*, p.A740V, p.V155M, p.I166T, p.Q216*, p.R218I, p.T268M, p.D284G	[62]
Esophagus	p.V346M, p.R43Q, p.I455V, p.S499=, p.R551Gfs*8, p.K635N, p.V691L, p.*755Qext*13, p.M83Cfs*9, p.G100A, p.N142S, p.P191A, p.D211Y	[9, 10, 62, 75, 115-117]
Hematopoietic and Lymphoid Tissue	p.L34=, p.D399=, p.I41S, p.P672=, p.K105E, p.E185Q, p.E185*, p.K219Nfs*16, p.K219N, p.Q220E	[80, 118]
Kidney	p.M389V, p.I439V, p.E619K, p.Y679H, p.L68=, p.A713Gfs*29, p.N731Ifs*20, p.K125Rfs*34, p.G206Lfs*26, p.L230F, p.S240Cfs*8	[62, 119, 120]
Large Intestine	p.L34=, p.V346=, p.I35T, p.L352R, p.S355=, p.N359S, p.Y363=, p.E383*, p.D399=, p.S411C, p.Y429H, p.R43Q, p.R466Gfs*18, p.L490*, p.P498L, p.E505*, p.E512Q, p.D519G, p.N520S, p.T526I, p.S530Y, p.R551Gfs*8, p.R551K, p.M553Wfs*6, p.V556=, p.V608I, p.S647G, p.L654Afs*5, p.K653N, p.S661*, p.S667P, p.P672=, p.A696=, p.D70Y, p.A710S, p.L723=, p.Q732=, p.L739P, p.E81K, p.M83Cfs*9, p.R89Q, p.A131=, p.L135P, p.R169C, p.E185Q, p.R215W, p.R218I, p.V25I, p.P266=, p.N30Tfs*5, p.L302I, p.A313V, p.F316L, p.Y322C, p.G327C,	[14, 16, 17, 62, 72, 84, 85, 121]
Liver	p.R43*, p.S488=, p.F52L, p.T570R, p.Q616K, p.D677G, p.E728=, p.V153=, p.P15=, p.E23K, p.Q234H, p.H235Q, p.V242L, p.P325T	[62]
Lung	p.P338L, p.L34=, p.K351R, p.A356T, p.A365P, p.D373H, p.W375C, p.E390*, p.D399=, p.S418*, p.I455=, p.S486=, p.E512G, p.V54=, p.K549E, p.K550I, p.E561K, p.L574I, p.D646Y, p.V65A, p.L656M, p.R660=, p.I664M, p.P672=, p.N684K, p.F685I, p.N716D, p.T717A, p.E721*, p.E726*, p.V729L, p.E737K, p.A8T, p.S88T, p.G103Efs*6, p.G103V, p.S118C, p.V122=, p.G124R, p.E185Q, p.D201V, p.P203L, p.G206*, p.R215W, p.R215L, p.F222L, p.F222=, p.G224V, p.G224A, p.G245A, p.R250M, p.F262L, p.P266L, p.G267A, p.D284H, p.K287=, p.G301S, p.K320E, p.Q326E, p.I33F	[22-25, 62, 72, 87, 88, 122, 123]
NS	p.R466Gfs*18, p.P191L, p.R551Gfs*8, p.N503Y, p.P572S	[45, 62]
Ovary	p.I41=, p.D272N	N/A
Pancreas	p.Y363H, p.A372V, p.R394I, p.R466Gfs*18, p.R592S, p.S667C, p.E185Q,	[62, 90, 124, 125]
Penis	p.T337N, p.V157F, p.K715N	[62]
Placenta	p.E658K	[62]
Pleura	p.T657A	[31]
Prostate	p.T361K, p.Y363=, p.R551Gfs*8, p.K642R, p.R714L, p.Q732K, p.K82E	[32, 62, 92, 126, 127]
Salivary Gland	p.P694T	[128]
Skin	p.L342H, p.A356V, p.S387F, p.R394I, p.S418*, p.R466K, 25759019, p.D469N, p.E491*, p.H540Y, p.R551=, p.L574S, p.Q644*, p.Q644=, p.V65I, p.S669F, p.D121N, p.L165H, p.P170L, p.S186F, p.S207N, p.L297=, p.V314M	[34-37, 62, 129, 130]
Small Intestine	p.V346M	[62]
Soft Tissue	p.P338S, p.P354L, p.R466Gfs*18, p.N517K, p.V153Kfs*17, p.W2C	[62, 131-133]
Stomach	p.T337I, p.S432P, p.K464E, p.R466Gfs*18, p.R551Gfs*8, p.L563F, p.D596N, p.I675M, p.Q84H, p.K125E, p.A131D, p.V172=, p.L181M, p.R215=, p.S240=, p.Y322H	[38, 59, 62]
Testis	p.N640S	[62]
Thyroid	p.P381S, p.I426M, p.L656=, p.R660I, p.E728D, p.E81K, p.E109*, p.E185Q, p.R215W	[62, 103, 134]
Upper Aerodigestive System	p.L377F, p.S418*, p.P461L, p.T485M, p.T717S, p.R17T	[43, 60, 135]
Urinary Tract	p.E36A, p.N37T, p.E379K, p.R394G, p.S40=, p.R43Q, p.A46V, p.S499L, p.E510Q, p.R551Kfs*5, p.S615F, p.R670T, p.D677N, p.H712Y, p.E737Q, p.R89Q, p.I162V, p.E254K, p.F263L	[44, 62]

Tissue	NBN Intronic, 5' and 3' UTR Mutations	References
Adrenal Gland	c.481-1G>C	[136]
Biliary Tract	c.896+1769del, c.1397+747del, c.2070+109_2070+111del, c.1914+41C>T, c.897-983dup, c.*502A>G, c.480+1026T>A, c.1397+747dup, c.1397+747del, c.584+2906A>T	N/A
Bone	c.2071-2A>G	[62]
Breast	c.1397+314C>G, c.320+212C>T, c.2070+335C>T, c.2070+607C>T, c.2070+986C>G, c.994+637G>T, c.2184+2131_2184+2139del, c.2185-1483C>T, c.896+220C>T, c.*2065C>T, c.1846-1391T>C, c.584+1627G>A, c.1846-1559C>T, c.1397+266G>A, c.897-732C>T, c.2185-1483C>T, c.2184+2T>A, c.2184+245T>C, c.897-1646C>G, c.*2065C>T, c.1124+54G>C, c.1397+266G>A, c.585-399G>T, c.2185-1209G>A, c.995-2016C>G, c.1845+418T>G, c.2185-1031C>G, c.584+26A>G, c.321-47C>T, c.1124+663T>A, c.1397+656C>G, c.896+1709C>T, c.2184+2709T>A, c.1846-542G>A, c.2071-84C>A, c.*1017C>T, c.480+95A>G, c.2235-1G>A, c.994+595G>C, c.584+3379G>C, c.480+398G>T, c.1124+444C>T, c.480+589C>T, c.897-711C>T, c.1846-529C>G,	[62, 70]
Central Nervous System	c.1124+292G>A,	N/A
Endometrium	c.2184+882C>T	N/A
Esophagus	c.1398-10del, c.171+55A>G, c.2185-2670G>C, c.585-930A>C, c.994+843T>G, c.995-1G>A, c.995-780C>G	[75]
Eye	c.*724_*725insTG	[137]
Hematopoietic and Lymphoid Tissue	c.*486T>A, c.1124+18C>T, c.1125-79C>A, c.1397+259del, c.1397+72A>T ,c.1846-23T>A, c.1846-2424T>G, c.1846-2574_1846-2569del, c.1846-402T>C, c.1846-644G>A, c.1915-7A>G, c.2071-2A>G, c.2184+1494A>T, c.2184+1686T>C, c.2185-122T>C, c.2185-1393C>T, c.2185-655C>T, c.2185-919A>G, c.2234+687A>T, c.584+2682T>A, c.703-18G>A, c.896+728_896+730del	[138]
Kidney	c.1915-1G>A, c.2185-121C>T, c.2185-1733G>A, c.585-1779G>T, c.585-2196G>A, c.585-492G>A, c.994+2722T>A, c.995-511G>A	N/A
Large Intestine	c.*1977T>C, c.1124+125G>A, c.1397+10dup, c.1397+41del, c.1398-10del, c.1914+74del, c.1915-162A>G, c.2184+123G>A, c.2185-1385G>T, c.2185-1387G>T, c.2185-577T>G, c.2235-433G>A, c.2235-4G>C, c.38-18A>T, c.38-1G>T, c.38-27C>A, c.585-117del, c.703-174G>A, c.896+1065G>A, c.896+517C>T, c.896+63del, c.897-84del	[14, 62, 139, 140]
Liver	c.*1167A>G, c.*143A>G, c.1124+66A>G, c.1125-164A>G, c.1398-154A>G, c.1398-457A>G, c.1398-483A>T, c.172-229G>A, c.1845+1571G>A, c.1845+2595C>G, c.1846-2248T>A, c.1846-75C>G, c.1914+318C>T, c.1915-122A>G, c.2071-22T>C, c.2184+1518G>T, c.2184+2174C>A, c.2184+689A>G, c.2185-93del, c.2234+495_2234+498del, c.37+79C>T, c.38-241T>G, c.38-249A>G, c.480+1080dup, c.481-110C>T, c.481-191A>G, c.584+1367G>T, c.584+160A>G, c.584+217T>C, c.584+409A>G, c.585-2796G>T, c.585-3347T>C, c.702+12A>T, c.703-232C>A, c.-79G>C, c.896+117G>T, c.896+33C>A, c.896+75C>G, c.897-1242G>C, c.994+1221A>T, c.994+2676dup, c.995-3C>A, c.995-766C>A	N/A
Lung	c.*1524G>T, c.1124+18C>T, c.1124+37C>T, c.1124+91C>A, c.1398-152A>C, c.172-578G>T, c.1845+2085T>C, c.1915-7A>G, c.2070+1G>T, c.2071-30A>T, c.-22C>G, c.320+1G>T, c.37+20C>A, c.38-1G>T, c.481-1G>C, c.896+120G>C	[23, 62, 141]
NS	c.1845+2T>C	[62]
Ovary	c.1125-960T>C, c.2184+1545A>C, c.2185-1665A>G, c.481-1051G>T, c.584+2922T>A, c.584+789A>G, c.897-1363A>G, c.897-425C>G, c.995-1212C>G, c.995-1263T>G, c.995-808C>A	N/A
Pancreas	c.*1320A>G, c.*2163C>T, c.1124+1056A>C, c.1124+41C>G, c.1124+81C>G, c.1397+230G>A, c.1398-10T>A, c.1398-728G>A, c.2070+1110C>T, c.2184+1740C>A, c.2185-1393C>T, c.2185-1646T>G, c.2185-641C>T, c.2234+176G>A, c.37+163C>T, c.38-19A>T, c.481-1205C>A, c.584+2743G>T, c.585-2656G>A, c.896+1170G>T, c.896+2403G>T, c.896+2404G>T, c.897-170G>A, c.994+1436G>T, c.994+2231T>A, c.994+273G>T, c.994+513A>G, c.994+842A>T, c.995-1794C>T, c.995-258C>G, c.995-264A>T, c.995-336C>T	N/A
Prostate	c.1124+761G>A, c.1398-10T>A, c.1846-24G>T, c.2070+832A>G, c.2184+840G>C, c.2185-1374_2185-1373del, c.2185-2449G>A, c.2234+180C>T, c.2234+395A>G, c.480+575G>A, c.480+80_480+81insT, c.584+1255A>G, c.584+147_584+150del,c.584+1725C>G, c.584+2599C>T, c.584+894dup, c.585-117del, c.994+266_994+267insCCA, c.995-1074T>G, c.995-1798_995-1796del, c.995-403A>G	[32, 33]
Skin	c.1125-643_1125-642insTC, c.1398-1G>A, c.1915-7A>G, c.481-741T>C, c.896_896+1delinsAA, c.994+513A>T	[34, 36]
Soft Tissue	c.1397+46del, c.2071-61A>T, c.-26G>A, c.703-18G>A, c.896+10dup	[132, 133]
Stomach	c.2071-752G>A. c.2071-839G>A, c.2184+1455T>C, c.38-20T>A, c.584+292A>G, c.703-149G>A, c.896+100G>T	N/A
Thyroid	c.897-2A>T	[103]
Upper Aerodigestive System	c.1914+2T>A, c.2235-563G>T, c.481-766A>G	[62]
Urinary Tract	c.2071-13_2077del, c.2184+6T>C,	[142]

References

1. Wardell, C.P., et al., *Genomic characterization of biliary tract cancers identifies driver genes and predisposing mutations.* J Hepatol, 2018. **68**(5): p. 959-969.
2. Malhotra, A., et al., *Ploidy-Seq: inferring mutational chronology by sequencing polyploid tumor subpopulations.* Genome Med, 2015. **7**(1): p. 6.
3. Nomura, M., et al., *Distinct molecular profile of diffuse cerebellar gliomas.* Acta Neuropathol, 2017. **134**(6): p. 941-956.
4. Killela, P.J., et al., *The genetic landscape of anaplastic astrocytoma.* Oncotarget, 2014. **5**(6): p. 1452-7.
5. Patil, V., J. Pal, and K. Somasundaram, *Elucidating the cancer-specific genetic alteration spectrum of glioblastoma derived cell lines from whole exome and RNA sequencing.* Oncotarget, 2015. **6**(41): p. 43452-71.
6. Le Gallo, M., et al., *Exome sequencing of serous endometrial tumors identifies recurrent somatic mutations in chromatin-remodeling and ubiquitin ligase complex genes.* Nat Genet, 2012. **44**(12): p. 1310-5.
7. Gao, Y.B., et al., *Genetic landscape of esophageal squamous cell carcinoma.* Nat Genet, 2014. **46**(10): p. 1097-102.
8. Sawada, G., et al., *Genomic Landscape of Esophageal Squamous Cell Carcinoma in a Japanese Population.* Gastroenterology, 2016. **150**(5): p. 1171-1182.
9. Cheng, C., et al., *Genomic analyses reveal FAM84B and the NOTCH pathway are associated with the progression of esophageal squamous cell carcinoma.* Gigascience, 2016. **5**: p. 1.
10. Zhang, L., et al., *Genomic analyses reveal mutational signatures and frequently altered genes in esophageal squamous cell carcinoma.* Am J Hum Genet, 2015. **96**(4): p. 597-611.
11. Ljungstrom, V., et al., *Whole-exome sequencing in relapsing chronic lymphocytic leukemia: clinical impact of recurrent RPS15 mutations.* Blood, 2016. **127**(8): p. 1007-16.
12. Dietz, S., et al., *Patient-specific molecular alterations are associated with metastatic clear cell renal cell cancer progressing under tyrosine kinase inhibitor therapy.* Oncotarget, 2017. **8**(43): p. 74049-74057.
13. Durinck, S., et al., *Spectrum of diverse genomic alterations define non-clear cell renal carcinoma subtypes.* Nat Genet, 2015. **47**(1): p. 13-21.
14. Giannakis, M., et al., *Genomic Correlates of Immune-Cell Infiltrates in Colorectal Carcinoma.* Cell Rep, 2016. **15**(4): p. 857-865.
15. Cancer Genome Atlas, N., *Comprehensive molecular characterization of human colon and rectal cancer.* Nature, 2012. **487**(7407): p. 330-7.
16. Giannakis, M., et al., *RNF43 is frequently mutated in colorectal and endometrial cancers.* Nat Genet, 2014. **46**(12): p. 1264-6.
17. Mouradov, D., et al., *Colorectal cancer cell lines are representative models of the main molecular subtypes of primary cancer.* Cancer Res, 2014. **74**(12): p. 3238-47.
18. Kim, T.M., et al., *Clonal origins and parallel evolution of regionally synchronous colorectal adenoma and carcinoma.* Oncotarget, 2015. **6**(29): p. 27725-35.
19. Liu, Z., et al., *The landscape of somatic mutation in sporadic Chinese colorectal cancer.* Oncotarget, 2018. **9**(44): p. 27412-27422.
20. Jhunjhunwala, S., et al., *Diverse modes of genomic alteration in hepatocellular carcinoma.* Genome Biol, 2014. **15**(8): p. 436.
21. Kan, Z., et al., *Whole-genome sequencing identifies recurrent mutations in hepatocellular carcinoma.* Genome Res, 2013. **23**(9): p. 1422-33.

22. Imielinski, M., et al., *Mapping the hallmarks of lung adenocarcinoma with massively parallel sequencing.* Cell, 2012. **150**(6): p. 1107-20.
23. McMillan, E.A., et al., *Chemistry-First Approach for Nomination of Personalized Treatment in Lung Cancer.* Cell, 2018. **173**(4): p. 864-878 e29.
24. Liu, J., et al., *Genome and transcriptome sequencing of lung cancers reveal diverse mutational and splicing events.* Genome Res, 2012. **22**(12): p. 2315-27.
25. George, J., et al., *Comprehensive genomic profiles of small cell lung cancer.* Nature, 2015. **524**(7563): p. 47-53.
26. Seo, J.S., et al., *The transcriptional landscape and mutational profile of lung adenocarcinoma.* Genome Res, 2012. **22**(11): p. 2109-19.
27. Li, C., et al., *Whole Exome Sequencing Identifies Frequent Somatic Mutations in Cell-Cell Adhesion Genes in Chinese Patients with Lung Squamous Cell Carcinoma.* Sci Rep, 2015. **5**: p. 14237.
28. Wagner, A.H., et al., *Recurrent WNT pathway alterations are frequent in relapsed small cell lung cancer.* Nat Commun, 2018. **9**(1): p. 3787.
29. Cancer Genome Atlas Research, N., *Integrated genomic analyses of ovarian carcinoma.* Nature, 2011. **474**(7353): p. 609-15.
30. Murphy, S.J., et al., *Genetic alterations associated with progression from pancreatic intraepithelial neoplasia to invasive pancreatic tumor.* Gastroenterology, 2013. **145**(5): p. 1098-1109 e1.
31. Bueno, R., et al., *Comprehensive genomic analysis of malignant pleural mesothelioma identifies recurrent mutations, gene fusions and splicing alterations.* Nat Genet, 2016. **48**(4): p. 407-16.
32. Robinson, D., et al., *Integrative clinical genomics of advanced prostate cancer.* Cell, 2015. **161**(5): p. 1215-1228.
33. Kumar, A., et al., *Substantial interindividual and limited intraindividual genomic diversity among tumors from men with metastatic prostate cancer.* Nat Med, 2016. **22**(4): p. 369-78.
34. Sharpe, H.J., et al., *Genomic analysis of smoothened inhibitor resistance in basal cell carcinoma.* Cancer Cell, 2015. **27**(3): p. 327-41.
35. Krauthammer, M., et al., *Exome sequencing identifies recurrent somatic RAC1 mutations in melanoma.* Nat Genet, 2012. **44**(9): p. 1006-14.
36. Pickering, C.R., et al., *Mutational landscape of aggressive cutaneous squamous cell carcinoma.* Clin Cancer Res, 2014. **20**(24): p. 6582-92.
37. South, A.P., et al., *NOTCH1 mutations occur early during cutaneous squamous cell carcinogenesis.* J Invest Dermatol, 2014. **134**(10): p. 2630-2638.
38. Wang, K., et al., *Whole-genome sequencing and comprehensive molecular profiling identify new driver mutations in gastric cancer.* Nat Genet, 2014. **46**(6): p. 573-82.
39. Liu, J., et al., *Integrated exome and transcriptome sequencing reveals ZAK isoform usage in gastric cancer.* Nat Commun, 2014. **5**: p. 3830.
40. Wang, K., et al., *Exome sequencing identifies frequent mutation of ARID1A in molecular subtypes of gastric cancer.* Nat Genet, 2011. **43**(12): p. 1219-23.
41. Martin, D., et al., *The head and neck cancer cell oncogenome: a platform for the development of precision molecular therapies.* Oncotarget, 2014. **5**(19): p. 8906-23.
42. Fadlullah, M.Z., et al., *Genetically-defined novel oral squamous cell carcinoma cell lines for the development of molecular therapies.* Oncotarget, 2016. **7**(19): p. 27802-18.
43. Stransky, N., et al., *The mutational landscape of head and neck squamous cell carcinoma.* Science, 2011. **333**(6046): p. 1157-60.

44. Guo, G., et al., *Whole-genome and whole-exome sequencing of bladder cancer identifies frequent alterations in genes involved in sister chromatid cohesion and segregation.* Nat Genet, 2013. **45**(12): p. 1459-63.
45. Van Allen, E.M., et al., *The genetic landscape of clinical resistance to RAF inhibition in metastatic melanoma.* Cancer discovery, 2014. **4**(1): p. 94-109.
46. Dutton-Regester, K., et al., *Melanomas of unknown primary have a mutation profile consistent with cutaneous sun-exposed melanoma.* Pigment Cell Melanoma Res, 2013. **26**(6): p. 852-60.
47. Puente, X.S., et al., *Whole-genome sequencing identifies recurrent mutations in chronic lymphocytic leukaemia.* Nature, 2011. **475**(7354): p. 101-5.
48. McGirt, L.Y., et al., *Whole-genome sequencing reveals oncogenic mutations in mycosis fungoides.* Blood, 2015. **126**(4): p. 508-19.
49. Jiang, L., et al., *Exome sequencing identifies somatic mutations of DDX3X in natural killer/T-cell lymphoma.* Nat Genet, 2015. **47**(9): p. 1061-6.
50. Welch, J.S., et al., *The origin and evolution of mutations in acute myeloid leukemia.* Cell, 2012. **150**(2): p. 264-78.
51. Sato, Y., et al., *Integrated molecular analysis of clear-cell renal cell carcinoma.* Nat Genet, 2013. **45**(8): p. 860-7.
52. van de Wetering, M., et al., *Prospective derivation of a living organoid biobank of colorectal cancer patients.* Cell, 2015. **161**(4): p. 933-45.
53. Yang, J., et al., *Genome landscapes of rectal cancer before and after preoperative chemoradiotherapy.* Theranostics, 2019. **9**(23): p. 6856-6866.
54. Totoki, Y., et al., *High-resolution characterization of a hepatocellular carcinoma genome.* Nat Genet, 2011. **43**(5): p. 464-9.
55. Yin, S., et al., *Exome sequencing identifies frequent mutation of MLL2 in non-small cell lung carcinoma from Chinese patients.* Sci Rep, 2014. **4**: p. 6036.
56. Bi, W.L., et al., *Genomic landscape of high-grade meningiomas.* NPJ Genom Med, 2017. **2**.
57. Jones, S., et al., *Low-grade serous carcinomas of the ovary contain very few point mutations.* J Pathol, 2012. **226**(3): p. 413-20.
58. Behjati, S., et al., *Recurrent PTPRB and PLCG1 mutations in angiosarcoma.* Nat Genet, 2014. **46**(4): p. 376-379.
59. Chong, I.Y., et al., *The genomic landscape of oesophagogastric junctional adenocarcinoma.* J Pathol, 2013. **231**(3): p. 301-10.
60. Al-Hebshi, N.N., et al., *Exome sequencing of oral squamous cell carcinoma in users of Arabian snuff reveals novel candidates for driver genes.* Int J Cancer, 2016. **139**(2): p. 363-72.
61. Pugh, T.J., et al., *The genetic landscape of high-risk neuroblastoma.* Nat Genet, 2013. **45**(3): p. 279-84.
62. Zehir, A., et al., *Mutational landscape of metastatic cancer revealed from prospective clinical sequencing of 10,000 patients.* Nat Med, 2017. **23**(6): p. 703-713.
63. Li, M., et al., *Whole-exome and targeted gene sequencing of gallbladder carcinoma identifies recurrent mutations in the ErbB pathway.* Nat Genet, 2014. **46**(8): p. 872-6.
64. Crompton, B.D., et al., *The genomic landscape of pediatric Ewing sarcoma.* Cancer Discov, 2014. **4**(11): p. 1326-41.
65. Maresca, L., et al., *Functional Interaction Between BRCA1 and DNA Repair in Yeast May Uncover a Role of RAD50, RAD51, MRE11A, and MSH6 Somatic Variants in Cancer Development.* Front Genet, 2018. **9**: p. 397.
66. Guerini-Rocco, E., et al., *The repertoire of somatic genetic alterations of acinic cell carcinomas of the breast: an exploratory, hypothesis-generating study.* J Pathol, 2015. **237**(2): p. 166-78.

67. Stephens, P.J., et al., *The landscape of cancer genes and mutational processes in breast cancer.* Nature, 2012. **486**(7403): p. 400-4.
68. Ferrari, A., et al., *A whole-genome sequence and transcriptome perspective on HER2-positive breast cancers.* Nat Commun, 2016. **7**: p. 12222.
69. Serra, V., et al., *Clinical response to a lapatinib-based therapy for a Li-Fraumeni syndrome patient with a novel HER2V659E mutation.* Cancer Discov, 2013. **3**(11): p. 1238-44.
70. Lefebvre, C., et al., *Mutational Profile of Metastatic Breast Cancers: A Retrospective Analysis.* PLoS Med, 2016. **13**(12): p. e1002201.
71. Hyman, D.M., et al., *HER kinase inhibition in patients with HER2- and HER3-mutant cancers.* Nature, 2018. **554**(7691): p. 189-194.
72. Abaan, O.D., et al., *The exomes of the NCI-60 panel: a genomic resource for cancer biology and systems pharmacology.* Cancer Res, 2013. **73**(14): p. 4372-82.
73. Wu, G., et al., *The genomic landscape of diffuse intrinsic pontine glioma and pediatric non-brainstem high-grade glioma.* Nat Genet, 2014. **46**(5): p. 444-450.
74. Le Gallo, M., et al., *Somatic mutation profiles of clear cell endometrial tumors revealed by whole exome and targeted gene sequencing.* Cancer, 2017. **123**(17): p. 3261-3268.
75. Dulak, A.M., et al., *Exome and whole-genome sequencing of esophageal adenocarcinoma identifies recurrent driver events and mutational complexity.* Nat Genet, 2013. **45**(5): p. 478-86.
76. Papaemmanuil, E., et al., *Clinical and biological implications of driver mutations in myelodysplastic syndromes.* Blood, 2013. **122**(22): p. 3616-27; quiz 3699.
77. Mareschal, S., et al., *Whole exome sequencing of relapsed/refractory patients expands the repertoire of somatic mutations in diffuse large B-cell lymphoma.* Genes Chromosomes Cancer, 2016. **55**(3): p. 251-67.
78. Gunawardana, J., et al., *Recurrent somatic mutations of PTPN1 in primary mediastinal B cell lymphoma and Hodgkin lymphoma.* Nat Genet, 2014. **46**(4): p. 329-35.
79. Leich, E., et al., *Multiple myeloma is affected by multiple and heterogeneous somatic mutations in adhesion- and receptor tyrosine kinase signaling molecules.* Blood Cancer J, 2013. **3**: p. e102.
80. Bea, S., et al., *Landscape of somatic mutations and clonal evolution in mantle cell lymphoma.* Proc Natl Acad Sci U S A, 2013. **110**(45): p. 18250-5.
81. Landau, D.A., et al., *Evolution and impact of subclonal mutations in chronic lymphocytic leukemia.* Cell, 2013. **152**(4): p. 714-26.
82. Zhang, J., et al., *Deregulation of DUX4 and ERG in acute lymphoblastic leukemia.* Nat Genet, 2016. **48**(12): p. 1481-1489.
83. Gadd, S., et al., *A Children's Oncology Group and TARGET initiative exploring the genetic landscape of Wilms tumor.* Nat Genet, 2017. **49**(10): p. 1487-1494.
84. Lim, B., et al., *Genome-wide mutation profiles of colorectal tumors and associated liver metastases at the exome and transcriptome levels.* Oncotarget, 2015. **6**(26): p. 22179-90.
85. Seshagiri, S., et al., *Recurrent R-spondin fusions in colon cancer.* Nature, 2012. **488**(7413): p. 660-4.
86. Huang, J., et al., *Exome sequencing of hepatitis B virus-associated hepatocellular carcinoma.* Nat Genet, 2012. **44**(10): p. 1117-21.
87. Shi, J., et al., *Somatic Genomics and Clinical Features of Lung Adenocarcinoma: A Retrospective Study.* PLoS Med, 2016. **13**(12): p. e1002162.
88. Liu, P., et al., *Identification of somatic mutations in non-small cell lung carcinomas using whole-exome sequencing.* Carcinogenesis, 2012. **33**(7): p. 1270-6.
89. Biankin, A.V., et al., *Pancreatic cancer genomes reveal aberrations in axon guidance pathway genes.* Nature, 2012. **491**(7424): p. 399-405.

90. Yachida, S., et al., *Genomic Sequencing Identifies ELF3 as a Driver of Ampullary Carcinoma.* Cancer Cell, 2016. **29**(2): p. 229-40.
91. Grasso, C.S., et al., *The mutational landscape of lethal castration-resistant prostate cancer.* Nature, 2012. **487**(7406): p. 239-43.
92. Lindberg, J., et al., *The mitochondrial and autosomal mutation landscapes of prostate cancer.* Eur Urol, 2013. **63**(4): p. 702-8.
93. Lim, B., et al., *Genetic alterations and their clinical implications in gastric cancer peritoneal carcinomatosis revealed by whole-exome sequencing of malignant ascites.* Oncotarget, 2016. **7**(7): p. 8055-66.
94. Kang, H., et al., *Whole-Exome Sequencing of Salivary Gland Mucoepidermoid Carcinoma.* Clin Cancer Res, 2017. **23**(1): p. 283-288.
95. Wagle, N., et al., *MAP kinase pathway alterations in BRAF-mutant melanoma patients with acquired resistance to combined RAF/MEK inhibition.* Cancer Discov, 2014. **4**(1): p. 61-8.
96. Krauthammer, M., et al., *Exome sequencing identifies recurrent mutations in NF1 and RASopathy genes in sun-exposed melanomas.* Nat Genet, 2015. **47**(9): p. 996-1002.
97. Sanborn, J.Z., et al., *Phylogenetic analyses of melanoma reveal complex patterns of metastatic dissemination.* Proc Natl Acad Sci U S A, 2015. **112**(35): p. 10995-1000.
98. Hoogstraat, M., et al., *Detailed imaging and genetic analysis reveal a secondary BRAF(L505H) resistance mutation and extensive intrapatient heterogeneity in metastatic BRAF mutant melanoma patients treated with vemurafenib.* Pigment Cell Melanoma Res, 2015. **28**(3): p. 318-23.
99. Durinck, S., et al., *Temporal dissection of tumorigenesis in primary cancers.* Cancer Discov, 2011. **1**(2): p. 137-43.
100. Harms, P.W., et al., *The Distinctive Mutational Spectra of Polyomavirus-Negative Merkel Cell Carcinoma.* Cancer Res, 2015. **75**(18): p. 3720-3727.
101. Shern, J.F., et al., *Comprehensive genomic analysis of rhabdomyosarcoma reveals a landscape of alterations affecting a common genetic axis in fusion-positive and fusion-negative tumors.* Cancer Discov, 2014. **4**(2): p. 216-31.
102. Kim, T.M., et al., *The mutational burdens and evolutionary ages of early gastric cancers are comparable to those of advanced gastric cancers.* J Pathol, 2014. **234**(3): p. 365-74.
103. Pozdeyev, N., et al., *Genetic Analysis of 779 Advanced Differentiated and Anaplastic Thyroid Cancers.* Clin Cancer Res, 2018. **24**(13): p. 3059-3068.
104. Hedberg, M.L., et al., *Genetic landscape of metastatic and recurrent head and neck squamous cell carcinoma.* J Clin Invest, 2016. **126**(1): p. 169-80.
105. Hayes, T.F., et al., *Integrative genomic and functional analysis of human oral squamous cell carcinoma cell lines reveals synergistic effects of FAT1 and CASP8 inactivation.* Cancer Lett, 2016. **383**(1): p. 106-114.
106. Hurst, C.D., et al., *Genomic Subtypes of Non-invasive Bladder Cancer with Distinct Metabolic Profile and Female Gender Bias in KDM6A Mutation Frequency.* Cancer Cell, 2017. **32**(5): p. 701-715 e7.
107. Parker, M., et al., *C11orf95-RELA fusions drive oncogenic NF-kappaB signalling in ependymoma.* Nature, 2014. **506**(7489): p. 451-5.
108. Berger, M.F., et al., *The genomic complexity of primary human prostate cancer.* Nature, 2011. **470**(7333): p. 214-20.
109. Gonzalez-Vela, M.D.C., et al., *Shared Oncogenic Pathways Implicated in Both Virus-Positive and UV-Induced Merkel Cell Carcinomas.* J Invest Dermatol, 2017. **137**(1): p. 197-206.
110. Kakiuchi, M., et al., *Recurrent gain-of-function mutations of RHOA in diffuse-type gastric carcinoma.* Nat Genet, 2014. **46**(6): p. 583-7.

111. Molenaar, J.J., et al., *Sequencing of neuroblastoma identifies chromothripsis and defects in neuritogenesis genes.* Nature, 2012. **483**(7391): p. 589-93.
112. Ellis, M.J., et al., *Whole-genome analysis informs breast cancer response to aromatase inhibition.* Nature, 2012. **486**(7403): p. 353-60.
113. Nik-Zainal, S., et al., *Mutational processes molding the genomes of 21 breast cancers.* Cell, 2012. **149**(5): p. 979-93.
114. Shankar, G.M., et al., *BRAF alteration status and the histone H3F3A gene K27M mutation segregate spinal cord astrocytoma histology.* Acta Neuropathol, 2016. **131**(1): p. 147-50.
115. Agrawal, N., et al., *Comparative genomic analysis of esophageal adenocarcinoma and squamous cell carcinoma.* Cancer Discov, 2012. **2**(10): p. 899-905.
116. Chang, J., et al., *Genomic analysis of oesophageal squamous-cell carcinoma identifies alcohol drinking-related mutation signature and genomic alterations.* Nat Commun, 2017. **8**: p. 15290.
117. Lin, D.C., et al., *Genomic and molecular characterization of esophageal squamous cell carcinoma.* Nat Genet, 2014. **46**(5): p. 467-73.
118. Li, B., et al., *Negative feedback-defective PRPS1 mutants drive thiopurine resistance in relapsed childhood ALL.* Nat Med, 2015. **21**(6): p. 563-71.
119. Varela, I., et al., *Exome sequencing identifies frequent mutation of the SWI/SNF complex gene PBRM1 in renal carcinoma.* Nature, 2011. **469**(7331): p. 539-42.
120. Dalgliesh, G.L., et al., *Systematic sequencing of renal carcinoma reveals inactivation of histone modifying genes.* Nature, 2010. **463**(7279): p. 360-3.
121. Yu, J., et al., *Novel recurrently mutated genes and a prognostic mutation signature in colorectal cancer.* Gut, 2015. **64**(4): p. 636-45.
122. Kan, Z., et al., *Diverse somatic mutation patterns and pathway alterations in human cancers.* Nature, 2010. **466**(7308): p. 869-73.
123. Peifer, M., et al., *Integrative genome analyses identify key somatic driver mutations of small-cell lung cancer.* Nat Genet, 2012. **44**(10): p. 1104-10.
124. Wang, L., et al., *Whole-exome sequencing of human pancreatic cancers and characterization of genomic instability caused by MLH1 haploinsufficiency and complete deficiency.* Genome Res, 2012. **22**(2): p. 208-19.
125. Witkiewicz, A.K., et al., *Whole-exome sequencing of pancreatic cancer defines genetic diversity and therapeutic targets.* Nat Commun, 2015. **6**: p. 6744.
126. Barbieri, C.E., et al., *Exome sequencing identifies recurrent SPOP, FOXA1 and MED12 mutations in prostate cancer.* Nat Genet, 2012. **44**(6): p. 685-9.
127. Bova, G.S., et al., *Integrated clinical, whole-genome, and transcriptome analysis of multisampled lethal metastatic prostate cancer.* Cold Spring Harb Mol Case Stud, 2016. **2**(3): p. a000752.
128. Dalin, M.G., et al., *Comprehensive Molecular Characterization of Salivary Duct Carcinoma Reveals Actionable Targets and Similarity to Apocrine Breast Cancer.* Clin Cancer Res, 2016. **22**(18): p. 4623-33.
129. Li, Y.Y., et al., *Genomic analysis of metastatic cutaneous squamous cell carcinoma.* Clin Cancer Res, 2015. **21**(6): p. 1447-56.
130. Berger, M.F., et al., *Melanoma genome sequencing reveals frequent PREX2 mutations.* Nature, 2012. **485**(7399): p. 502-6.
131. Zhang, L., et al., *Whole-exome sequencing identifies a somatic missense mutation of NBN in clear cell sarcoma of the salivary gland.* Oncol Rep, 2016. **35**(6): p. 3349-56.
132. Shankar, G.M., et al., *Sporadic hemangioblastomas are characterized by cryptic VHL inactivation.* Acta Neuropathol Commun, 2014. **2**: p. 167.

133. Kohsaka, S., et al., *A recurrent neomorphic mutation in MYOD1 defines a clinically aggressive subset of embryonal rhabdomyosarcoma associated with PI3K-AKT pathway mutations.* Nat Genet, 2014. **46**(6): p. 595-600.
134. Ibrahimpasic, T., et al., *Genomic Alterations in Fatal Forms of Non-Anaplastic Thyroid Cancer: Identification of MED12 and RBM10 as Novel Thyroid Cancer Genes Associated with Tumor Virulence.* Clin Cancer Res, 2017. **23**(19): p. 5970-5980.
135. Pickering, C.R., et al., *Integrative genomic characterization of oral squamous cell carcinoma identifies frequent somatic drivers.* Cancer Discov, 2013. **3**(7): p. 770-81.
136. Pinto, E.M., et al., *Genomic landscape of paediatric adrenocortical tumours.* Nat Commun, 2015. **6**: p. 6302.
137. Martin, M., et al., *Exome sequencing identifies recurrent somatic mutations in EIF1AX and SF3B1 in uveal melanoma with disomy 3.* Nat Genet, 2013. **45**(8): p. 933-6.
138. Walter, M.J., et al., *Clonal architecture of secondary acute myeloid leukemia.* N Engl J Med, 2012. **366**(12): p. 1090-8.
139. Sanz-Pamplona, R., et al., *Exome Sequencing Reveals AMER1 as a Frequently Mutated Gene in Colorectal Cancer.* Clin Cancer Res, 2015. **21**(20): p. 4709-18.
140. Cho, J., et al., *Colon cancer-derived oncogenic EGFR G724S mutant identified by whole genome sequence analysis is dependent on asymmetric dimerization and sensitive to cetuximab.* Mol Cancer, 2014. **13**: p. 141.
141. Pleasance, E.D., et al., *A small-cell lung cancer genome with complex signatures of tobacco exposure.* Nature, 2010. **463**(7278): p. 184-90.
142. Balbas-Martinez, C., et al., *Recurrent inactivation of STAG2 in bladder cancer is not associated with aneuploidy.* Nat Genet, 2013. **45**(12): p. 1464-9.

Supplementary Figure S1. Sequence alignment of MRE11, RAD50 and NBN.

A. MRE11

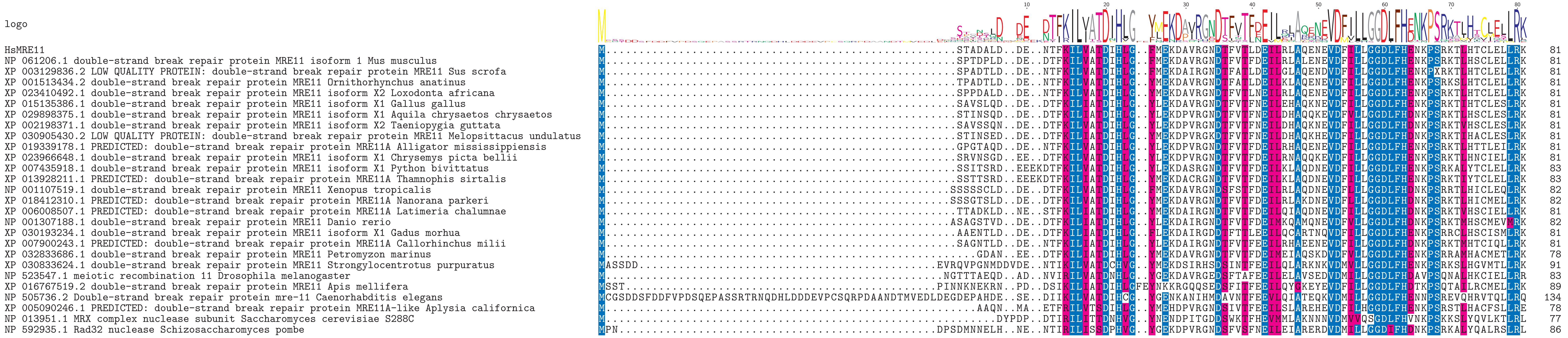

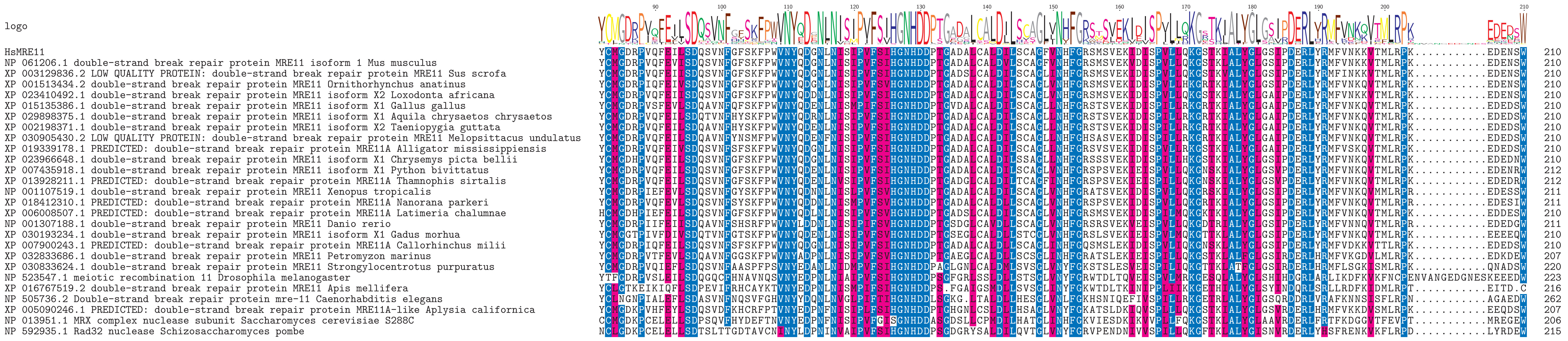

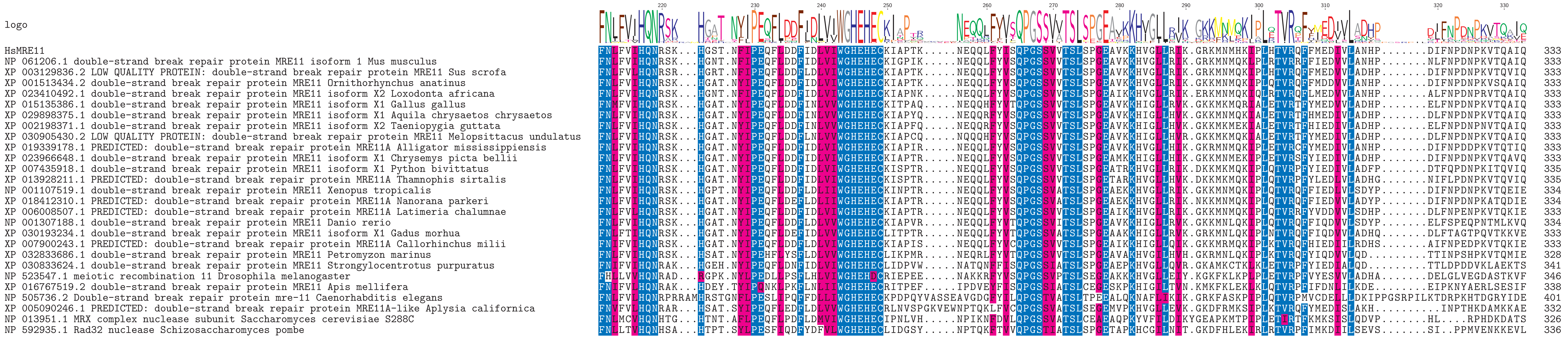

logo

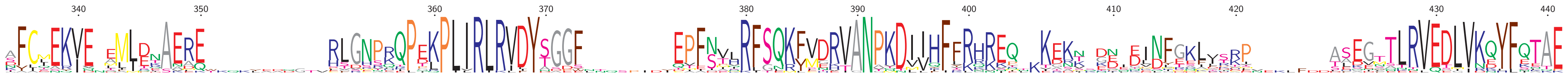


```
HsMRE11  SFCLEKIE.EMLENAERE...........RLGNSHQPEKPLVRLRVDYSGGF........EPFSVLRFSQKFVDRVANPKDIIHFFRHREQ..KEKT.GE.EINFGKLITKP........SEGTTLRVEDLVKQYFQTAE  441
NP 061206.1 double-strand break repair protein MRE11 isoform 1 Mus musculus  SFCLEKIE.EMLDSAERE...........RLGNPQQPGKPLIRLRVDYSGGF........EPFNVLRFSQKFVDRVANPKDIIHFFRHREQ..KGKT.GE.EINFGMLITKP.......ASEGATLRVEDLVKQYFQTAE  442
XP 003129836.2 LOW QUALITY PROTEIN: double-strand break repair protein MRE11 Sus scrofa  SFCLEKIE.EILENAERE...........RLGNSRQPEKPLIRLRVDYSGGF........EPFSVLRFSQKFVDRVANPKDIIHFFRHKEQ..KENM.GE.EINLGKFIPKP........SEGTTLRVEDLVKQYFQTAE  441
XP 001513434.2 double-strand break repair protein MRE11 Ornithorhynchus anatinus  NFCLEKVE.EMLDKAERE...........RLGNPQRPEKPLIRLRVDYSGGF........EPFSVLRFSQKFVDRIANPKDIIHFFRHREQ..KEKT.DE.DINFGKFIRKP.......ASEGMTLRVEDLVKQYFQTAE  442
XP 023410492.1 double-strand break repair protein MRE11 isoform X2 Loxodonta africana  SFCLEKIE.EMLENAERE...........RLGNPQQPEKPLIRLRVDYSGGF........EPFSVLRFSQKFVDRVANPKDIIHFFRHREP..KEKT.GE.EINFGKLITRP.......SLEGTTLRVEDLVKQYFQTAE  442
XP 015135386.1 double-strand break repair protein MRE11 isoform X1 Gallus gallus  AFCMEKVE.MMLDNAERE...........RLGNPRQPQKPLIRLRVDYTGGF........EPFIVHRFSQKYMDRVANPKDIIHFFRHREQ..KEKN.DN.DINFGKLLSRP.......ASEEVTLRVEDLVKQYFQTAE  442
XP 029898375.1 double-strand break repair protein MRE11 isoform X1 Aquila chrysaetos chrysaetos  AFCMEKVE.LMLDNAERQ...........RLGNPRQPEKPLIRLRVDYAGGF........EPFNVHRFSQKYMDRVANPKDIIYFFRHREQ..KEKN.DN.DLNFGKLVSRP.......TAEGMTLRVEDLVKQYFQTAE  442
XP 002198371.1 double-strand break repair protein MRE11 isoform X2 Taeniopygia guttata  AFCMEKVE.LMLDTAERE...........RLGNPRQPEKPIIRLRVDYAGGF........EPFSVHRFSQKYMHRVANPKDIVHFFRHREQ..KEKK.DI.DLNFGKLVSRP........AEGTTLRVEDLVKQYFQTAE  441
XP 030905430.2 LOW QUALITY PROTEIN: double-strand break repair protein MRE11 Melopsittacus undulatus  AFCMEKVE.MMLDNAERE...........RLSNPRQPGKPLIRLRVDYAGGF........EPFSVHRFSQKYMDRVANPKDIVHFFRRREQ..KEKN.DN.DLDFGNLVSRP.......ASDEMTLRVEDLVKQYFQTAE  442
XP 019339178.1 PREDICTED: double-strand break repair protein MRE11A Alligator mississippiensis  AFCMEKVD.AMLDKSEME...........RLGSPRQPEKPLIRLRVDYTGGF........EPFNVHRFSQKFMDRVANPKDIVHFFRRREQ..KEKN.EN.EINFGKLLSRP.......ASEGTTLRVEDLVKQYFQTAE  442
XP 023966648.1 double-strand break repair protein MRE11 isoform X1 Chrysemys picta bellii  AFCIEKVE.TLLDNAERE...........RLGNPRQPEKPLIRLRVDYSGGF........EPFSIHRFSQKFMERTANPKDIIHFFRHREQ..KEKN.ED.EINFGKLIRRP.......ASEGTTLRVEDLVKKYFQTAE  442
XP 007435918.1 double-strand break repair protein MRE11 isoform X1 Python bivittatus  DFCMEKVE.MMLENSERE...........HLRNPKQPEKPLIRLRVDYSGGF........EPFSIVRFSQKYLDRVANPKDIIHFFRHREQ..KEKN.ED.EVSFRKLDHKP.......TAEGTTLRVEDLVKQYFQTAE  444
XP 013928211.1 PREDICTED: double-strand break repair protein MRE11A Thamnophis sirtalis  DFCMEKVE.LMLENAERQ...........SFGNPKQPEKPLIRLRVDYSGGF........EPFSMVRFSQKYMDRVANPKDIIHFFRHREQ..KEKN.ED.DVNFRKLVPKP.......AAEGTTLRVEDLVKQYFQTAE  444
NP 001107519.1 double-strand break repair protein MRE11 Xenopus tropicalis  SFCIEKVE.AMLDTAERE...........RLGNARQPDKPLIRLRVDYTGGF........EPFNTLRFSQKFVDRTANPKDIIHFFRHKEQ..KDKK.DNITINFGKLDSKP.......LFEGSTLRVEDLVKEYFKTAE  444
XP 018412310.1 PREDICTED: double-strand break repair protein MRE11A Nanorana parkeri  NFCIEKIE.SMLDIAERE...........RLGNTRQPDKPLIRLRVDYTGGF........EPFNTLRFSQKFVDKTANPKDIIHFFRHKEQ..KDKK.DNIAINFEKLESRP.......SSEVATLRVEDLVKQYFQTAE  444
XP 006008507.1 PREDICTED: double-strand break repair protein MRE11A Latimeria chalumnae  GFCTERVE.TMLETAERE...........CLDNSKQPDKPLIRLRVDYSGGF........EPFSTLRFSQKFVNRVANPKDIIHFFRHREQ..KDRK.GDEQINFDKFVTCP.......AADGMTLRVEDLVKQYFETTE  443
NP 001307188.1 double-strand break repair protein MRE11 Danio rerio  AFCQEKVE.EMLEEAERE...........RLGNPQIPEKPLIRLRVDYSGGF........EVFNTMRFSQKFVDKVANPKDILHFVRHRET..KGNI.KDEDVDFEALFSRP.......TSEVLQLRVEDLVKEYFQTAE  444
XP 030193234.1 double-strand break repair protein MRE11 isoform X1 Gadus morhua  DFCQAKVV.EMLDQAERE...........RLGCPLTPEKPLIRIRVDYSGGF........EAFNTSRFSQKFVDRVANPKDVIHFLRRREN..KEAVKDEIDIDYSRLVRTT.......ALEG..LRVEDLVKQYFEAAE  442
XP 007900243.1 PREDICTED: double-strand break repair protein MRE11A Callorhinchus milii  AFCAEKVE.ALLDLAERE...........HLGNPGQPEKPLIRLRVDYS.GF........EPFGMLRFSQRFVERVANPKDLIHFLRQKEQ..KDKK.DDQKINFEKCTIRS.......ASEGFTLRVEDLVKQYFETAE  442
XP 032833686.1 double-strand break repair protein MRE11 Petromyzon marinus  NYCSEKVE.DMLEQAAAE...........RSGHSKQPDRPLIRLRIDYSGGF........EPFNTLRFSQKFVEHVGNPKDIIHFFRQREM..KART.GD.EMDFGQAFSHV.......AHGENTLRVEDLVKEYFKSAE  437
XP 030833624.1 double-strand break repair protein MRE11 Strongylocentrotus purpuratus  AFCTEKVE.ALIQRAEDE...........HTGNRKQPTLPLIRLRVDYSGGF........DTINVNRFGQKFVGRVANTKDLVRFTKRRIQLKKEDT.KP.GEDLGEFLKPE.......ALE..TERVEDLVRKYFSETE  450
NP 523547.1 meiotic recombination 11 Drosophila melanogaster  KFAQERVE.AMIERAVAQ...........HTGHPKQPTLPLIRLRLLYTDES........CMFNAIRFGEMLSTRVANVQDVVQFSKVVKRTKTEAV....NLDKEALRRAL........EADNATRVEELVDRYFEEAK  454
XP 016767519.2 double-strand break repair protein MRE11 Apis mellifera  KFIDNYIQNELMPKAALQ...........LSGHPKQPILPLLRLRIFYNSDE........EIFDEIKLTQKYCDEVANPMDIVIFRKQKNINKKSNSSNSLEDDFEDIAQVFN...FDDDEKNWNKTVQGGIKNHFSLKE  456
NP 505736.2 Double-strand break repair protein mre-11 Caenorhabditis elegans  IAIEAKIN.EMITTAKAK...........R..RPRQPELPLIRLKVIYDGDWLN.....ITPANAKRIGLRYENVVANAVDMVFIKKNNKP.KEGKLQTENEKNITEMADEM........GQVSATNLQTIINDYFINQP  513
XP 005090246.1 PREDICTED: double-strand break repair protein MRE11A-like Aplysia californica  DFCVEKIE.QFISKAEDE...........HSGSRRQPTQPLIRLRIDYT.GF........QPFSSQRLGQKFVDHVANPKTMIHFLRGRVE.KIASV.KQ.DPQMMEKIKTI.......TLD..SNRVEDMVRDYFVKAE  439
NP 013951.1 MRX complex nuclease subunit Saccharomyces cerevisiae S288C  KYLIEQVE.EMIRDANEETKQKLADDG..EGDMVAELPKPLIRLRVDYSAPSNTQSPIDYQVENPRRFSNRFVGRVANGNNVVQFYKKRSP..VTRS.KKSGINGTSISDRDVEKLF..SESGGELEVQTLVNDLLN...  455
NP 592935.1 Rad32 nuclease Schizosaccharomyces pombe  TYLISKVE.EAITEANAQW...YEAQGTVPVVENEKPPLPLIRLRVDYTGGY........QTENPQRFSNRFVGRVANATDVVQFYLKKKY..TRSK.RN.DGLYTSAVEDI.........KINSLRVESLVNEYLK...  448
```

logo

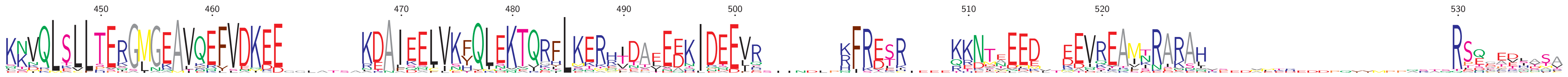


```
HsMRE11  KNVQLSLLTERGMGEAVQEFVDKEE.......KDAIEELVKYQLEKTQRFLKERHIDALEDKIDEEVR.......RFRETR....QKNTNEED..DEVREAMTRARAL......................RSQSEESASA  539
NP 061206.1 double-strand break repair protein MRE11 isoform 1 Mus musculus  KNVQLSLLTERGMGEAVQEFVDKEE.......KDAIEELVKYQLEKTQRFLKERHIDALEDKIDEEVR.......RFRESR....QRNTNEED..DEVREAMSRARAL......................RSQSETSTSA  540
XP 003129836.2 LOW QUALITY PROTEIN: double-strand break repair protein MRE11 Sus scrofa  KNVQLSLLTERGMGKAVQEFVDKEE.......KDAIQELVKYQLEKTQRFLKERHIDALEDKIDEEVR.......RFRESR....QKNTNEED..DEVQEAMNRARAL......................RSQEEDAASA  539
XP 001513434.2 double-strand break repair protein MRE11 Ornithorhynchus anatinus  KNVQLSLLTERGMGEAVQEFVDKEE.......KDAIEELVKYQLEKTQRFLKERHIDAVEDKIDEEVR.......RFRETR....RENSAEEE..DEVREAMNRARAH......................RSQFEDSASV  540
XP 023410492.1 double-strand break repair protein MRE11 isoform X2 Loxodonta africana  KNVQLSLLTERGMGEAVQEFVDKEE.......KDAIEELVKYQLEKTQRFLKERHIDAVEDKIDEEVR.......RFRESR....QRNTDEED..DEVREAMSRARAH......................RSQLEDTASA  540
XP 015135386.1 double-strand break repair protein MRE11 isoform X1 Gallus gallus  KKVQLSLLTERGMGEAVQEFVDKEE.......KDAIEELVKFQLEKTQRFLKERHIDAEEEKIDEEVR.......KFRESR....RKNTEEED..EEVREAMTRARAH......................RSEGVVLDSA  540
XP 029898375.1 double-strand break repair protein MRE11 isoform X1 Aquila chrysaetos chrysaetos  KKVQLSLLTERGMGEAVQEFVDKEE.......KDAIEELVKFQLEKTQRFLKERCTDAEEEKIDEEVR.......KFRESR....KKNTEEED..EEVREAMTRARAH......................RSEDAVLVAA  540
XP 002198371.1 double-strand break repair protein MRE11 isoform X2 Taeniopygia guttata  KKVQLSLLTERGMGEAVQEFVDKEE.......KEAIEELVKFQLEKTQRFLKERRTDAVEEKIDEEVR.......KFRESR....KKNTEEED..KEVREAMIRARAH......................RSEDEVLVAA  539
XP 030905430.2 LOW QUALITY PROTEIN: double-strand break repair protein MRE11 Melopsittacus undulatus  KKVQLSLLTERGMGEAVQEFVDKEE.......KDAIEELVKFQLEKTQRFLKERRTAAEEEKIDEEVR.......KFRESR....KKNAEEED..EEVREAMTRARAHRSEDAMTR..........ARAQRSEDAVLVTA  552
XP 019339178.1 PREDICTED: double-strand break repair protein MRE11A Alligator mississippiensis  KKVQLSLLTERGMGEAVQEFVDKEE.......KDAIEELVKFQLEKTQRFLKERRTDAEEEKIDEEVR.......KFRDTR....KKNTEEED..EEVREAMTRARAH......................RSQETVHTTF  540
XP 023966648.1 double-strand break repair protein MRE11 isoform X1 Chrysemys picta bellii  KKVQLSLLTERGMGEAVQEFVDKEE.......KDAIEELVKFQLEKTQRFLKERHTDAEEAKIDEEVR.......KFRETR....KENIEEEE..EEVREAMTRARAH......................RSHEAALASC  540
XP 007435918.1 double-strand break repair protein MRE11 isoform X1 Python bivittatus  KKVQLSLLTERGMGEAVQEFVDKEE.......KDAIEELVKFQLEKTQRFLKEKHIDAEEAKIDEEVQ.......HFRESR....KKNMVEED..EEVREAINRAKA.......................RSQGTDHASV  541
XP 013928211.1 PREDICTED: double-strand break repair protein MRE11A Thamnophis sirtalis  KKVQLSLLTERGMGEAVQEFVDKEE.......KDAIEELVKFQLEKTQRFLKERHTDAEEAKIDEEVQ.......RFRESK....KKNTVEED..EEVREAINRAKA.......................RSQATGHTST  541
NP 001107519.1 double-strand break repair protein MRE11 Xenopus tropicalis  KNVQLSLLTERGMGEAVQEFVDKEE.......KDALEELVKFQLEKTQRFLKERQIDAEEDKIDEEVR.......KFRETR....KTNTNEEE..EEVREAMIRARAH......................RSQTADIDM.  541
XP 018412310.1 PREDICTED: double-strand break repair protein MRE11A Nanorana parkeri  KNVQLSLLTERGMGEAVQEFVDKEE.......KDAIEELVKFQLEKTQRFLKERKFDV.EEKIDE..................................EAINRARAH......................RSQAEDINMS  520
XP 006008507.1 PREDICTED: double-strand break repair protein MRE11A Latimeria chalumnae  KNVQLSLLTERGMGEAVQEFVDKEE.......KDAIEELVKFQLEKTQKFLKERHTDALEEKIDEEVR.......HFRESR....QKNTAEED..EEVREAMNRARAN......................RSEEERVCFV  541
NP 001307188.1 double-strand break repair protein MRE11 Danio rerio  KNVQLSLLTEQGMGKAVQEFVDKEE.......RDAIEELINYQLEKTQRYLRERRVDATEEKIDEEIR.......QFRDS.....KRNTAEED..EEVQEAIIRAKAH......................RDRGETAR..  539
XP 030193234.1 double-strand break repair protein MRE11 isoform X1 Gadus morhua  QKVQLSLLTESGMGKAIQEFVDKDE.......KEAIEELISYQLEKTQRHLQGRGVTT.EQDIDAEIQ.......RFRDA.....KKNTTEEE..RENREALDRARAL......................RLDRGEAPQ.  537
XP 007900243.1 PREDICTED: double-strand break repair protein MRE11A Callorhinchus milii  KSIQLSLLTERGMGEAVQEFVDKEE.......KDAIDELVKFQLEKTQRFLKERRIEGIEDKIDEEVR.......HFRDSR....KKNEADED..ADVREAMKRARAH......................RTEGKEISTR  540
XP 032833686.1 double-strand break repair protein MRE11 Petromyzon marinus  KNVQLALLTERGMGAAVQEFVDKEE.......KEAISELVKVQLTKTQEHLKKTEVSS.VDTIDEEVR.......HFRESC....KKNM.QED..EDIKEAVSRSRAI......................RPTGETSDH.  532
XP 030833624.1 double-strand break repair protein MRE11 Strongylocentrotus purpuratus  ETKQLELLSEKGLGQAVREYVDKEE.......KEAIQALVKHQLSQTQGQLKNRKTT..EEHIDDELH.......RFKVER.....REMTEADVTQEVKAAITEDRSQ......................RPDAYSQ...  544
NP 523547.1 meiotic recombination 11 Drosophila melanogaster  SNKPLKLFHSKALAEMTYRLLEQRD.......ADAAENIVKFYKEKAVDHLMEAMPN..DENIDEELE.......RFRALH....KHGDLLKML.DTQGLKVKKEEK.................................  540
XP 016767519.2 double-strand break repair protein MRE11 Apis mellifera  NKDKLTVLTVNGLNEALNRFVNMGD.......LDAFKGIVNHQMNKTITLLETCEVDT.SESIRNEIK.......NIRDKRIEEEKEDTMVDK..MEIQTAFNNIKN.......................QAECFEI...  553
NP 505736.2 Double-strand break repair protein mre-11 Caenorhabditis elegans  LVDQMTVLKPIGIGRALEQYSAIEEGGLATSANRNFDSCLMHQIDVVRKTLKKMPLPPIND..PSDLD.......KFRDLI....TKDLYDMK..KADSERFKNPVADVE..MEEDEDDPQYYMPPQSTSRTNYASGS..  634
XP 005090246.1 PREDICTED: double-strand break repair protein MRE11A-like Aplysia californica  GDNQLKLLSEKGLGEAVKEYVDKEE.......REAISQLVKYQLQKTQGYLMSRNSN..EDQIQTDLG.......KFKEER....KKKVTEED..EDVKVALERAKSI......................RESQPENG..  533
NP 013951.1 MRX complex nuclease subunit Saccharomyces cerevisiae S288C  .KMQLSLLPEVGLNEAVKKFVDKDE.......KTALKEFISHEISNEVGILST..........NEEFL.................RTDDAEEM..KALIKQVKRANSV......................RPTPPKE...  533
NP 592935.1 Rad32 nuclease Schizosaccharomyces pombe  .TNRLECLPEDSLGEAVVNFVEKDD.......RDAIKECVETQLNKQINLLVKKRVT..EENLEQEISSIINDLPKISTTK....RKDYEELP..EEVSETSINIAEHTP..VLKH..............TSSLLDH...  553
```

logo

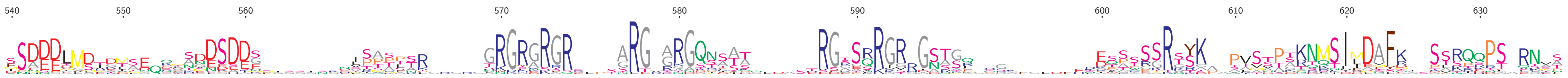


```
HsMRE11  FSADDLMSIDLAE.QMANDSDDS........ISAATNK.....GRGRGRGR.....RG.GRGQNSAS......RGGSQRGR.ADTGL...........ETSTRSRNSK..TAVSASRNMSIIDAFK..STRQQPS.RNVT  636
NP 061206.1 double-strand break repair protein MRE11 isoform 1 Mus musculus  FSAEDL.SFDTSE.QTANDSDDS........LSAVPSR.....GRGRGRGR.....RG.ARGQSSAP......RGGSQRGR.D.TGL...........EITTRGRSSK..ATSSTSRNMSIIDAFR..STRQQPS.RNVA  635
XP 003129836.2 LOW QUALITY PROTEIN: double-strand break repair protein MRE11 Sus scrofa  FSAEDLMSIDMAE.QTANDSDDS........ITAAASR.....GRGRGRGR.....RG.GRGQSLVS......RGASQRGR.G.TGL...........ETSTPSRSSK..ATMSTSRNMTIIDAFK..SARKQPS.QNAA  635
XP 001513434.2 double-strand break repair protein MRE11 Ornithorhynchus anatinus  FSADDLMSIELAE.QMANDSDDS........ISAMSTK.....GRGRGRGSR...ARG.ARGQSSAA......RGGARRGR.GSSCQ...........ESPNRSRSSKA.SVSTPTKNMSIMDAFK..STRQQPT.RNVA  640
XP 023410492.1 double-strand break repair protein MRE11 isoform X2 Loxodonta africana  VSADDLMSVDLAE.EMADDSDDS........NSAAANR.....GRGRGRGR.....RG.GRGQNSAS......RGEPQRGR.G.TGM...........ATASRSRSSKP.TTSASSRNMSILDVFK..PARQQPS.GNAA  637
XP 015135386.1 double-strand break repair protein MRE11 isoform X1 Gallus gallus  SSDEGLMDTGM...KASGDSDDD........IPTTLSR.....GRG..RGR....ARG.ARGQNSAA......RGSSRRGR.GNTSQ...........GSSTSSRTYK....SVPDKNLSIMDAFR..SLKPEPS.QS.T  631
XP 029898375.1 double-strand break repair protein MRE11 isoform X1 Aquila chrysaetos chrysaetos  SSDEELMDTGM...KDTGDSDDG........LPATLRR.....GRGWARGR....ARG.ARGQNSTA......RGSSRRGR.GSTSQ...........ESATSSRTYK..PVSVPAKNMSIMDAFR..SLKPEPS.LN.S  635
XP 002198371.1 double-strand break repair protein MRE11 isoform X2 Taeniopygia guttata  SSDEDSMDIEM...TGTSNPSDG........LSSTLSR.....GRGRARGR....ARG.ARGQNSTA......RGSSRRGR.GATSQ...........ESASGSRSYK..PVSGPGKNMSIMDAFR..SSKTDRS.SN.S  634
XP 030905430.2 LOW QUALITY PROTEIN: double-strand break repair protein MRE11 Melopsittacus undulatus  SSDEEAMDTGM...KGSGDSDDG........FPATLTR.....GRGRARGR....ARG.ARGQNPTA......RGSSQRRR.GSSGR...........ESGPSSRTYK..PLSVPAKNASIMDAFK..SLKPDPL..N.S  646
XP 019339178.1 PREDICTED: double-strand break repair protein MRE11A Alligator mississippiensis  SSDDELMDTRTMEQKDSDDSDDGF......SISATTSR.....GRGRGRTR....ARG.TRGQNAAT......RGVSRRGR.GNTSQ...........ESSSSSRTYK..PIATPTKNMSIFDAFK..SSRQQPS.RNSS  641
XP 023966648.1 double-strand break repair protein MRE11 isoform X1 Chrysemys picta bellii  SSDDDLMDMGMSEQKNNDDSDAG........TSVSFSR.....GRGRGRGQ....ARG.GRGQISTS......RGASRRGR.GNTTQ...........EQSNSSRSYK..PVSTPTKNMSIIDAFK..AVRQQPS.RNSS  639
XP 007435918.1 double-strand break repair protein MRE11 isoform X1 Python bivittatus  MSDEDLMDVATSDQRANDESEDS........LAPS.........RGRRRGR....ARG.RGGQNISA......RGASQRGRGGSTGR...........DQTSSGRTYK..PVTTPTKNMLIWDAFK..SSRQQPS.RKMP  637
XP 013928211.1 PREDICTED: double-strand break repair protein MRE11A Thamnophis sirtalis  MSDDDLVDEAPLDQRVNDESDDS........LPPS.........WGRKRGR....ARG..RGQNPST......RGASQRGRRGSASR...........DQAGSCRTYK..PASTPTKTMSILDAFK..SSRQQPS.RKTS  636
NP 001107519.1 double-strand break repair protein MRE11 Xenopus tropicalis  .SDDDELMRKVSG.PLSDDEDIG........ASMP........ARGRGRGR....GRG.GRGQTTTT......RGTSRRGR.GSAGA...........DQPSSGRASK.....AAGKNMSILDAFK..PSSRQPTARNMP  633
XP 018412310.1 PREDICTED: double-strand break repair protein MRE11A Nanorana parkeri  DNDDDGMGRKTSN.PLNDDSDDN........IPTP........TRGKGRGR....ARG.GRGQGAST......RGTSRRGR.GSS.............DPPSSGRASK.....APAKNMSIFDAFKASSSRQSTS.RNVP  612
XP 006008507.1 PREDICTED: double-strand break repair protein MRE11A Latimeria chalumnae  ISDDEM...DISE.PRGEDSDSS........VVPS...........RGRGR....ARG.GRGQSAAA......RGTSRRGR.GNASQGQRA.....ESKASSSSRSYKA.PVVPPAKNMSIMDAFK..SSSRHPS.RTGT  637
NP 001307188.1 double-strand break repair protein MRE11 Danio rerio  ..DDDLGLSDDPA.DIAMESDEE.......PSSAPP.......VRGRGRG.....SRG.GRDPCASK......RG...RAR.GAASR............SGRGGRGRG..SLSSPVKTKSIMDVFQ..DVSQRST.RGKT  629
XP 030193234.1 double-strand break repair protein MRE11 isoform X1 Gadus morhua  DPMEELG.......DAHMDSDEG........VTPPPAP.....TRGRGSR.....GRG.GRG................RGR.GAAAP...........EPKAAGRGRG..QRSSAGGSRSIMEAFD..RPSQRRP.RAGA  618
XP 007900243.1 PREDICTED: double-strand break repair protein MRE11A Callorhinchus milii  ISDDDD.DLTIT..SEKDEDADE........IPMAFT......NKGHARGR....GRG.SRGQSSST.....ERGGSRRGSRGNSRE.TGK.....ETRVPSGSRSYKP.PVVTTTKNKSIIDAFK..PSNQRPT.RTLN  643
XP 032833686.1 double-strand break repair protein MRE11 Petromyzon marinus  ..DDDD........QDDEERADE........VRTPT.......SRGRGKG.....SRGRARGQGSAG.....TRGRTLRGGRGSRGK.AGGFGLDFHNEPPPSARRKA..PAANVAPQSTIDAMFT..TSTQKPS.RSAS  631
XP 030833624.1 double-strand break repair protein MRE11 Strongylocentrotus purpuratus  ..............DYGVDSDEESIGGGNGDGPSRST.........RGRAR....GRG.AAGSTA........RGRGSRGGRGSRGG.RG.......RGQSSSSRGSA.......PTQQTLLMTSS..SSRTKPG.VLAF  630
NP 523547.1 meiotic recombination 11 Drosophila melanogaster  ..............SMSVLGSEV.......NVSTAPAT.....GRGAARGRG.TART.RAGATAAT......RGKGQ...................LDVTVSSTRTS.......GRQQTLLSSLMG.............  606
XP 016767519.2 double-strand break repair protein MRE11 Apis mellifera  ..DSDI........NLSDEDEKK.......NMIPK........SKRSERS.....TRG.RNGRNKTK......EFTAKKIS........S.......KIDGIEKRKTK.......KQDQNILENYM..............  620
NP 505736.2 Double-strand break repair protein mre-11 Caenorhabditis elegans  ..EDEV...........ANSDEEMGSSISRHSKQPT.......TRGRGRG.....ARG.AGASRETT......RGRSNKVV..................................STRQIDSDGFEI.............  695
XP 005090246.1 PREDICTED: double-strand break repair protein MRE11A-like Aplysia californica  ..ENET........GFSDDSDDE.......DGPSSSTRGRGRGGRGRGRGR....GRG.AKADTGADGLDASTSSRSSRGGRGSRGG.RG.............SRGSKAAVVMDTSRSRSVRDMFA..SASASNS.....  630
NP 013951.1 MRX complex nuclease subunit Saccharomyces cerevisiae S288C  ..NDET........NFAFN........................GNGLDSFRS..SNREVRTGSPDITQ...SHVDNESRITHISQAE.SS.......KPTSKPKRVRT.......ATKKKIPAFSD..STVISDA.ENEL  616
NP 592935.1 Rad32 nuclease Schizosaccharomyces pombe  ..HSPL........ATSSSEHEM.......EATPSPAL.....LKKTNKRRELPSSLT.KKNTRTPQ......RSKEVKKV.........PA.....RKLSQSTKKSD.......KNTQSTLLFYD.......PS.....  631
```

```
logo

HsMRE11                                                                          ..TKNYSEVIE.VDESDV.........EEDIFP.TTSKTDQRW......SSTSS................SKIMSQSQVSKGVDFESSED....DDD.DP....FMN.TSSLRRNRR........................ 708
NP 061206.1 double-strand break repair protein MRE11 isoform 1 Mus musculus     ..PKNYSETIE.VDDSD..........EEDIFP.TNSRADQRW......SGTTS................SKRMSQSQTAKGVDFESDED....DDD.DP....FMS.SSCPRRNRR........................ 706
XP 003129836.2 LOW QUALITY PROTEIN: double-strand break repair protein MRE11 Sus scrofa  ..PKNYSEVIE.VDESDT.........EEDIFP.TTSKTDQRWL.....STSSS................SKRASQSQVARGVDFESDED....DDD.DP....FMN.LSSLRRNRR........................ 708
XP 001513434.2 double-strand break repair protein MRE11 Ornithorhynchus anatinus  ..TKNYSEVIV.DDDSDM.........EDDLFP.ASSKVDQRL..........S................SKRGSQSQNTKGVDFESDE.....EDD.DP....FKN.ISSFGRNPR........................ 707
XP 023410492.1 double-strand break repair protein MRE11 isoform X2 Loxodonta africana  ..TKNYSEVIE.VEQSDE.........EDDIFP.TSSKTDQRR......PGVSS................SKRVSQVQIAKGVDFESDED....DDD.DP....FMN.INSLRRTRR........................ 709
XP 015135386.1 double-strand break repair protein MRE11 isoform X1 Gallus gallus  ..SKFFSEDII.DDEMDL.........EESPIS.LSSKTNQRS......SAMSS..............FSKRGSQSQMSRGVDFESDE.....D...DP....FKN.TATSRRKK......................... 700
XP 029898375.1 double-strand break repair protein MRE11 isoform X1 Aquila chrysaetos chrysaetos  ..SKSYSEDIM.DDDSDL.........EDISIV.RSSKVNQRL......STTSS..............LSKRGSQSQTSRGVDFESDE.....DEF.DP....FKS.TAPSRRTK......................... 706
XP 002198371.1 double-strand break repair protein MRE11 isoform X2 Taeniopygia guttata  ..FQSYSEDIV.DVDSDP.........EDTSFVPPSSKINQRL......PTMSS..............LSKRGSQSQASRGVDFESDE.....D...DP....FMS.PATSRRTK......................... 704
XP 030905430.2 LOW QUALITY PROTEIN: double-strand break repair protein MRE11 Melopsittacus undulatus  ..SKSYSEDII.DDDSDV.........EQVSTT.SSSKVSLRL......SSTPS..............LLKRGSQSQTSRGVDFESDE.....GEF.DP....FQS.TATSRRK.......................... 716
XP 019339178.1 PREDICTED: double-strand break repair protein MRE11A Alligator mississippiensis  ..AKNYSEHIV.DDDSDV.........EEIPFT..ASKVNQRV......SSTST..............FSKTGSQSQMSKGVDFESDE.....DEY.DP....FKN.TTVSRRNRR........................ 712
XP 023966648.1 double-strand break repair protein MRE11 isoform X1 Chrysemys picta bellii  ..AKNYSEDII.DDDSDV.........EEVSFT.AASKFTQRL......SSTST..............SSKRSSQSQITRGVDFESDE.....DEF.DP....FKS.TATSRRKHR........................ 711
XP 007435918.1 double-strand break repair protein MRE11 isoform X1 Python bivittatus  ..IKNYSEVIE.DDDSDI.........EEVSFT.PSSTTNQRL......PTTST..............YSQRSSQSQSSKGVEFESDE.....D...EL....F...VSTSRRNR......................... 704
XP 013928211.1 PREDICTED: double-strand break repair protein MRE11A Thamnophis sirtalis  ..VKNYSEVIE.DDDSDV.........EMSC...SSSSTNQRL......PTAST..............QSQRNSQSQASKGVSFESDE.....D...EL....F...VSTSRRKR......................... 701
NP 001107519.1 double-strand break repair protein MRE11 Xenopus tropicalis  ..KKTYSEDIE.DDDSDI.........EEVSFT.PSSFVESRRT.....SSTST............SYSRKSSQSQAAKGHFLDDDDD...EDEF.DP....FKK.SAPSRRGRR........................ 709
XP 018412310.1 PREDICTED: double-strand break repair protein MRE11A Nanorana parkeri  ..AKNYSEEIE.DNDSDL.........EEVSFT.AASHKNSRA......SSTSS............SFSKNSSQSQPSQAAFFIDDDD...EEER.SSTGCILEA.KEESSKGRCLSEEQYRRGTLEERNSTKGKLEKK 715
XP 006008507.1 PREDICTED: double-strand break repair protein MRE11A Latimeria chalumnae  ..TKNYAEVIV.DDDSDM.........EEEDSF.CSSKVDQRP..........S................SKRSSQGQISRGVHFDNNDDSDDEDEF.DP....FKSAASSSRRGRR....................... 710
NP 001307188.1 double-strand break repair protein MRE11 Danio rerio  ..PKSYAEEIT.IEDSDS........DEDVSFL.KSSRTKQKL......PSLSD................SKRASQSQSLKGVCIDEDSG...EDEF.DP....FKG.SRKQRR........................... 700
XP 030193234.1 double-strand break repair protein MRE11 isoform X1 Gadus morhua  ..AKSYTEEVV.IDDSD..........EDFPTA.ASSGPPPRS......SASQAPPRGSASQAPPRGSSFQSSSESQSSRGVAFDDSDD...DDEE.DP....FKA.QRSRTRR.......................... 703
XP 007900243.1 PREDICTED: double-strand break repair protein MRE11A Callorhinchus milii  ..QSNYTEIISTDDDSDL.........EDVSSS.SASKTKQRG......SSMSE............SFSKRGSQSLAAKGVTFYDDDE...EGED.DP....FKN.STSSRQNRR........................ 719
XP 032833686.1 double-strand break repair protein MRE11 Petromyzon marinus  V.TKKYNPPVQ.ISDSES........ETELLSA.SPKRSSERNRNEVTGPAGSS...........RAPSGSYRTSSQSHRGVMFDDDDD...EDEDYNP....FK................................. 707
XP 030833624.1 double-strand break repair protein MRE11 Strongylocentrotus purpuratus  E..........DDDDDD........DMEVSMP.ASR..............................GSRAPKATKNKVINISDSSD...NDSE.DP....FSA.MSSSSRSQA.....................KRS 688
NP 523547.1 meiotic recombination 11 Drosophila melanogaster  ..SRNTASYVI.SDDSD........................................................................................................................ 620
XP 016767519.2 double-strand break repair protein MRE11 Apis mellifera  .........................ESNLNLC...............................................................QET......................................... 630
NP 505736.2 Double-strand break repair protein mre-11 Caenorhabditis elegans  ............INDSPS...................................................PPPASRSTRGKARGKSAP...S................KKRDLSF...................F... 728
XP 005090246.1 PREDICTED: double-strand break repair protein MRE11A-like Aplysia californica  ..SAAGGDFTV.ISDDDD........DSEFSIS.SARGTNQRS..........................TQRSRGKKGPVFIEDSS...EDEL..P....VRK.....RRK.......................... 689
NP 013951.1 MRX complex nuclease subunit Saccharomyces cerevisiae S288C  GDNNDAQDDVD.IDENDIIMVSTDEEDASYGLL.NGRKTKTKT.......................RPAA.STKTASRRGKGRASRTP...K..T.DI....LGS.LLAKKRK.......................... 692
NP 592935.1 Rad32 nuclease Schizosaccharomyces pombe  ..STTEAQYLD.NEDDEIL.................................................................D...D............................................. 649
```

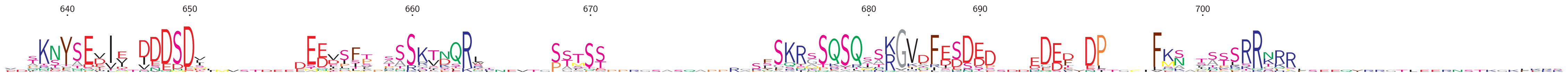

```
logo

HsMRE11                                                                          ................................................................................ 708
NP 061206.1 double-strand break repair protein MRE11 isoform 1 Mus musculus     ................................................................................ 706
XP 003129836.2 LOW QUALITY PROTEIN: double-strand break repair protein MRE11 Sus scrofa  ................................................................................ 708
XP 001513434.2 double-strand break repair protein MRE11 Ornithorhynchus anatinus  ................................................................................ 707
XP 023410492.1 double-strand break repair protein MRE11 isoform X2 Loxodonta africana  ................................................................................ 709
XP 015135386.1 double-strand break repair protein MRE11 isoform X1 Gallus gallus  ................................................................................ 700
XP 029898375.1 double-strand break repair protein MRE11 isoform X1 Aquila chrysaetos chrysaetos  ................................................................................ 706
XP 002198371.1 double-strand break repair protein MRE11 isoform X2 Taeniopygia guttata  ................................................................................ 704
XP 030905430.2 LOW QUALITY PROTEIN: double-strand break repair protein MRE11 Melopsittacus undulatus  ................................................................................ 716
XP 019339178.1 PREDICTED: double-strand break repair protein MRE11A Alligator mississippiensis  ................................................................................ 712
XP 023966648.1 double-strand break repair protein MRE11 isoform X1 Chrysemys picta bellii  ................................................................................ 711
XP 007435918.1 double-strand break repair protein MRE11 isoform X1 Python bivittatus  ................................................................................ 704
XP 013928211.1 PREDICTED: double-strand break repair protein MRE11A Thamnophis sirtalis  ................................................................................ 701
NP 001107519.1 double-strand break repair protein MRE11 Xenopus tropicalis  ................................................................................ 709
XP 018412310.1 PREDICTED: double-strand break repair protein MRE11A Nanorana parkeri  KRNSTRENWRWRRGVKQEGNLGGEEQCKENAGGGREEQYRRDSEDEEQQRVTLMWKEINRTGEHCGKKQRGTTGENES 793
XP 006008507.1 PREDICTED: double-strand break repair protein MRE11A Latimeria chalumnae  ................................................................................ 710
NP 001307188.1 double-strand break repair protein MRE11 Danio rerio  ................................................................................ 700
XP 030193234.1 double-strand break repair protein MRE11 isoform X1 Gadus morhua  ................................................................................ 703
XP 007900243.1 PREDICTED: double-strand break repair protein MRE11A Callorhinchus milii  ................................................................................ 719
XP 032833686.1 double-strand break repair protein MRE11 Petromyzon marinus  ................................................................................ 707
XP 030833624.1 double-strand break repair protein MRE11 Strongylocentrotus purpuratus  RRN............................................................................. 691
NP 523547.1 meiotic recombination 11 Drosophila melanogaster  ................................................................................ 620
XP 016767519.2 double-strand break repair protein MRE11 Apis mellifera  ................................................................................ 630
NP 505736.2 Double-strand break repair protein mre-11 Caenorhabditis elegans  ................................................................................ 728
XP 005090246.1 PREDICTED: double-strand break repair protein MRE11A-like Aplysia californica  ................................................................................ 689
NP 013951.1 MRX complex nuclease subunit Saccharomyces cerevisiae S288C  ................................................................................ 692
NP 592935.1 Rad32 nuclease Schizosaccharomyces pombe  ................................................................................ 649
```

X non-conserved
X similar
X ≥98% conserved

B. RAD50

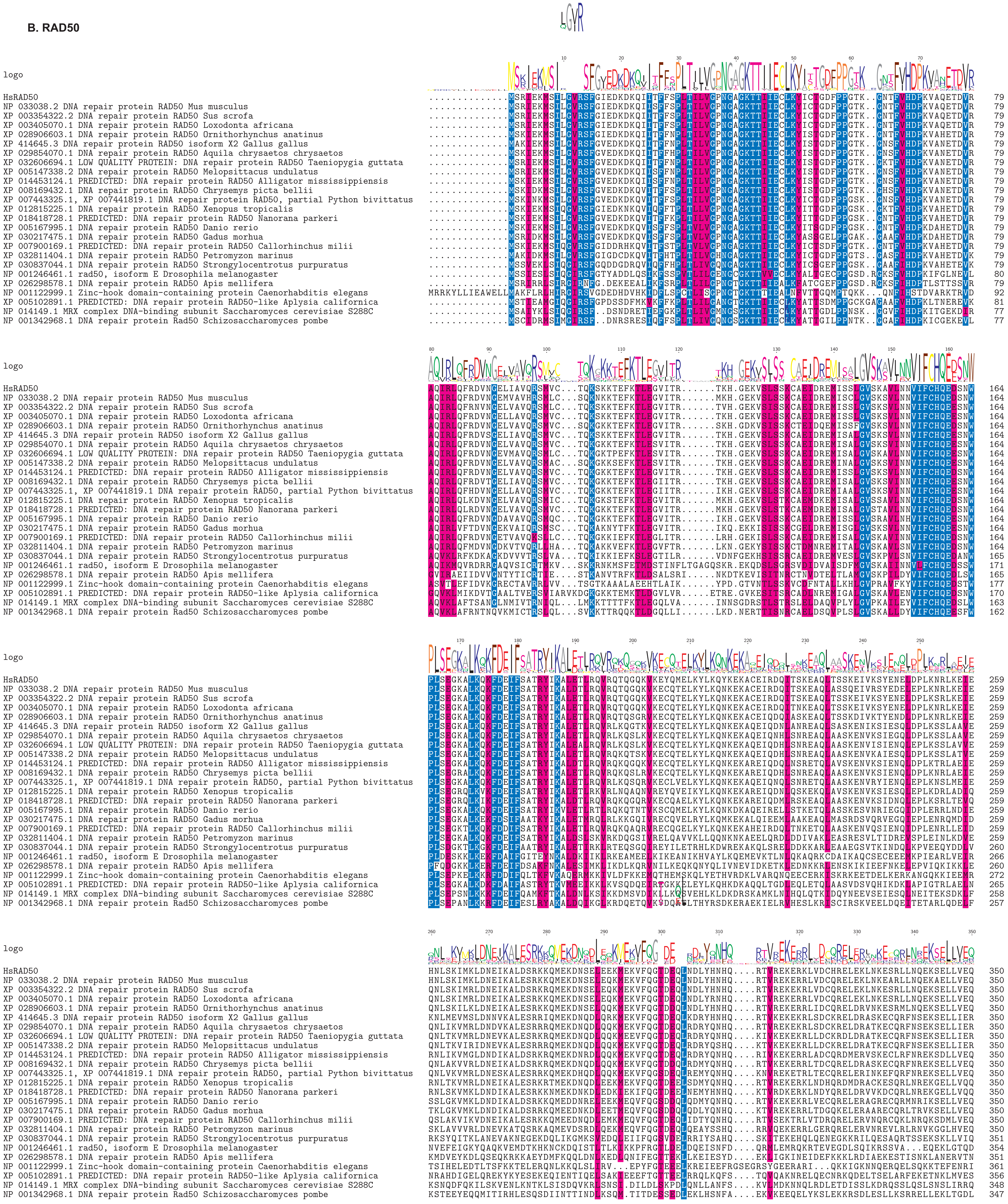
logo
HsRAD50
NP 033038.2 DNA repair protein RAD50 Mus musculus
XP 003354322.2 DNA repair protein RAD50 Sus scrofa
XP 003405070.1 DNA repair protein RAD50 Loxodonta africana
XP 028906603.1 DNA repair protein RAD50 Ornithorhynchus anatinus
XP 414645.3 DNA repair protein RAD50 isoform X2 Gallus gallus
XP 029854070.1 DNA repair protein RAD50 Aquila chrysaetos chrysaetos
XP 032606694.1 LOW QUALITY PROTEIN: DNA repair protein RAD50 Taeniopygia guttata
XP 005147338.2 DNA repair protein RAD50 Melopsittacus undulatus
XP 014453124.1 PREDICTED: DNA repair protein RAD50 Alligator mississippiensis
XP 008169432.1 DNA repair protein RAD50 Chrysemys picta bellii
XP 007443325.1, XP 007441819.1 DNA repair protein RAD50, partial Python bivittatus
XP 012815225.1 DNA repair protein RAD50 Xenopus tropicalis
XP 018418728.1 PREDICTED: DNA repair protein RAD50 Nanorana parkeri
XP 005167995.1 DNA repair protein RAD50 Danio rerio
XP 030217475.1 DNA repair protein RAD50 Gadus morhua
XP 007900169.1 PREDICTED: DNA repair protein RAD50 Callorhinchus milii
XP 032811404.1 DNA repair protein RAD50 Petromyzon marinus
XP 030837044.1 DNA repair protein RAD50 Strongylocentrotus purpuratus
NP 001246461.1 rad50, isoform E Drosophila melanogaster
XP 026298578.1 DNA repair protein RAD50 Apis mellifera
NP 001122999.1 Zinc-hook domain-containing protein Caenorhabditis elegans
XP 005102891.1 PREDICTED: DNA repair protein RAD50-like Aplysia californica
NP 014149.1 MRX complex DNA-binding subunit Saccharomyces cerevisiae S288C
NP 001342968.1 DNA repair protein Rad50 Schizosaccharomyces pombe

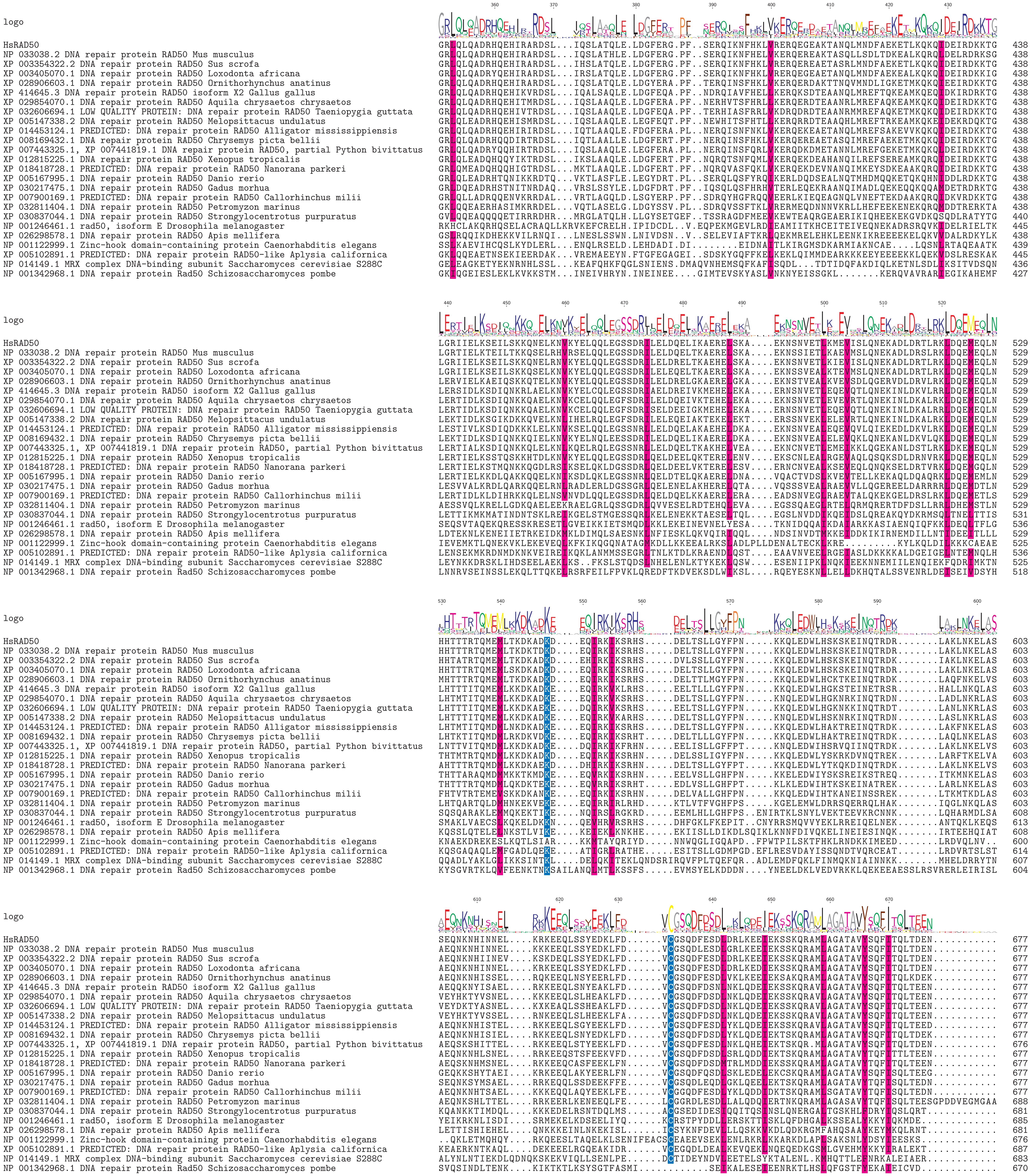
logo
HsRAD50
NP 033038.2 DNA repair protein RAD50 Mus musculus 438
XP 003354322.2 DNA repair protein RAD50 Sus scrofa 438
XP 003405070.1 DNA repair protein RAD50 Loxodonta africana 438
XP 028906603.1 DNA repair protein RAD50 Ornithorhynchus anatinus 438
XP 414645.3 DNA repair protein RAD50 isoform X2 Gallus gallus 438
XP 029854070.1 DNA repair protein RAD50 Aquila chrysaetos chrysaetos 438
XP 032606694.1 LOW QUALITY PROTEIN: DNA repair protein RAD50 Taeniopygia guttata 438
XP 005147338.2 DNA repair protein RAD50 Melopsittacus undulatus 438
XP 014453124.1 PREDICTED: DNA repair protein RAD50 Alligator mississippiensis 438
XP 008169432.1 DNA repair protein RAD50 Chrysemys picta bellii 438
XP 007443325.1, XP 007441819.1 DNA repair protein RAD50, partial Python bivittatus 438
XP 012815225.1 DNA repair protein RAD50 Xenopus tropicalis 438
XP 018418728.1 PREDICTED: DNA repair protein RAD50 Nanorana parkeri 438
XP 005167995.1 DNA repair protein RAD50 Danio rerio 438
XP 030217475.1 DNA repair protein RAD50 Gadus morhua 438
XP 007900169.1 PREDICTED: DNA repair protein RAD50 Callorhinchus milii 438
XP 032811404.1 DNA repair protein RAD50 Petromyzon marinus 438
XP 030837044.1 DNA repair protein RAD50 Strongylocentrotus purpuratus 440
NP 001246461.1 rad50, isoform E Drosophila melanogaster 445
XP 026298578.1 DNA repair protein RAD50 Apis mellifera 438
NP 001122999.1 Zinc-hook domain-containing protein Caenorhabditis elegans 439
XP 005102891.1 PREDICTED: DNA repair protein RAD50-like Aplysia californica 445
NP 014149.1 MRX complex DNA-binding subunit Saccharomyces cerevisiae S288C 436
NP 001342968.1 DNA repair protein Rad50 Schizosaccharomyces pombe 427
logo
HsRAD50
NP 033038.2 DNA repair protein RAD50 Mus musculus 529
XP 003354322.2 DNA repair protein RAD50 Sus scrofa 529
XP 003405070.1 DNA repair protein RAD50 Loxodonta africana 529
XP 028906603.1 DNA repair protein RAD50 Ornithorhynchus anatinus 529
XP 414645.3 DNA repair protein RAD50 isoform X2 Gallus gallus 529
XP 029854070.1 DNA repair protein RAD50 Aquila chrysaetos chrysaetos 529
XP 032606694.1 LOW QUALITY PROTEIN: DNA repair protein RAD50 Taeniopygia guttata 529
XP 005147338.2 DNA repair protein RAD50 Melopsittacus undulatus 529
XP 014453124.1 PREDICTED: DNA repair protein RAD50 Alligator mississippiensis 529
XP 008169432.1 DNA repair protein RAD50 Chrysemys picta bellii 529
XP 007443325.1, XP 007441819.1 DNA repair protein RAD50, partial Python bivittatus 529
XP 012815225.1 DNA repair protein RAD50 Xenopus tropicalis 529
XP 018418728.1 PREDICTED: DNA repair protein RAD50 Nanorana parkeri 529
XP 005167995.1 DNA repair protein RAD50 Danio rerio 529
XP 030217475.1 DNA repair protein RAD50 Gadus morhua 529
XP 007900169.1 PREDICTED: DNA repair protein RAD50 Callorhinchus milii 529
XP 032811404.1 DNA repair protein RAD50 Petromyzon marinus 529
XP 030837044.1 DNA repair protein RAD50 Strongylocentrotus purpuratus 531
NP 001246461.1 rad50, isoform E Drosophila melanogaster 536
XP 026298578.1 DNA repair protein RAD50 Apis mellifera 529
NP 001122999.1 Zinc-hook domain-containing protein Caenorhabditis elegans 525
XP 005102891.1 PREDICTED: DNA repair protein RAD50-like Aplysia californica 536
NP 014149.1 MRX complex DNA-binding subunit Saccharomyces cerevisiae S288C 525
NP 001342968.1 DNA repair protein Rad50 Schizosaccharomyces pombe 518
logo
HsRAD50
NP 033038.2 DNA repair protein RAD50 Mus musculus 603
XP 003354322.2 DNA repair protein RAD50 Sus scrofa 603
XP 003405070.1 DNA repair protein RAD50 Loxodonta africana 603
XP 028906603.1 DNA repair protein RAD50 Ornithorhynchus anatinus 603
XP 414645.3 DNA repair protein RAD50 isoform X2 Gallus gallus 603
XP 029854070.1 DNA repair protein RAD50 Aquila chrysaetos chrysaetos 603
XP 032606694.1 LOW QUALITY PROTEIN: DNA repair protein RAD50 Taeniopygia guttata 603
XP 005147338.2 DNA repair protein RAD50 Melopsittacus undulatus 603
XP 014453124.1 PREDICTED: DNA repair protein RAD50 Alligator mississippiensis 603
XP 008169432.1 DNA repair protein RAD50 Chrysemys picta bellii 603
XP 007443325.1, XP 007441819.1 DNA repair protein RAD50, partial Python bivittatus 603
XP 012815225.1 DNA repair protein RAD50 Xenopus tropicalis 603
XP 018418728.1 PREDICTED: DNA repair protein RAD50 Nanorana parkeri 603
XP 005167995.1 DNA repair protein RAD50 Danio rerio 603
XP 030217475.1 DNA repair protein RAD50 Gadus morhua 603
XP 007900169.1 PREDICTED: DNA repair protein RAD50 Callorhinchus milii 603
XP 032811404.1 DNA repair protein RAD50 Petromyzon marinus 603
XP 030837044.1 DNA repair protein RAD50 Strongylocentrotus purpuratus 608
NP 001246461.1 rad50, isoform E Drosophila melanogaster 613
XP 026298578.1 DNA repair protein RAD50 Apis mellifera 608
NP 001122999.1 Zinc-hook domain-containing protein Caenorhabditis elegans 600
XP 005102891.1 PREDICTED: DNA repair protein RAD50-like Aplysia californica 614
NP 014149.1 MRX complex DNA-binding subunit Saccharomyces cerevisiae S288C 607
NP 001342968.1 DNA repair protein Rad50 Schizosaccharomyces pombe 604
logo
HsRAD50
NP 033038.2 DNA repair protein RAD50 Mus musculus 677
XP 003354322.2 DNA repair protein RAD50 Sus scrofa 677
XP 003405070.1 DNA repair protein RAD50 Loxodonta africana 677
XP 028906603.1 DNA repair protein RAD50 Ornithorhynchus anatinus 677
XP 414645.3 DNA repair protein RAD50 isoform X2 Gallus gallus 677
XP 029854070.1 DNA repair protein RAD50 Aquila chrysaetos chrysaetos 677
XP 032606694.1 LOW QUALITY PROTEIN: DNA repair protein RAD50 Taeniopygia guttata 677
XP 005147338.2 DNA repair protein RAD50 Melopsittacus undulatus 677
XP 014453124.1 PREDICTED: DNA repair protein RAD50 Alligator mississippiensis 677
XP 008169432.1 DNA repair protein RAD50 Chrysemys picta bellii 677
XP 007443325.1, XP 007441819.1 DNA repair protein RAD50, partial Python bivittatus 676
XP 012815225.1 DNA repair protein RAD50 Xenopus tropicalis 677
XP 018418728.1 PREDICTED: DNA repair protein RAD50 Nanorana parkeri 677
XP 005167995.1 DNA repair protein RAD50 Danio rerio 677
XP 030217475.1 DNA repair protein RAD50 Gadus morhua 677
XP 007900169.1 PREDICTED: DNA repair protein RAD50 Callorhinchus milii 677
XP 032811404.1 DNA repair protein RAD50 Petromyzon marinus 688
XP 030837044.1 DNA repair protein RAD50 Strongylocentrotus purpuratus 681
NP 001246461.1 rad50, isoform E Drosophila melanogaster 685
XP 026298578.1 DNA repair protein RAD50 Apis mellifera 681
NP 001122999.1 Zinc-hook domain-containing protein Caenorhabditis elegans 676
XP 005102891.1 PREDICTED: DNA repair protein RAD50-like Aplysia californica 687
NP 014149.1 MRX complex DNA-binding subunit Saccharomyces cerevisiae S288C 683
NP 001342968.1 DNA repair protein Rad50 Schizosaccharomyces pombe 670

logo

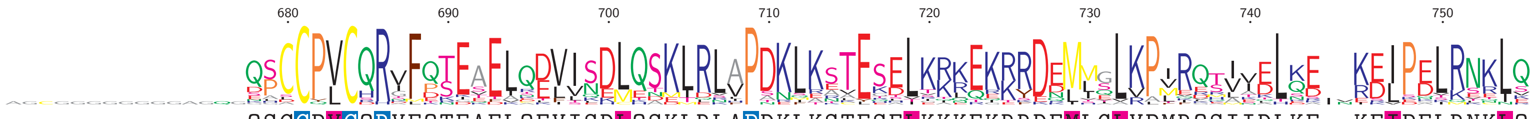

```
HsRAD50                                                                  ...............QSCCPVCQRVFQTEAELQEVISDLQSKLRLAPDKLKSTESELKKKEKRRDEMLGLVPMRQSIIDLKE..KEIPELRNKLQ   755
NP 033038.2 DNA repair protein RAD50 Mus musculus                        ...............QSCCPVCQRVFQTEAELQEVISDLQSKLRLAPDKLKSTESELKKKERRRDEMLGLVPVRQSIIDLKE..KEIPELRNRLQ   755
XP 003354322.2 DNA repair protein RAD50 Sus scrofa                       ...............QSCCPVCQRVFQTEAELQEVISDLQSKLRLAPDKLKSTESELKKKEKRRDEMLGLVPMRQSIIDLKE..KEIPALRNKLQ   755
XP 003405070.1 DNA repair protein RAD50 Loxodonta africana               ...............QSCCPVCQRVFQTETELQEVISDLQSKLRLAPDKLKSTESELKKKEKRRDDMLGLVPMRQSIVDLRE..KEIPELRNKLQ   755
XP 028906603.1 DNA repair protein RAD50 Ornithorhynchus anatinus         ...............QSCCPVCQRVFQTEAELRDVISDLESKLRLAPDKLKSTESDLKRKEKRRDDMVGLKPIRQHIVELKE..KEIPELRNKLQ   755
XP 414645.3 DNA repair protein RAD50 isoform X2 Gallus gallus             ...............QSCCPVCQRVFQTEAELQDVISDLQSKLRLAPDKLKSTESELKKREKKRDEMIGLKPIRQTVVELQE..RDIPDLRNRLQ   755
XP 029854070.1 DNA repair protein RAD50 Aquila chrysaetos chrysaetos     ...............QSCCPVCQRVFQTEAELQDVISDLQSKLRLAPDKLKSTESELKRKEKKRDEMMSLKPLRRTVVELQD..RDIPDLRSKIQ   755
XP 032606694.1 LOW QUALITY PROTEIN: DNA repair protein RAD50 Taeniopygia guttata ...............QSCCPVCQRVFQTEAELQDVISDLQSKLRLAPDKLKSTESELKRKEKKRDEMMSLKPLRRTVAELQD..KEIPDLRSKIQ   755
XP 005147338.2 DNA repair protein RAD50 Melopsittacus undulatus          ...............QLCCPVCQRGFETEAELQDVISDLQSKLRLAPDKLKSTESELKRKEKKRDEMMSLKPLRQTVVELQA..RDIPDLRNKIQ   755
XP 014453124.1 PREDICTED: DNA repair protein RAD50 Alligator mississippiensis ...............QACCPVCQRVFQTETELQDVISDLQSKLRLAPDKLKTTESELKRREKRRDEMMGLKPIRQTIVELKE..KDIPDLRNKLQ   755
XP 008169432.1 DNA repair protein RAD50 Chrysemys picta bellii          ...............QSCCPVCQRVFQTESELEDVISDLQSKLRLAPDKLKSTESELKRREKKRDEMMGLKPIRQTIAELKE..KDIPDLRNKLQ   755
XP 007443325.1, XP 007441819.1 DNA repair protein RAD50, partial Python bivittatus ...............QSCCPVCQRIFQTEAELQDVINDLQAKLRLAPDKLKATESELKKREKKRDDMISLKPFRQTVSELQE..KDIPELRNKLQ   754
XP 012815225.1 DNA repair protein RAD50 Xenopus tropicalis              ...............QPCCPVCQRIFPSEAELQDVINDMQSKLRLVPDKLKAAEGELKRKEKRKDEMMELKPMRQMLADLKE..KEVPEIRNKLV   755
XP 018418728.1 PREDICTED: DNA repair protein RAD50 Nanorana parkeri     ...............QPCCPVCQRIFPSEAELQDVINDMQSKLRLVPDKLKSTEGDLKKREKRRDDMMELKPMRQMLIDHKE..KEIPELRNKLV   755
XP 005167995.1 DNA repair protein RAD50 Danio rerio                     ...............DPCCPVCQRVFPSEAELQDVINDMQSKLRLVPDKLKNTEHDLKRKERRRDEMMTLKPIRQSLVELQE..KELPELRNQLQ   755
XP 030217475.1 DNA repair protein RAD50 Gadus morhua                    ...............GPCCPVCQRTFPSEVELDEVVSDMQSKLRLVPDKLKNTEQELRRKEGRRDQMMALRPVRVSMEKLQK..EELPELRNRLQ   755
XP 007900169.1 PREDICTED: DNA repair protein RAD50 Callorhinchus milii  ...............EPCCPVCQRIFPSEAELQDIVNDLQSKLRLVPDKLKATESELKRREKRRDEMMGLKPVRETVTELNE..RDLPELRNKLQ   755
XP 032811404.1 DNA repair protein RAD50 Petromyzon marinus              AGCGGGGGGGGAGQGSPCCPVCQRAFQSESDLAEVVNDLQAKLRLVPDKLRATETELTRKQKRRDNMIQLKPVQQRVVETSE..KELPELRNRLS   781
XP 030837044.1 DNA repair protein RAD50 Strongylocentrotus purpuratus   ...............DALCPLCHRGFDSGVEVQELVQELQDKLRLAPSKLAEKERTITKQREKSDRLLVLRPIKDAAKKLSE..REIPDLKLKLS   759
NP 001246461.1 rad50, isoform E Drosophila melanogaster                ...............EPSCPLCHHNMTSD.EACDLTSELTDEIQKLPDNITRAEKALKAEQIKYENLLQLKPTILKVKELKD...SLPQKKEELK   761
XP 026298578.1 DNA repair protein RAD50 Apis mellifera                  ...............NPCCPLCHRGFDKIETITTLLKEMETEIENHPNRLKECEKELIIQQEKYDKMLQLKPVVERIIQLEE..IELEKLMSNLE   759
NP 001122999.1 Zinc-hook domain-containing protein Caenorhabditis elegans ...............SGCCPLCDRDFKTKKEINEFSKKLENMTLSFPTEQEELEKLVSKLEK..EEIIIVKA.EGQANELQRIVKELKEVREKNR   753
XP 005102891.1 PREDICTED: DNA repair protein RAD50-like Aplysia californica ...............KPDCPLCRRGFQREQEARELILKLQNDMRRVPANMRRVEESLGRLQQRYDQLTQLKAVRETATALKD..RDIPGLKVTLK   765
NP 014149.1 MRX complex DNA-binding subunit Saccharomyces cerevisiae S288C ...............DSCCYLCSRKFENESFKSKLLQELKTKTD...ANFEKTLKDTVQNEKEY..LHSLRLLEKHIITLNSINEKIDNSQKCLE   758
NP 001342968.1 DNA repair protein Rad50 Schizosaccharomyces pombe       ...............QHACQLCQRSLDKEEE.KLFVEHCHSMIDVIPSKSAEVYSHLETLTKTFKNLSEAKPIFDEIELLD...KRLSETKTELS   746
```

logo

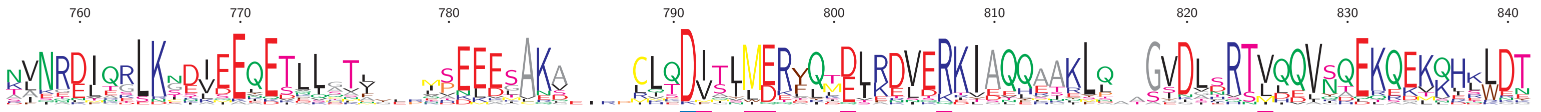

```
HsRAD50                                                                  NVNRDIQRLKNDIEEQETLLGTI...MPEEESAKV....CLTDVTIMERFQMELKDVERKIAQQAAKLQ..GIDLDRTVQQVNQEKQEKQHKLDT   841
NP 033038.2 DNA repair protein RAD50 Mus musculus                        SVNRDIQRLKNDIEEQETLLGTI...MPEEESAKV....CLTDVTIMERFQMELKDVERKIAQQAAKLQ..GVDLDRTVQQVNQEKQEKQHRLDT   841
XP 003354322.2 DNA repair protein RAD50 Sus scrofa                       NVNRDIQSLKIDIEEQDTLLGAI...IPEEESAKV....CLTDVTIMERFQLELKDVERKIAQQAAKLQ..GVDLHRTIQQVNQEKQEKQHKLDT   841
XP 003405070.1 DNA repair protein RAD50 Loxodonta africana               NVNRDIQRLKNDIEEQETLLGTI...MPEEESAKV....CLTDVTIMERLQMELKDVERKIAQQAAKLQ..GIDLDRTVQQVNQDKQEKQHKLDT   841
XP 028906603.1 DNA repair protein RAD50 Ornithorhynchus anatinus         TLNRDIQRLKCDVEEQETVLCTV...MPEEESAKA....CLTDITIMERVQMELRDVERKIAQQAAKLQ..GVDLDRTVQQVNQEKQEKQHKLDT   841
XP 414645.3 DNA repair protein RAD50 isoform X2 Gallus gallus             TVNRDFARLKGEIEEQETLLQTV...LSEKEGANA....CLQDITLMERYQTDIRDVERKIAQQEAKLL..GVDLNRTVLQVSQEKQAKKHLWDT   841
XP 029854070.1 DNA repair protein RAD50 Aquila chrysaetos chrysaetos     NANRDLTGLKGEIEEQESLLRTA...LSEEDGAKA....RLQDITLMERYQTDIRDVERKIAQQEAKLL..GVDLSRTVLQVSHEKQEKKHLWDT   841
XP 032606694.1 LOW QUALITY PROTEIN: DNA repair protein RAD50 Taeniopygia guttata NANRDLTGLKGEIEEQESLLQTA...LSEEEGAKA....RLQDITLMERYQTDIRDVERKIAQQEAKLL..GVNLSRTVHQVSKEKQDKKHLWDT   841
XP 005147338.2 DNA repair protein RAD50 Melopsittacus undulatus          NANRDLTGLKGEIEEQESLLQTA...LSEEEGAKA....RLQDITLMERYQTDIRDVDRKIAQQEAKLL..GVDLSRTVLQVSQEKQEKKHLWDT   841
XP 014453124.1 PREDICTED: DNA repair protein RAD50 Alligator mississippiensis NVNRDIQRLKTDIEEVETLLHTV...IPEEESAKA....CLQDITLMERYQIDLRDVERKIAQHAAKLL..GVDLTRTVQQVSQEKQGKKHLWDT   841
XP 008169432.1 DNA repair protein RAD50 Chrysemys picta bellii          NVNRDIQRLKSDVEEQETLLGTV...MPEEESAKA....CLQDITLMERYQTDLRDVERKIAQQANKLQ..GVDLDRTLIQVSQEKQEKKHLWDT   841
XP 007443325.1, XP 007441819.1 DNA repair protein RAD50, partial Python bivittatus TLNREIQSLKSDIEEQEIFLGVN...ITEEESAKA....CLQDIIIMERDQTDLKDVERKIAQQATKLQ..GVDLVRTLLQVSQEKQEKKHQWDT   840
XP 012815225.1 DNA repair protein RAD50 Xenopus tropicalis              AINREIQRLKNDVEEQETLIATF...VSEEESAKA....CLQDISLMERYQMELRDVERKIAQYATKLQ..GVDLNRTVQQVNQEKQEKQHSLDN   841
XP 018418728.1 PREDICTED: DNA repair protein RAD50 Nanorana parkeri     TINREIQRLKNDVEEQETLIATF...MSEEDSAKA....CLQDVSLMERYQMELRDVERKIAQHATKLQ..GVDLNRSVQQVNQEKQEKQLTLDN   841
XP 005167995.1 DNA repair protein RAD50 Danio rerio                     RVNRDIEKLKGDIEEQETLLCTL...VSEEETAKA....CLQDISLMDRFQLDLKDVERKIAQHAAKLQ..GVDLSRTMQQVSQEKQETQHRLDT   841
XP 030217475.1 DNA repair protein RAD50 Gadus morhua                    AVNRNIERLKGDVEEQETGLSVM...ISEEETAKA....CLQDIALMDRFLLDLKEVDRKIAQQEARLQ..GVDLSRTVQQVSQEKQETQHRLDT   841
XP 007900169.1 PREDICTED: DNA repair protein RAD50 Callorhinchus milii  NIIRDIQRLKCDVEEQETLQAAF...ISEEGSAKQ....CLQDISLMDRYQMELRDVERKIAQQAAKLQ..GVDVSRTMQQVNQEKQEMQHKLET   841
XP 032811404.1 DNA repair protein RAD50 Petromyzon marinus              SLNQEIQRLKNSLDEEETLQATL...ESEEDAVKS....CQQDVALMDRFQSDLRELERKVAQQMARLA..GVDVERTLQVVGDEKQDVQRKLDN   867
XP 030837044.1 DNA repair protein RAD50 Strongylocentrotus purpuratus   AVNADIEKLNATVMEAEDNVSML...QVDEAMARD....MKPDIQILEKTLTELRDLERKIGEQSKYFS..GSDISRTLQQVNTETAEVKIKLNT   845
NP 001246461.1 rad50, isoform E Drosophila melanogaster                KVEELLGD...SVSEYETLIALIGEPTHNMELANS....MMGDMSLLDEALKDSARLTKDLDLQKGQLP.ASYDSSVSMDDLQAEKSKVSKELET   848
XP 026298578.1 DNA repair protein RAD50 Apis mellifera                  KFKNKLNESRTIAIELKTKKSNP...EKKLVMCKD....IISDVMLWDTYIDDIFKLKQIIDNFHIRMTAAGIKTKRSLQEAQTEREELKISLKK   847
NP 001122999.1 Zinc-hook domain-containing protein Caenorhabditis elegans KLSTEMAEEKSNLSKNEKQLETV...NAKLKLAED....LQTDVGVIQQLYEQTEENEKRYEQLVSESD..SSDGLS.....YTELRKKVEDKDE   834
XP 005102891.1 PREDICTED: DNA repair protein RAD50-like Aplysia californica KVEGEVRGLKEEKQAKEDALMSL...QVDLDIAKG....LHSDVVSMDRYRGEVRELQRKVETQRALLP..AGGATRSVHDVIALQEEKQLELDG   851
NP 014149.1 MRX complex DNA-binding subunit Saccharomyces cerevisiae S288C KAKEETKTSKSKLDELEVDSTKL...KDEKELAESEIRPLIEKFTYLEKELKDLENSSKTISEELSIYN.TSEDGIQTVDELRDQQRKMNDSLRE   849
NP 001342968.1 DNA repair protein Rad50 Schizosaccharomyces pombe       DLQGDLQGLDIRKDEIQSELDTLYLRRANLEKLQL....LVKDISNLEE...EIRTIDRETEVLRIELP..SSIAHHNLDEIYAEREKLLEKRGY   832
```

logo

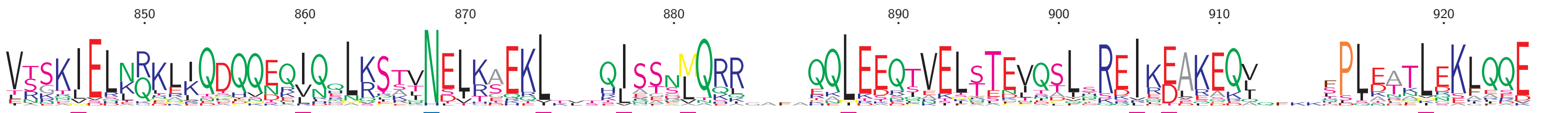

```
HsRAD50                                                                  VSSKIELNRKLIQDQQEQIQHLKSTTNELKSEKL...QISTNLQRR....QQLEEQTVELSTEVQSLYREIKDAKEQV....SPLETTLEKFQQE   925
NP 033038.2 DNA repair protein RAD50 Mus musculus                        VTSKIELNRKLIQDQQEQIQHLKSKTNELKSEKL...QIATNLQRR....QQMEEQSVELSTEVQSLNREIKDAKEQI....SPLETALEKLQQE   925
XP 003354322.2 DNA repair protein RAD50 Sus scrofa                       VASKIELNRKLIQDQQEQIQHLKSTTNELKSEKL...QISTNLQRR....QQLEEQTVELSTEVQSLQREIKDAKEQV....NPLETTLEKFQQE   925
XP 003405070.1 DNA repair protein RAD50 Loxodonta africana               VSSKIELNRKLIQDQQEQVQQLKSTTNELKSEKL...QISTNLQRR....QQLEEQTVELSTEVQSLYREIKDAKEQV....SPLETTLEKFQQE   925
XP 028906603.1 DNA repair protein RAD50 Ornithorhynchus anatinus         VSSKMELNRKLIQDQQEQIQGLKSTVNDLKSEKL...QISSNLQRR....QQLEEQTVELSTEVQSLHREIKDAKEQL....FPLEATLDKLRQE   925
XP 414645.3 DNA repair protein RAD50 isoform X2 Gallus gallus             VTSKIELNQKMKQDQQNQIQELKSTVNELRAEKL...QISSSVQRR....QQLEEQTVELTTEVQSLSREIKEEKEQV....FPLDATLEKLQQD   925
XP 029854070.1 DNA repair protein RAD50 Aquila chrysaetos chrysaetos     VTGKIELNQKLKQDQQNQIQQLKSAVNELKAEKL...QISSSMQHR....RQLEEQTVELTTEVQSLCREIKEAKEQV....FPLNATLEKLQQE   925
XP 032606694.1 LOW QUALITY PROTEIN: DNA repair protein RAD50 Taeniopygia guttata VTSKIELNQKLKQDQQSQIQQLRSAVNDLKAEKL...QIVSSMQRR....RQLEEQTVELTTEVQALCREIKEAEEQV....LPLDAKLDKLQQE   925
XP 005147338.2 DNA repair protein RAD50 Melopsittacus undulatus          VTGKIELNQKLKQDQQNQIQQLKSTINELKAEKL...QISSSMQRR....HQLEEQTVELSTEVQALSREIKEAKEQV....LPLDTTLEKLQQE   925
XP 014453124.1 PREDICTED: DNA repair protein RAD50 Alligator mississippiensis VTSKIELNQKLKQAQQNEIQQLKSTLNELKAEKL...HISGRMQRR....QQLEEQTVELSTEVQSLSREIKEAKEQI....FPLDATLEKLQQE   925
XP 008169432.1 DNA repair protein RAD50 Chrysemys picta bellii          VTGKIELNQKLKQDQQTQIQQLKSTVNELKAEKL...QISSNMQRR....QQLEDQTVELSTGVQSLYREIKEAKDQL....FPLEATLEKLQRE   925
XP 007443325.1, XP 007441819.1 DNA repair protein RAD50, partial Python bivittatus VTSKIELKQTLKQDQQNQVQYLRCTVNELKAEKL...QVSSCMQRQ....QQLEEQTVELTTEVQSLNREIKEAKEQL....SPLEATLGKLQQE   924
XP 012815225.1 DNA repair protein RAD50 Xenopus tropicalis              VSGKIELLRKRIQDQQEQVQQLKSTVNELTAEKL...HISSNLQRR....QQLEEQNVELTTELQCLSREIKEAKEQV....FPLESTLQKFQQE   925
XP 018418728.1 PREDICTED: DNA repair protein RAD50 Nanorana parkeri     VSGKIELLRKRIQDQQEQIQQLKSSANELRAEKL...QISSNLQRR....QQLEEQSVEMTTELQCLSREIREAREQV....FPLDNTMEKLQQE   925
XP 005167995.1 DNA repair protein RAD50 Danio rerio                     TSSKIELKRKLIQDQQEQIQALRSSVNEIRGEKL...QISSNMQKR....QQLEEQCVEFSTEIQTLHRDIRDAKEQA....SPLAATLEKLQQE   925
XP 030217475.1 DNA repair protein RAD50 Gadus morhua                    TSSKVELKRKLIQDQQERVQSLRSAVNETRGQKL...QLSSDMQQQ....QALTAQSVETTTEVQSLTREIRDAKEQL....TPISAMLEKLQQE   925
XP 007900169.1 PREDICTED: DNA repair protein RAD50 Callorhinchus milii  VSSRVELKRKLIQDQQELIQSLKSSLNEVRAEKL...QIASNMQRR....QQLEEQSVELATEIESLHRDIREAKEQL....SPLAAALEKLLKE   925
XP 032811404.1 DNA repair protein RAD50 Petromyzon marinus              VNNALELKRLALETDVERINELRSRVNSVREERL...RLAAGMQRI....QALRSVTAELEAETSDLKRQIQEAQEQV....LPLEGTLRKLQQE   951
XP 030837044.1 DNA repair protein RAD50 Strongylocentrotus purpuratus   VRSTLELKQRQRQEHVDNVNMMRISINSITSETL...RLSAKLQKR....EKLESEQAELSTNTRTFQREIEDSRQEQ....RPIKDKLQKLAQD   929
NP 001246461.1 rad50, isoform E Drosophila melanogaster                ERKELESAQNAVQQQMDALNRLREKKNSLKDRQI...HLREGLQSL....PQLKERLEKLNSFLTTVASEISELKAKI....QPLKLNLRAAIEE   932
XP 026298578.1 DNA repair protein RAD50 Apis mellifera                  IRENIERLQFEINMYNEKLHNNQQEQNTLHKEQL...KIQSDMQKL....KELKDNQEILYLKEISLGKSIDTLREKI....TLSETELNSGFDK   931
NP 001122999.1 Zinc-hook domain-containing protein Caenorhabditis elegans EYRKIVQEGEELQKCSEERNKLQSKLNELGTHRV...SLGEAAAQAGAFAEQLETKIKEIQECITAISQKRNEDLPDAQFKKDDLTRNVSSKEEE   926
XP 005102891.1 PREDICTED: DNA repair protein RAD50-like Aplysia californica VNRGLDHKRDKLSGHQDRLLRLRTRVHDLTGEKL...TIEGELQQK....TKLEERKVTLTSDNATYLRDIEEAKAQL....KPIETQIGKIAAE   935
NP 014149.1 MRX complex DNA-binding subunit Saccharomyces cerevisiae S288C LRKTISDLQMEKD...EKVRENSRMINLIKEKELTVSEIESSLTQK....QNIDDSIRSKRENINDIDSRVKELEARI....ISLKNKKDEAQSV   933
NP 001342968.1 DNA repair protein Rad50 Schizosaccharomyces pombe       LRKQIERTKLEETSFKKKIDDAVLANNE...QKLKLTKLNFQVNEL....EQLEKDINKSSEDCDLQKKKLLEVSSKQ....GSQAPFLNELESE   916
```

logo

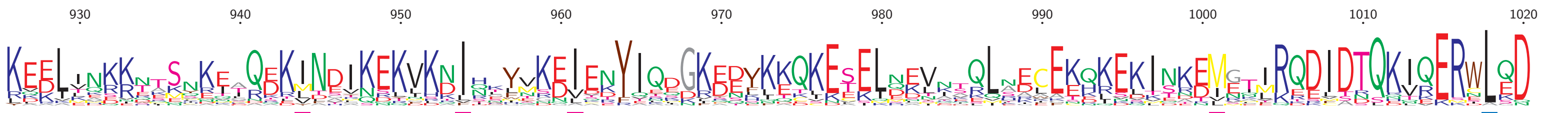

```
HsRAD50                                                                  KEELINKKNTSNKIAQDKLNDIKEKVKNIHGYMKDIENYIQDGKDDYKKQKETELNKVIAQLSECEKHKEKINEDMRLMRQDIDTQKIQERWLQD   1020
NP 033038.2 DNA repair protein RAD50 Mus musculus                        KEELIHRKHTSNKMAQDKINDIKEKVKNIHGYMKDIENYIQDGKDDYKKQKETELNGVAVQLNECEKHREKINKDMGTMRQDIDTQKIQERWLQD   1020
XP 003354322.2 DNA repair protein RAD50 Sus scrofa                       KEELINKKNTSSKIAQDKINDIKEKVKNIHGYVKDIENYIQDGKDEYKKQKETELNKVIVQLSECETHKEKVNKEMGIMRQDIDTQKIQERWLQD   1020
XP 003405070.1 DNA repair protein RAD50 Loxodonta africana               KEELINKKNKSNKIIQDKLNDIKEKVKNIQSYVKDIENYIQDGKDDYKKQKETELNKVITQLSECEKQKEKINKEMGIMRQDIDTQKIQERWLQD   1020
XP 028906603.1 DNA repair protein RAD50 Ornithorhynchus anatinus         KEELIDKKNTNSKIAQDKLNDIQEKIKNIHSHVKNIEDYIQDGKENYKQQKESELNKVATQINECEKQKEKINNEMGTMRQDIDTQKIQERWLQD   1020
XP 414645.3 DNA repair protein RAD50 isoform X2 Gallus gallus             KEDLINKRTASNKEIQEKMNAIKEKVKDINKYTKEIENYIQQGKEEYKKQKECELDEVNSQLVACEKQKEKISKEMEIIRQDIDTQKIQERWLED   1020
XP 029854070.1 DNA repair protein RAD50 Aquila chrysaetos chrysaetos     KEDLMNKRTASNKETQEKINGIKEKVKDINKYVKEIENYIQQGKEDYKKQKESELDEVNSQLAACEKQKEKISKEMETIRQDIDTQKIQERWLED   1020
XP 032606694.1 LOW QUALITY PROTEIN: DNA repair protein RAD50 Taeniopygia guttata KEELVNKRTASNKETQDKMNSINEKVKDIYKYVKEIENYIQQGKEDYKKQKESELDEVNSRLAACEKQKEKINKEMEMTRQDIDTQKIRERWLED   1020
XP 005147338.2 DNA repair protein RAD50 Melopsittacus undulatus          KEDLVNKRTTSNKETQDKMNGINEKVKDINKYVKEIENYIQKGRYDYKKQKESELNEVNSQLADWEKQKEKISKEMETIRQDIDTQKIQERWLQD   1020
XP 014453124.1 PREDICTED: DNA repair protein RAD50 Alligator mississippiensis REDLINKKNTSNKTMQDKINDTKEKVKTIHNYMKEIENYIQDGKEDYKKQKESELDEVKTQLADCENRKERINKEMGTIRQDIDTQKIQERWLED   1020
XP 008169432.1 DNA repair protein RAD50 Chrysemys picta bellii          RVDLINKKNTSNKVTQEKINEIKEKVKNIHDYVKEIEKYIQGGKEDYKKQKESELDEVKTQLADCEKQKEKISKEMGTIRQDIDTQKIQERWLED   1020
XP 007443325.1, XP 007441819.1 DNA repair protein RAD50, partial Python bivittatus KEELLNKKNTSYKITQDKINDIKEKVKNIRNYMKEIENYIQEGKEEYKQQKESELEKVTIQLADCEEKKEKINKEMGTIRQDIDTQKVQERWLED   1019
XP 012815225.1 DNA repair protein RAD50 Xenopus tropicalis              KQELLQRKELSYREAQEKVNDIKEKVKKINLFSKDIEKYIQDGKEDFKEQKECELQELIARLNECEKQKEKINREMVNIRQDIDTQKIQERCLQD   1020
XP 018418728.1 PREDICTED: DNA repair protein RAD50 Nanorana parkeri     KKEILQSKDSSYRAVQERLNDMKEKVKKISLYVRDIEKYISDGREEFKEQKETELQDLKTRLNDCEKHKEVINKDMMTIRQDIDTQKIQERCLQD   1020
XP 005167995.1 DNA repair protein RAD50 Danio rerio                     KQDLVERRRKKQEEGQEKINAIKEKMKNITLFEKEITKYIEEGKDSYKEQKETELQEMDKQLHEAEKQREKTNKDMGNIRQDIDTQKVQERWLQD   1020
XP 030217475.1 DNA repair protein RAD50 Gadus morhua                    REELVVRRRQKQDEGQEKMNTIKDKVKNISTLERDISRYTEEGKQEHKEQKESELHDTNGQLHEAEKHKEKITKEMGAVRQDIDTQKVQERWLQD   1020
XP 007900169.1 PREDICTED: DNA repair protein RAD50 Callorhinchus milii  KLDLMEKKTASCKEIQEKINDVKEKVKNIDWFAKEIQRYIQEKKDEFKEQKESELQETVTQLNEVEKQREKIQRDIEAFRKDIDTQKLEERWLQD   1020
XP 032811404.1 DNA repair protein RAD50 Petromyzon marinus              KAEITARKTREHNQEARRITDLQDKNKNIITYMKEVDTYINSGRDQLNKKKELEMQEVARELSGEEENQVQIGRDISGIRQEIDTQKVRERCLQD   1046
XP 030837044.1 DNA repair protein RAD50 Strongylocentrotus purpuratus   KVAITQKKESEIKEIREKIDEVQQQLRDIRAGTSNIAKYVTDGKESKLDDVEVKIMDLNEKLTKLGEDLSLMTATIEQLKDDISNQKVKEMELGN   1024
NP 001246461.1 rad50, isoform E Drosophila melanogaster                KERLKKSESEKLAQLNSKYNSYKSTDHDIQRLNKEAEDYAKLDLRNEIKKLDEIIMASKDKLRKLATEISLKTDELETIKTECSNQQTVERDLKD   1027
XP 026298578.1 DNA repair protein RAD50 Apis mellifera                  LEKKKKDNWEKQETDRKLITEGSRRLSDLQKMQDDVDSFIYRKIPESLECSESKIKSYEKLLNELFCKKNDIETTINKLKEEMTRQEVRKRELSD   1026
NP 001122999.1 Zinc-hook domain-containing protein Caenorhabditis elegans KKKAEMEVQMMKKELDQKIFHRKSLFKKVQE..GGLCERQLMDKENNIATLNASLEENQQRQKRFEEDL..........RSFDSSHQRESILKD   1008
XP 005102891.1 PREDICTED: DNA repair protein RAD50-like Aplysia californica KDKLTQQKEKMMEEARADVDVVKNKGLAIRELNGQVKTYQQSNKAQHLTECKEKQNKLRSSQERKEEERKDLEARVNQLREEVTRQDIKKRSLED   1030
NP 014149.1 MRX complex DNA-binding subunit Saccharomyces cerevisiae S288C LDKVKNERDIQVRNKQKTVADINRLIDRFQTIYNEVVDFEAKGFDELQTTIKE.LELNKAQMLELKEQLDLKSNEVNEEKRKLADSNNEEKNLKQ   1027
NP 001342968.1 DNA repair protein Rad50 Schizosaccharomyces pombe       YEKLEADIQEMAQKSRTEILEANEYLHQLNEWNSEL....RIDVSTKFKCIKEKKSNIGEEVRIIASKIESTDDNLRKLQERLADLRTRERNASD   1007
```

logo

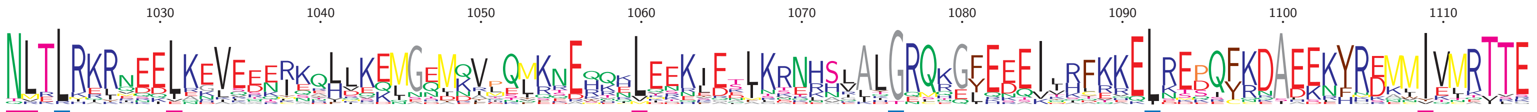

```
HsRAD50  NLTLRKRNEELKEVEEERKQHLKEMGQMQVLQMKSEHQKLEENIDNIKRNHNLALGRQKGYEEEIIHFKKELREPQFRDAEEKYREMMIVMRTTE  1115
NP 033038.2 DNA repair protein RAD50 Mus musculus  NLTLRKRRDELKEVEEERKQHLKEMGQMQVLQMKNEHQKLEENIDTIKRNHSLALGRQKGYEDEILHFKKELREPQFRDAEEKYREMMIVMRTTE  1115
XP 003354322.2 DNA repair protein RAD50 Sus scrofa  NLTLRKRNEELKEVEEERKQHLKEMGQMQVLQMKNEHQNLEEKIENIKRNHSLAIGRQKGYEEEIIHFKKELREPQFRDADEKYREMLIVMRTTE  1115
XP 003405070.1 DNA repair protein RAD50 Loxodonta africana  NLTLRKRNEELKEVEEERKQHLKEMGQMQVLQMKNEHQKLEEKIETIKRNHSLALGRQKGYEEEIIHFKKELREPQFRDAEEKYREMMIVMRTTE  1115
XP 028906603.1 DNA repair protein RAD50 Ornithorhynchus anatinus  NLTLRKRNEELKEVEEERKQLSKEMGQMQVLQLKTEHQKLEEKIETLKRNLSVALGRQKGYEEEIIHFKKELRDPQFRDAEEKHREMMIVMRTTE  1115
XP 414645.3 DNA repair protein RAD50 isoform X2 Gallus gallus  NLTLRERNKELKGVEDNIKELVKKMGEMKVPQLKNEQKHLEEKIEALKRNHHVALGRQRGFEEEIVRFKKELRESQFKDAEEKHREMMIVMRTTE  1115
XP 029854070.1 DNA repair protein RAD50 Aquila chrysaetos chrysaetos  NLTLRKRNEELKEVEDNIKQLVKEMGEMKVPQMKNDQKHLEEKIESLKRNHHVALGRQRGFEEEIVRFKKELRESQFKDAEEKYREMMIVMRTTE  1115
XP 032606694.1 LOW QUALITY PROTEIN: DNA repair protein RAD50 Taeniopygia guttata  NLTLRKRNEELKEVEDNIKQLMKEMGEMKVPQMKNEQKHLEEKIESLKRNHHVALGRQRGFEEEIVRFRKELREPQFKDAEEKYRDMMIVMRTTE  1115
XP 005147338.2 DNA repair protein RAD50 Melopsittacus undulatus  NLTLRKRNEELKDVEDNIKQLVREMGEMKVPEMKNEQKQLEEKIESLKRNHHVALGRQRGFEEEIVRFKKELREPQFKDAEEKYGDMMIVMRTTE  1115
XP 014453124.1 PREDICTED: DNA repair protein RAD50 Alligator mississippiensis  NLTLRKRNEELKGVEEEIKQHLKELGEMKAQQMKTEQHHLEEKIEELKRNHSLALGRQRGFEEEIIRFKKELRESQFKDAEEKYRDMMIVMRTTE  1115
XP 008169432.1 DNA repair protein RAD50 Chrysemys picta bellii  NLTLRKRNDDLKEVEEERKQHLKEMGEMKVPQLKNERQCLVEKLEDFKRNHSLALGRQHGFEEEIIRFKKELRESQFKDAEEKYREMMIVMRTTE  1115
XP 007443325.1, XP 007441819.1 DNA repair protein RAD50, partial Python bivittatus  NLTLRKRHEDLKEVEDNIKQHLKEMEEMQVPQLKRDQKHLEEKIEDLKRNHSLALGRQHGFEEEILRCKRELKEQQFKDAEEKYREMMIVMRTTE  1114
XP 012815225.1 DNA repair protein RAD50 Xenopus tropicalis  NLTLRKRIEELKRVEEERQQLLKEMGQMKVMQMKNEHQELENKSETLKTNHSLALGRQKGFEDEILRFKKELREPQYKDAEEKYRDKMIVMRTTE  1115
XP 018418728.1 PREDICTED: DNA repair protein RAD50 Nanorana parkeri  NLTLRKRMEELKKVQQEREHLLKEMGQIKAVQMRNEHQELENKIESLKTNHSLALGRQKGFEDEIMRFKKELREPQYKDAEEKHREMMIVMRTTE  1115
XP 005167995.1 DNA repair protein RAD50 Danio rerio  NLTLRKRVEELKEVSRKREALIKEMGNMQVLQLRNERREVERKLEDLKKNRSVALGRQKGYEDEILRFRKELNEDQYCRAEDLYRDKMIVMRTTE  1115
XP 030217475.1 DNA repair protein RAD50 Gadus morhua  NLTLRRRGEELKEVLSRREALLKDMGNMQVMQLRQERREVERKVEELKKTRSIAQGRQNGFEEEILHCRKELREDQYNKADERFRDKMIVMRTTE  1115
XP 007900169.1 PREDICTED: DNA repair protein RAD50 Callorhinchus milii  NLTLRKRVEELRGVEAKRQDLLQQIGEMQVMQLKNERRQLDEKLEQLKDNRSLAHGRQKGFEEEILRFRKELKEQQYKDAEEKFREMMITIRTTE  1115
XP 032811404.1 DNA repair protein RAD50 Petromyzon marinus  NLTLRRRQQELANVANNINRLLEKMGSMNVMQLKSEQERLIGHLEELKSKRSRALGRQQGLQDAKYKFEVQLKEKQFRDAAKEYMDNIIVMKTSE  1141
XP 030837044.1 DNA repair protein RAD50 Strongylocentrotus purpuratus  NMRLKKYQQEVEKKSGEIEKLQEKLGGYNLITIEREKTEMEKKKASLNREKNQLDGRSTELRKEVTRQERELESDSYKNAEKNYNDMMIKCRTTE  1119
NP 001246461.1 rad50, isoform E Drosophila melanogaster  NRELKQLEDKEAKLRESCQVLDKQLGNLDFHSVSKEKVNLTKQRDKATVRKGELLGQLGEIHSQVNKLQREIDEPRFKESLKNFRKANYEIEVTR  1122
XP 026298578.1 DNA repair protein RAD50 Apis mellifera  NVKLREIQEIINNLQEQYSNIKEKLNTINYSEIFDEWQNLQSREQTILRQKNIIKGNQEELERTVQQYVQELKKDIYKQAHKNYKSKCIELTVVE  1121
NP 001122999.1 Zinc-hook domain-containing protein Caenorhabditis elegans  QLTRMIIENKIKELKRTLATFDGQINEDRITEQKQAYNKLQNELRLIGNEEVKIYTQMQEYEKQKKIAEAKLSTKECQNAESNYRDAIIELAITK  1103
XP 005102891.1 PREDICTED: DNA repair protein RAD50-like Aplysia californica  TMTLHKKQEDLRGVEEKMGEVRQKLGDLDIKNLQRERTNLMDEEDKMREDLHRATGRQQGFKDQIKATKKELNSDICKDADKKYRDMVIQNRTLE  1125
NP 014149.1 MRX complex DNA-binding subunit Saccharomyces cerevisiae S288C  NLELIELKSQLQHIESEISRLDVQNAEAERDKYQEESLRLRTRFEKLSSENAGKLGEMKQLQNQIDSLTHQLRTD.YKDIEKNYHKEWVELQTRS  1121
NP 001342968.1 DNA repair protein Rad50 Schizosaccharomyces pombe  NLRLRALMRQLEEAVTQKNYLLSQQSHDDRESFRERMQILKSKYGALNAERAGLLGECKQLENSITKDKEELNME.FKDADERFRRQLIKTKTTG  1101
```

logo

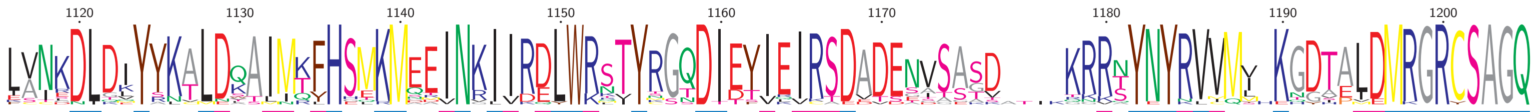

```
HsRAD50  LVNKDLDIYYKTLDQAIMKFHSMKMEEINKIIRDLWRSTYRGQDIEYIEIRSDADENVSASD....KRRNYNYRVVML.KGDTALDMRGRCSAGQ  1205
NP 033038.2 DNA repair protein RAD50 Mus musculus  LVNKDLDIYYKTLDQAIMKFHSMKMEEINKIIRDLWRSTYRGQDIEYIEIRSDADENVSASD....KRRNYNYRVVML.KGDTALDMRGRCSAGQ  1205
XP 003354322.2 DNA repair protein RAD50 Sus scrofa  LVNKDLDIYYKTLDQAIMKFHSMKMEEINKIIRDLWRSTYRGQDIEYIEIRSDADENVSASD....KRRNYNYRVVML.KGDTALDMRGRCSAGQ  1205
XP 003405070.1 DNA repair protein RAD50 Loxodonta africana  LVNKDLDIYYKTLDQAIMKFHSMKMEEINKIIRELWRNTYRGQDIEYIEIRSDADENVSASD....KRRNYNYRVVML.KGDTALDMRGRCSAGQ  1205
XP 028906603.1 DNA repair protein RAD50 Ornithorhynchus anatinus  LVNKDLDIYYKTLDQAIMKFHSMKMEEINKIIRDLWRTTYRGQDIEYIEIRSDADENVSASD....KRRSYNYRVVMI.KGDTALDMRGRCSAGQ  1205
XP 414645.3 DNA repair protein RAD50 isoform X2 Gallus gallus  LVNKDLDIYYKALDKAIMTFHSMKMQEINKIIRDLWRSTYRGQDIEYIEIRSDADENVSASD....KRRSYNYRVVMI.KGDTALDMRGRCSAGQ  1205
XP 029854070.1 DNA repair protein RAD50 Aquila chrysaetos chrysaetos  LVNKDLDLYYKALDKAIMTFHSMKMEEINKIIRDLWRSTYRGQDIEYIEIRSDADENVSASD....KRRSYNYRVVMI.KGDTALDMRGRCSAGQ  1205
XP 032606694.1 LOW QUALITY PROTEIN: DNA repair protein RAD50 Taeniopygia guttata  LVNKDLDLYYKALDKAIMTFHSMKMEEINKIIRDLWRSIYRGQDIEYIEIRSDADESVSATD....KRRNYNYRVVMV.KGDTALDMRGRCSAGQ  1205
XP 005147338.2 DNA repair protein RAD50 Melopsittacus undulatus  LVNKDLDLYYKALDKAIMTFHSMKMEAINKIIRELWRNTYRGQDIEYIEIRSDADENVSASD....KRRSYNYRVVMV.KGGTAMDMRGRCSAGQ  1205
XP 014453124.1 PREDICTED: DNA repair protein RAD50 Alligator mississippiensis  LVNKDLDIYYKALDKAIMTFHSMKMEEINKIIRDLWRSTYRGQDIEYIEIRSDADENVSASD....KRRSYNYRVVMI.KGDTALDMRGRCSAGQ  1205
XP 008169432.1 DNA repair protein RAD50 Chrysemys picta bellii  LVNRDLDMYYKALDKAIMTFHSMKMEEINKIIRDLWRSTYRGQDIEYVEIRSDADENVSASD....KRRNYNYRVVMI.KGDTALDMRGRCSAGQ  1205
XP 007443325.1, XP 007441819.1 DNA repair protein RAD50, partial Python bivittatus  LANKDLDIYYKALDQAIMTFHSMKMEEINKIIRDLWRSTYRGQDIEYIEIRSDADENISASD....KRRSYNYRVVMM.KGDTALDMRGRCSAGQ  1204
XP 012815225.1 DNA repair protein RAD50 Xenopus tropicalis  LAIKDLDIYYKTLDQAIMKFHSMKMEEINKIIRDLWRSTYRGQDIEYIEIRSDADEGVSAAD....KRRTYNYRVVMI.KGDTALDMRGRCSAGQ  1205
XP 018418728.1 PREDICTED: DNA repair protein RAD50 Nanorana parkeri  LANKDLDIYYKTLDQAIMKFHSMKMEEINKIIRDLWRSTYRGQDIEYIEIRSDADENVSA.D....KRRTYNYRVVMI.KGDAALDMRGRCSAGQ  1204
XP 005167995.1 DNA repair protein RAD50 Danio rerio  LANKDLDIYYKALDQTIMRFHSMKMEEINKIIRDLWRSTYRGQDIEYVEIRSDVDENASAGV....KRRTYNYRVVMV.KGDAALDMRGRCSAGQ  1205
XP 030217475.1 DNA repair protein RAD50 Gadus morhua  LVNKDLDIYYKALDQTIMKFHSMKMDEINKIIRDLWRSTYRGQDIEYVEIRSDVDENSSAGV....KRRTYNYRVVMM.KGDTALDMRGRCSAGQ  1205
XP 007900169.1 PREDICTED: DNA repair protein RAD50 Callorhinchus milii  LANRDLDIYYKALDQAIMKFHSMKMQEINKIIRDLWRSTYRGQDIEYIEIRSDADENVTAGD....KRRNYNYRVVMV.KGDTPLDMRGRYSAGQ  1205
XP 032811404.1 DNA repair protein RAD50 Petromyzon marinus  LACKDLDVYYKALDQAIMKFHSMKMQEINKIIRDLWRNTYRSQDIEYIEIRSDADESASASD....KRRNYNYRVVMV.KGDTPLDMRGRCSAGQ  1231
XP 030837044.1 DNA repair protein RAD50 Strongylocentrotus purpuratus  IANKDLDKYYNALNSAINTYHSQKMSEINKIVRDLWRMTYKGSDIDFIEICAD.DETGASTT....QRRQYNYRVVMV.KGDTALDMRGRCSAGQ  1208
NP 001246461.1 rad50, isoform E Drosophila melanogaster  LCIEDLGQYRLALEWALIQFHSEKMEMINRLIREYWRKIYRGNDIDYIQVKTDEVSSDASAD....RRKTYNYRVVQS.KNYSEIEMRGRCSAGQ  1212
XP 026298578.1 DNA repair protein RAD50 Apis mellifera  ESILNLKEYSKVLDTAMIQYHEERMATVNRIIKQLWKLVYTGTDTTSIEIRTDATEGIGGT......KRTYNYKLIQM.KHGHEIDMKGRCSAGQ  1209
NP 001122999.1 Zinc-hook domain-containing protein Caenorhabditis elegans  ESISDLTKYRNCLDASLIQFHSEKMGRVNGIIDDLWRKVYNSTDITTIRIRSDATSETSSK......KVAYEYNVMMVHETGTEVEMRGRCSAGQ  1192
XP 005102891.1 PREDICTED: DNA repair protein RAD50-like Aplysia californica  IANGDLQKYYKAMDLAIMRYHHTKMEEINVIVRELWKNTYMGNDIETIEVRSEDDDAATSGSATIKTRRNYNYRVVMI.KNGIPIDMRGRCSAGQ  1219
NP 014149.1 MRX complex DNA-binding subunit Saccharomyces cerevisiae S288C  FVTDDIDVYSKALDSAIMKYHGLKMQDINRIIDELWKRTYSGTDIDTIKIRSD...EVSSTV....KGKSYNYRVVMY.KQDVELDMRGRCSAGQ  1208
NP 001342968.1 DNA repair protein Rad50 Schizosaccharomyces pombe  KANEDLGKYAKALDVAIMQLHSMKMNEINRIVDELWKQTYCGTDIDTILIRSD.....SEGK....GNRTYNYRVCMV.KGDAELDMRGRCSAGQ  1186
```

logo

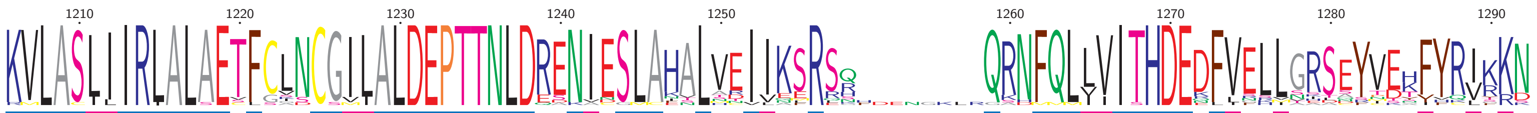

```
HsRAD50  KVLASLIIRLALAETFCLNCGIIALDEPTTNLDRENIESLAHALVEIIKSRSQ........QRNFQLLVITHDEDFVELLGRSEYVEKFYRIKKN  1292
NP 033038.2 DNA repair protein RAD50 Mus musculus  KVLASLIIRLALAETFCLNCGILALDEPTTNLDRENIESLAHALVEIIKSRSQ........QRNFQLLVITHDEDFVELLGRSEYVEKFYRVKKN  1292
XP 003354322.2 DNA repair protein RAD50 Sus scrofa  KVLASLIIRLALAETFCLNCGILALDEPTTNLDRENIESLAHALVEIIKSRSQ........QRNFQLLVITHDEDFVELLGRSEYVEKFYRIKKN  1292
XP 003405070.1 DNA repair protein RAD50 Loxodonta africana  KVLASLIIRLALAETFCLNCGILALDEPTTNLDRENIESLAHALVEIIKSRSQ........QRNFQLLVITHDEDFVELLGRSEYMEKFYRIKKN  1292
XP 028906603.1 DNA repair protein RAD50 Ornithorhynchus anatinus  KVLASLIIRLALAETFCLNCGILALDEPTTNLDRENIESLAHALVEIIKSRSQ........QRNFQLLVITHDEDFVELLGRSEYVEKFYRIKKN  1292
XP 414645.3 DNA repair protein RAD50 isoform X2 Gallus gallus  KVLASLIIRLALAETFCLNCGILALDEPTTNLDRENIESLAHALVEIIKSRSR........QRNFQLLVITHDEDFVELLGRSEYVETFYRIRKN  1292
XP 029854070.1 DNA repair protein RAD50 Aquila chrysaetos chrysaetos  KVLASLIIRLALAETFCLNCGILALDEPTTNLDRENIESLAHALMEIIKSRSQ........QRNFQLLVITHDEDFVELLGRSEYVETFYRIKKN  1292
XP 032606694.1 LOW QUALITY PROTEIN: DNA repair protein RAD50 Taeniopygia guttata  KVLASLIIRLALAETFCINCGILALDEPTTNLDRENIESLAHALVEIIKSRLH........QRNFQLLVITHDEDFVELLGRSEYVETFYRIRKN  1292
XP 005147338.2 DNA repair protein RAD50 Melopsittacus undulatus  KVLASLIIRLALAETFCLNCGILALDEPTTNLDRENIESLAHALTEIIKSRSQ........QRNFQLLVITHDEDFVELLVRSEYVEAFYRIRKS  1292
XP 014453124.1 PREDICTED: DNA repair protein RAD50 Alligator mississippiensis  KVLASLIIRLALAETFCLNCGILALDEPTTNLDRENIESLAHALVEIIKSRSQ........QRNFQLLVITHDEDFVELLGRSEYVETFYRIKKN  1292
XP 008169432.1 DNA repair protein RAD50 Chrysemys picta bellii  KVLASLIIRLALAETFCLNCGILALDEPTTNLDRENIESLAHALVEIIKSRSQ........QRNFQLLVITHDEDFVELLGRSEYVEKFYRIKKN  1292
XP 007443325.1, XP 007441819.1 DNA repair protein RAD50, partial Python bivittatus  KVLASLIIRLALAESFCLNCGILALDEPTTNLDRENIESLAHALVEIIKSRSH........QRNFQLLVITHDEDFVELLSRSEYVEKFYRIKKN  1291
XP 012815225.1 DNA repair protein RAD50 Xenopus tropicalis  KVLASLIIRLALAETFCLNCGILALDEPTTNLDRENIESLAHALVEIIKSRSR........QRNFQLVVITHDEDFVELLGRSEYVEHFYRIKKN  1292
XP 018418728.1 PREDICTED: DNA repair protein RAD50 Nanorana parkeri  KVLASLIIRLALAETFCLNCGILALDEPTTNLDRENIESLAHALVEIIKSRSR........QRNFQLLVITHDEDFVELLGRSEYVEHFYRIKKN  1291
XP 005167995.1 DNA repair protein RAD50 Danio rerio  KVLASLIIRLALAETFCLNCGILALDEPTTNLDRENIESLAHALVEIIKSRSR........QRNFQLLVITHDEDFVELLGRSNYVEHFYRIRKN  1292
XP 030217475.1 DNA repair protein RAD50 Gadus morhua  KVLASLIIRLALAETFCLNCGILALDEPTTNLDRENIESLAHALVEIIKSRSR........QRNFQLLIITHDEDFVELLGRSSYVEHFYRIRKN  1292
XP 007900169.1 PREDICTED: DNA repair protein RAD50 Callorhinchus milii  KVLASLIIRLALAETFCLNCGILALDEPTTNLDRENIESLAHALVEIIKSRSN........QRNFQLVVITHDEDFVELLSRSQYVEHFYRVKKN  1292
XP 032811404.1 DNA repair protein RAD50 Petromyzon marinus  KVLASLIIRLALAETFCLNCGILALDEPTTNLDRENIESLAHALVEIIKSRSR........QRNFQLLVITHDEDFVELLGRSEYVDHFYRVRKN  1318
XP 030837044.1 DNA repair protein RAD50 Strongylocentrotus purpuratus  KVLASLLIRLALAETFCLSCGILALDEPTTNLDRDNIESLAHALVDILKSREN........QRNFQLLIITHDEEFVELLGHSDYTDNYYRVTKN  1295
NP 001246461.1 rad50, isoform E Drosophila melanogaster  RVLASLIIRLALAETFSSNCGVLALDEPTTNLDRANINSLCEALNCIVEERQS........QSNFMLIIITHDENFVSSLGK...ITSYHRVFRN  1296
XP 026298578.1 DNA repair protein RAD50 Apis mellifera  KVLASIIIRLALAETFCKDCGILALDEPTTNLDQENADSLANTLATVVKLRSQH.......QKNFQLIVISHDEKFLFKLAELSSNKGFYQLYRK  1297
NP 001122999.1 Zinc-hook domain-containing protein Caenorhabditis elegans  KMLASLLIRIALAEVFGGSCSMIALDEPTTNLDESKVEGMAIVLADIIAERRGFDENGKLRGRDMQMVVITHDERLVNRITISCRPEYIYCLGKD  1287
XP 005102891.1 PREDICTED: DNA repair protein RAD50-like Aplysia californica  KVLASLIIRLALAETFCVNCGILALDEPTTNLDRSNIESLAFALVRIIQERSS........QRHFQLVVITHDEDFVELLGRTEYVDHFVQVSKN  1306
NP 014149.1 MRX complex DNA-binding subunit Saccharomyces cerevisiae S288C  KVLASIIIRLALSETFGANCGVIALDEPTTNLDEENIESLAKSLHNIINMRRH........QKNFQLIVITHDEKFLGHMNAAAFTDHFFKVKRD  1295
NP 001342968.1 DNA repair protein Rad50 Schizosaccharomyces pombe  KVLACIIIRLALAECLGVNCGILALDEPTTNLDEENICSLAKNLSRIVEFRRK........QANFQLIVITHDEQFIRLVNSDAYCSYYYRVKRD  1273
```

logo

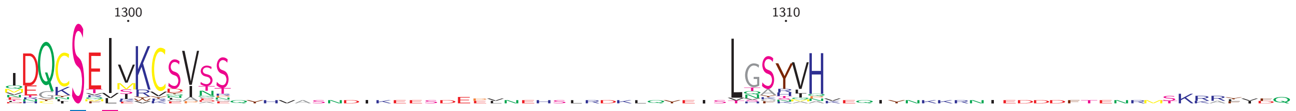

```
HsRAD50  IDQCSEIVKCSVSS...............................LGFNVH.............................  1312
NP 033038.2 DNA repair protein RAD50 Mus musculus  MDQCSEIVKCSISS...............................LGSYVH.............................  1312
XP 003354322.2 DNA repair protein RAD50 Sus scrofa  IDQCSEIVKCSVSS...............................LGSYVH.............................  1312
XP 003405070.1 DNA repair protein RAD50 Loxodonta africana  MDQCSEIVKCSVSS...............................LGSYVH.............................  1312
XP 028906603.1 DNA repair protein RAD50 Ornithorhynchus anatinus  IDQCSEIVKCSVSS...............................LGSYVH.............................  1312
XP 414645.3 DNA repair protein RAD50 isoform X2 Gallus gallus  IDQCSEIMKCSVSS...............................LGSYVH.............................  1312
XP 029854070.1 DNA repair protein RAD50 Aquila chrysaetos chrysaetos  IDQCSEIMKCSVSS...............................LGSYIH.............................  1312
XP 032606694.1 LOW QUALITY PROTEIN: DNA repair protein RAD50 Taeniopygia guttata  IDQCSEIMKCSVSS...............................LGSYVH.............................  1312
XP 005147338.2 DNA repair protein RAD50 Melopsittacus undulatus  VDQCSEIIKCNASS...............................LGSYVH.............................  1312
XP 014453124.1 PREDICTED: DNA repair protein RAD50 Alligator mississippiensis  IDQCSEIVKCSVSS...............................LGSYVH.............................  1312
XP 008169432.1 DNA repair protein RAD50 Chrysemys picta bellii  IDQCSEIVKCSVSS...............................LGSYAH.............................  1312
XP 007443325.1, XP 007441819.1 DNA repair protein RAD50, partial Python bivittatus  LDQCSEIVKCSVTT...............................LGSYVH.............................  1311
XP 012815225.1 DNA repair protein RAD50 Xenopus tropicalis  IDQCSEIMRCSVNS...............................LTSYVH.............................  1312
XP 018418728.1 PREDICTED: DNA repair protein RAD50 Nanorana parkeri  MDQCSEIVKCGVSS...............................LGSYMH.............................  1311
XP 005167995.1 DNA repair protein RAD50 Danio rerio  QDQCSEISKCSINS...............................LNSYLH.............................  1312
XP 030217475.1 DNA repair protein RAD50 Gadus morhua  QEQNSEIIKCSITS...............................LSSHLH.............................  1312
XP 007900169.1 PREDICTED: DNA repair protein RAD50 Callorhinchus milii  IEQCSEIIRCSVTS...............................LNAHVQ.............................  1312
XP 032811404.1 DNA repair protein RAD50 Petromyzon marinus  NEQCSEIVKCSVST...............................LQASVA.............................  1338
XP 030837044.1 DNA repair protein RAD50 Strongylocentrotus purpuratus  NDYKSTVVKYDIAT...............................LQATQPV............................  1316
NP 001246461.1 rad50, isoform E Drosophila melanogaster  EECKSVIRRVEAGP..........................................................SKKALIDQ  1318
XP 026298578.1 DNA repair protein RAD50 Apis mellifera  QTGYSTVKYCLVENQYHVASNDIKEESDEEVNEHSLRDKLQYEISLGGKIHKEQIYNKKRNIEDDDFTENRMSKRRYVFQ  1377
NP 001122999.1 Zinc-hook domain-containing protein Caenorhabditis elegans  EHGISFLSKRYPDG...............................TVKRVN.....................TKRRF...  1312
XP 005102891.1 PREDICTED: DNA repair protein RAD50-like Aplysia californica  VEGLSRLSVKQVQE...............................LHSR...............................  1324
NP 014149.1 MRX complex DNA-binding subunit Saccharomyces cerevisiae S288C  DRQKSQIEWVDINR...............................VTY................................  1312
NP 001342968.1 DNA repair protein Rad50 Schizosaccharomyces pombe  TNQKSMIVK...................EPL.................................................  1285
```

X non-conserved
X similar
X ≥98% conserved

C. NBN

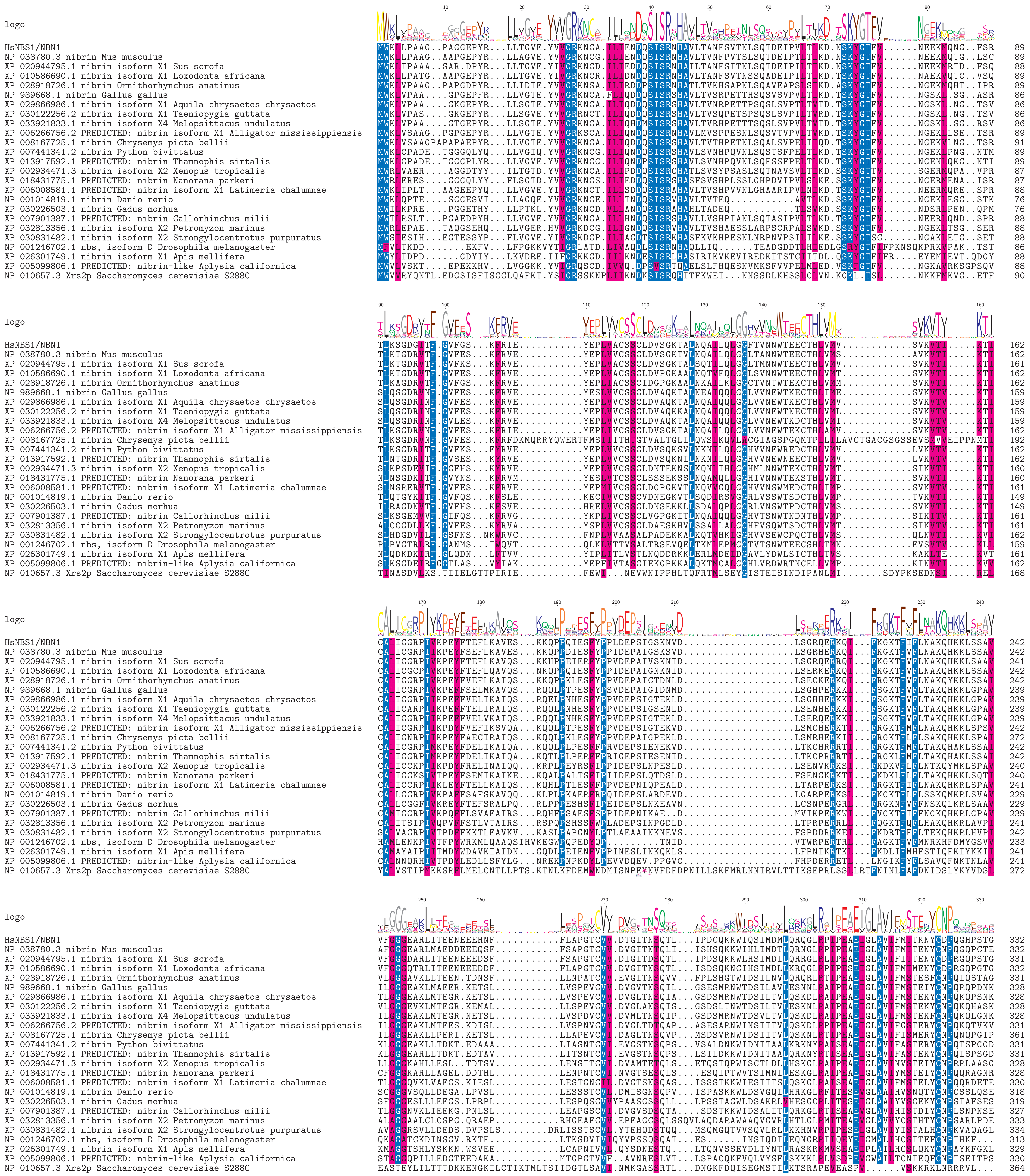
logo
HsNBS1/NBN1
NP 038780.3 nibrin Mus musculus
XP 020944795.1 nibrin isoform X1 Sus scrofa
XP 010586690.1 nibrin isoform X1 Loxodonta africana
XP 028918726.1 nibrin Ornithorhynchus anatinus
NP 989668.1 nibrin Gallus gallus
XP 029866986.1 nibrin isoform X1 Aquila chrysaetos chrysaetos
XP 030122256.2 nibrin isoform X1 Taeniopygia guttata
XP 033921833.1 nibrin isoform X4 Melopsittacus undulatus
XP 006266756.2 PREDICTED: nibrin isoform X1 Alligator mississippiensis
XP 008167725.1 nibrin Chrysemys picta bellii
XP 007441341.2 nibrin Python bivittatus
XP 013917592.1 PREDICTED: nibrin Thamnophis sirtalis
XP 002934471.3 nibrin isoform X2 Xenopus tropicalis
XP 018431775.1 PREDICTED: nibrin Nanorana parkeri
XP 006008581.1 PREDICTED: nibrin isoform X1 Latimeria chalumnae
NP 001014819.1 nibrin Danio rerio
XP 030226503.1 nibrin Gadus morhua
XP 007901387.1 PREDICTED: nibrin Callorhinchus milii
XP 032813356.1 nibrin isoform X2 Petromyzon marinus
XP 030831482.1 nibrin isoform X2 Strongylocentrotus purpuratus
NP 001246702.1 nbs, isoform D Drosophila melanogaster
XP 026301749.1 nibrin isoform X1 Apis mellifera
XP 005099806.1 PREDICTED: nibrin-like Aplysia californica
NP 010657.3 Xrs2p Saccharomyces cerevisiae S288C

logo

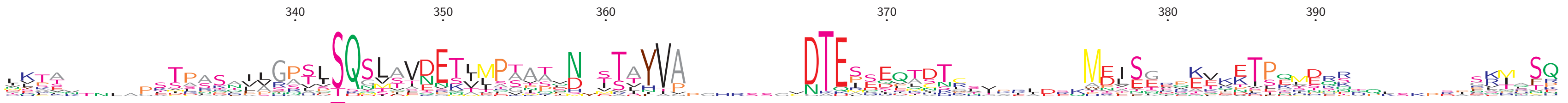

```
HsNBS1/NBN1                                                      LKTT.......TP.....GPSLSQGVSVDEKLMPSAPVN.TTTYVA........DTES.EQADT.........WDLSERPKEIKVSKMEQK........FRMLSQ   398
NP 038780.3 nibrin Mus musculus                                  LKTT.......TP.....GPSLSQVLSANGKIIPSAPVN.MTTYVA........DTES.EPADTC........MPLSERPEEVKIPGLEQS........SRKLSQ   399
XP 020944795.1 nibrin isoform X1 Sus scrofa                      LK.S.......TP.....GPSLSQGLSANENLIRGAPVN.TATYVA........DTES.EQADTC........MDLSERPKEVKIFEMDQN........LRIPSQ   397
XP 010586690.1 nibrin isoform X1 Loxodonta africana              LKPT.......TP.....GPSLSQGLSFSEKLMPTVPVN.TTTYVP........DTES.EQANTC........MDFSGSPKEIKISRMEQK........FRMLSP   399
XP 028918726.1 nibrin Ornithorhynchus anatinus                   LRSG.......IP.....RPGVSQSLFVDETLMPGATMN.VTAYVA........DTES.EQADTC........MDSSEK.QEDETLKIETR........GRMQSQ   397
NP 989668.1 nibrin Gallus gallus                                 AVTA.....STASK..VRPVSSQSSTVDETIMPTAAADYSTLNVA........DTEIEEQTC..........MEIE.....RTTSQTTRR........EKVAFQ   394
XP 029866986.1 nibrin isoform X1 Aquila chrysaetos chrysaetos   ALTK.....STPASAAAGRAISQSLAVDETIMLTAADH.STVHVA........DTEIEEQTC..........MEIE......DTPQMDGR........GQIAFQ   394
XP 030122256.2 nibrin isoform X1 Taeniopygia guttata            GLAAV....PESSASAVVGRAISQSLAVNETIMPTAADS.STLYIA........DTE..EQTC..........MEIE......NTSQVHRQ........RKMAFH   394
XP 033921833.1 nibrin isoform X4 Melopsittacus undulatus        VVTE.....STCASAVVGQHVSQSLVVDETLIAAASND.STAYVA........DTEIEEQTC..........MEIE......NTPQMDRQ........GKMAFQ   394
XP 006266756.2 PREDICTED: nibrin isoform X1 Alligator mississippiensis  IKPA......TPAANLLGSSISQSLIVEETV.PTATNN.DSVYVA........DTEPEDLTDSR........MEI.......DKTQMNRK........GKTNVQ   396
XP 008167725.1 nibrin Chrysemys picta bellii                     KQPV......SPGSAILHPTLSQSSTVDESIMPVTTSD..RAYVA........DTEPEEPC...........MEISGE.KTEKTPKMDRR........DKMYSQ   429
XP 007441341.2 nibrin Python bivittatus                         IKTA......TAASHVLGQSPSQSVTIKETAVPSNQDD.VSVYVA........DTEMDHLMDTLL.......VELSEEKTKKET.............PVIQSQ   399
XP 013917592.1 PREDICTED: nibrin Thamnophis sirtalis             IKTA......TLASQL...SQSQSVAVEETTMPSNQED.ISAFVA........DTEMDEVMDTLL.......MELSQDEKK................PVTQSQ   393
XP 002934471.3 nibrin isoform X2 Xenopus tropicalis             NEAE.....TSKRTQVLSSTLSQGMTVDETVLPAPALN.MTAYVA........DTEPQDQTDTG........MNISGVREVKETPGSSSS...........HSR   399
XP 018431775.1 PREDICTED: nibrin Nanorana parkeri                NEKE.....KSARSNIVGSSFSSSMAVNETVLPVATFN.TTAYVA........DTEPADKTHNW........MDISGAREVKETPKSSHS............TK   398
XP 006008581.1 PREDICTED: nibrin isoform X1 Latimeria chalumnae  TYTA..........TIPGPTLSQSLAIDETTMPAATLN.ITAYVP........DTEPSQELDTR........MEVSGLQEVKETPEKEQR........SRMRSR   399
NP 001014819.1 nibrin Danio rerio                                SVKT.........NPVFASATLSQSTAVDETALAAPSQN.ITAYVV........NTEISQDQSR.........MVTSGISAVGETPEKTNPTQK.....ASTTNK   391
XP 030226503.1 nibrin Gadus morhua                               DSVQ.....SVRPRLPPYASLSQNVAVEETVLQASSIN.ITAYVG........NTEASQGLGTKST......ARIETVGETQKTPQHSRTHPLSKPRPSAQGAG   403
XP 007901387.1 PREDICTED: nibrin Callorhinchus milii             MHFS.........SKTISRPTLSQQMDVDNMTEPGISQH..TSYVP........DTQR...TNTR........MNVNTTVEVKETPETDEE........SKIVSQ   394
XP 032813356.1 nibrin isoform X2 Petromyzon marinus             VSCVNTNLAGAMLSQNLAGATLSQNLALEPS..EGVAPD.STLYAA........DTQASLILARCHV......MSASGSQSQRVDETLDKR........SLIPTE   413
XP 030831482.1 nibrin isoform X2 Strongylocentrotus purpuratus  DLLS..............AVPSQSLATQNVLAS................DTESLSQKIHGVGGKLDRKATATAKKETAPTPNLPKQ........NLTSTP   396
NP 001246702.1 nbs, isoform D Drosophila melanogaster           .....................ISDSLPTTESVTSSMAFN.SSIIVP........NTERHSAQSNA........TPISELV.VPESIECEMEQ.....DASKPHSE   374
XP 026301749.1 nibrin isoform X1 Apis mellifera                LKRLK....PKCDSSKILN.LDTQDIVSNVKILPKIISN.VSI..........NTDLSIEKSKHTIKII...PDSYNDLKSQELLSVDFNKQ....SIKEIKCN   410
XP 005099806.1 PREDICTED: nibrin-like Aplysia californica       MYSA...............VATQMTQCDSQMMTTTTQM.EVDHTAPGHRSSGVDKKSSASSLNRSVPRLESQMDEGSEQKPVTPPPRNSSA.......RAGRSR   411
NP 010657.3 Xrs2p Saccharomyces cerevisiae S288C               .........PLDSLDFFAGGLSTKTLSENRSLTDAKRLN..............CGAESKTVISSPNIAEADEKHAPFLQNALKPTEDIGKK........SGHSSP   438
```

logo

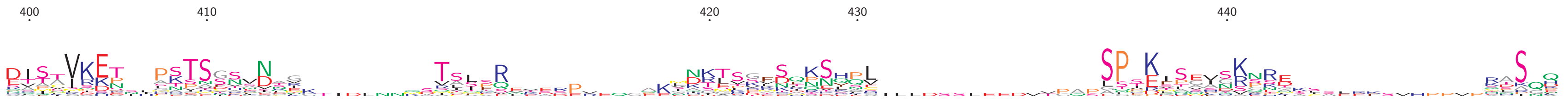

```
HsNBS1/NBN1                                                      DAPTVKES..CKTSSNNNSM.........VS...............NTLAKMRIPNYQL...............SPTKLPSINKSKD............RASQQ   449
NP 038780.3 nibrin Mus musculus                                  ETFNIKEA..PKPSSKANNV.........AS...............DTLVRGKTPSYQL...............SPMKFPVANKNKD............WTS.Q   449
XP 020944795.1 nibrin isoform X1 Sus scrofa                      EISAMKEY..HKISSHRSNI.........VS...............DTLVKMKIPNYQL...............SPTKFPGVNKNSD............QPSQH   448
XP 010586690.1 nibrin isoform X1 Loxodonta africana              ETSAVKEP..PKTSCNSDNI.........VS...............NTLVKVKVPDYQL...............SPTKFTGISKGRD............RVSQQ   450
XP 028918726.1 nibrin Ornithorhynchus anatinus                   DAPTVKET..PNTSCVSAGI.........TS...............QKMSRLSDQESQL...............SPPKL.....NRM............RNI.Q   442
NP 989668.1 nibrin Gallus gallus                                 Q.AAVREN..PSTSGTVNAG.........MLISR...........VNRTSGFGQKNHPH...............SPSKILEVDKPRE............CTP.R   447
XP 029866986.1 nibrin isoform X1 Aquila chrysaetos chrysaetos   DITAVKKN..LSTSGAINAG.........TSISR...........VNRTSGFSQKSHPV...............SPSEISEAGKPSE............HAS.R   448
XP 030122256.2 nibrin isoform X1 Taeniopygia guttata            DTTAIKKN..SGTSGSVNAG.........TSIPR...........GNRTSGFSQRSQPV...............SPSKISEAGKSHG............SAS.H   448
XP 033921833.1 nibrin isoform X4 Melopsittacus undulatus        DIATIKKN..PSTSGSLNAG.........TSTCR...........MNRTSGFSQKSHPV...............SPSKSSEAVKPRE............CAS.H   448
XP 006266756.2 PREDICTED: nibrin isoform X1 Alligator mississippiensis  DITTVKET..PNTSGNANVG.........ILFSR...........IDKMPEFDQKSLSL...............SPFKTSEVGKKRE............RAS.Q   450
XP 008167725.1 nibrin Chrysemys picta bellii                     NITSVKET..PSTSGSVNTG.........TVLPR...........MHRTLVVDQKSQPL...............SPSKILGVNRNRE............RAS.Q   483
XP 007441341.2 nibrin Python bivittatus                         DIITVKET..PAEAGNVNRE.........RILPE...........LKKTRGADHTSLAC...............SSTEISGHSRNRA............RDS.Q   453
XP 013917592.1 PREDICTED: nibrin Thamnophis sirtalis             DIMTVRET..PEGTGSVNRE.........RILPQ...........LKKTHGADFASLAC...............LSSEISSHSRNRE............EDS..   446
XP 002934471.3 nibrin isoform X2 Xenopus tropicalis             SRSCIEKE..PKSGSSNDLR.........QALFQ...........GDKTETRNKNNHPL...............LPIKPSARDVGKT............SQK.L   453
XP 018431775.1 PREDICTED: nibrin Nanorana parkeri                SGMCVDRD..LKSSSNGDCR.........TTLFQ............EVSKPTEKKSQLL...............LSIEKPASHKE..............KTSQE   450
XP 006008581.1 PREDICTED: nibrin isoform X1 Latimeria chalumnae  EISVVKET..PSTNSAEVLV.........TALRSEKERP.....ENSLTSKSASKEQL...............STRETSELGRSNE............GSS.Q   458
NP 001014819.1 nibrin Danio rerio                                PLSLGQEP..SSTRIVQETV.......MSSESFSVVESE....QKMKKGSVVSARGRVE...............GPVKQKAPSSGNTTL..........KHS.P   456
XP 030226503.1 nibrin Gadus morhua                               RVSAVADT..MSASFHTVEN.........TGSQQQEAGPREQGAKNAGVLPSNQKNHIG..........VGASSQKRSSQKQKPPA............QLS.P   472
XP 007901387.1 PREDICTED: nibrin Callorhinchus milii             DISVVIHP..ATSMFSPDQR.......................PKSKKEKSRSFNTGNE...............EPGEMAMFSSVRN............KDKNE   446
XP 032813356.1 nibrin isoform X2 Petromyzon marinus             ATSMSRDP..GRPEVRVGTTG.......ATAAAVLRDD.V...EEAADGSSLSTKSQPR...............GPCDSTDSAR...............KPA.A   473
XP 030831482.1 nibrin isoform X2 Strongylocentrotus purpuratus  KVSKVSETIIATPSRTANQ..........TASSR....P.....LDGSPEFSLQEK..................APIKME...................TGRGD   444
NP 001246702.1 nbs, isoform D Drosophila melanogaster           DQASLRKRSHASTVDSSDEEK......KSTLSKR.........AKSDIATKLTMKSKNAILLDSSLEEDVTPAPAPAPVQRVTRQSKAIAEEKSVHPPVPAAS.K   463
XP 026301749.1 nibrin isoform X1 Apis mellifera                ESQCIKET..KNTSDDKIFMKTIDLNNQDTCMTR..........IISESSEDFNSQES...............SNEDFNNISVRELKC..............N   472
XP 005099806.1 PREDICTED: nibrin-like Aplysia californica       RVSPKPDS..EATANPVDSK.........TPQERETSRP.....AGSESDKNDNVVSV...............SGVRDSVVSASEGKSSLRF.......RLSGD   477
NP 010657.3 Xrs2p Saccharomyces cerevisiae S288C               GAIIVSSPNLGTVNTSEDSL...........................DKSLQSHKL...........PQPSLPEVAGIGSQTISS..........NSA.D   489
```

logo

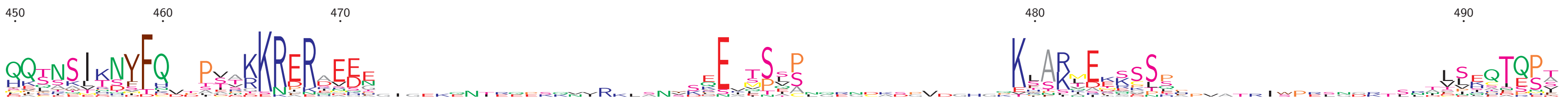

```
HsNBS1/NBN1                                                      QQTNSIRNYFQ..PSTKKRERDEEN.....................QE.MSSC.............KSARIETSCS..................LLEQTQPA   496
NP 038780.3 nibrin Mus musculus                                  QQQNSIKNYFQ..PCTRKRERDEDN.....................PE.LSSC.............KSSRMELSCS..................LLEQTQPA   496
XP 020944795.1 nibrin isoform X1 Sus scrofa                      QQTNSIKNYFH..PSPKKRERDEEN.....................QE.MSLS.............KSARTEMS.....................LEQTQPT   492
XP 010586690.1 nibrin isoform X1 Loxodonta africana              QQTNSIKNYFQ..PSTKKRGRGEEN.....................QE.MSSS.............KSARIEMSCS..................LLEQTQPA   497
XP 028918726.1 nibrin Ornithorhynchus anatinus                   QQTYSIKNYFQ..PTTKKRERDEEE.....................QE.MPAS.............KSAKTEELSSS.................LLAQTQPT   490
NP 989668.1 nibrin Gallus gallus                                 QQSNSITNYFH...VARKRERAEEG.....................EE.TSLS.............KQAKLEKKPLP.................VSECTESS   494
XP 029866986.1 nibrin isoform X1 Aquila chrysaetos chrysaetos   HQSNSITNYFQ...VARKRERAEEG.....................EE.TSLP.............KLAKLEERSSP.................VSKCIEPT   495
XP 030122256.2 nibrin isoform X1 Taeniopygia guttata            HQSNSITNYFQ...VSRKRERAEE......................EE.TSVP.............KLAKLEERSSP.................LPKCTEST   494
XP 033921833.1 nibrin isoform X4 Melopsittacus undulatus        HQYNSILNYFQ...VAGKRERAEEG.....................EE.TSVA.............KLAKLEERSLP.................VPKFTEPA   495
XP 006266756.2 PREDICTED: nibrin isoform X1 Alligator mississippiensis  QQLNSIKNYFQ..TATKKRERKEEE.....................EEVSSEP.............KLAKLEDKSLP.................SSSHTQPI   499
XP 008167725.1 nibrin Chrysemys picta bellii                     QESNSIKHYFQ..AAPKKRERIEEG......................E.TSVP.............KLAKMEEKSSH.................LSSQTQPT   530
XP 007441341.2 nibrin Python bivittatus                         QQLNSIKNYFQ..ITAKKRERSED......................VE.TPLS.............KHAKMEKKSSQ.................VSHQTPSV   500
XP 013917592.1 PREDICTED: nibrin Thamnophis sirtalis             QQLNSIKNYFQ..TTVKKRERNEE......................LE.TPLS.............KHAKMDKKSSQ.................VSHQTPSV   493
XP 002934471.3 nibrin isoform X2 Xenopus tropicalis             QPTKKIDSYFQ..SLAKKRDRDEDE.....................KE.VSSA.............KLARVEPVSSQ................NTEEQFRPI   502
XP 018431775.1 PREDICTED: nibrin Nanorana parkeri                MVPSKMPDYFQ..PTAKKRDREVDE.....................GE.QSTA.............KTSRVETGVSL.................GSNAAENI   498
XP 006008581.1 PREDICTED: nibrin isoform X1 Latimeria chalumnae  SQSSWLSGDFL..LVRKKRERSVDE.....................ES.SSQA.............KYTRTDRE.......................ETQSF   500
NP 001014819.1 nibrin Danio rerio                                QKQTALTSFFQ..PSSKKRPRESSA.....................SSVQPEP.............KFFKKDIKDNE...............DDIQQSSSV   506
XP 030226503.1 nibrin Gadus morhua                               QKQAPLTNFFQ..PVNKKRPRADEF....................SAE.ESEP.............KRALQSGASSS........................S   514
XP 007901387.1 PREDICTED: nibrin Callorhinchus milii             PQSSSIHDFFQ..PIRRKRERQGDE........................STQS.............KFARTEDSKPS.................LIHQTSMS   492
XP 032813356.1 nibrin isoform X2 Petromyzon marinus             HAKATITSYFQ..PSSRKRARHAGT....................DDEGSPVP............PKVSRAAEQPPTL....IWPESGDRTSRTCSLPTQPY   537
XP 030831482.1 nibrin isoform X2 Strongylocentrotus purpuratus  DKEEQVETSTR..PEVKKKDEKEDEGIGEKENTREEKQVVR....VKKEALSQPNGRGDADGVDGHGQKPASAEVSESL.................EVISEQPS   525
NP 001246702.1 nbs, isoform D Drosophila melanogaster           HITRKTKQVFCVDSSDEENENARKP.....................KE.TPAP.............TIPSMAKKKTEAPVATRISPRLNGK.SLATNITNQPA   530
XP 026301749.1 nibrin isoform X1 Apis mellifera                EQCIEEKDIFNDTPIIDKNIKIMQS....................KQNIVNIP.............ETNRNISESSSF.............LNILSKSTQHF   528
XP 005099806.1 PREDICTED: nibrin-like Aplysia californica       LFSSSTGNSTKVPPDEGSREKAVSE..............R..ANSSEEKETIS.............VFARQRKNGSKP...............TLVRRDSSD   535
NP 010657.3 Xrs2p Saccharomyces cerevisiae S288C               YETAAVNSMDD..AEVTKNFRVNHH....QNIEQPSKNIRKLSNYSRE.ISSPLQENCKSPV....KELSIKEKSGTPH...............AFVEAIQET   566
```

logo

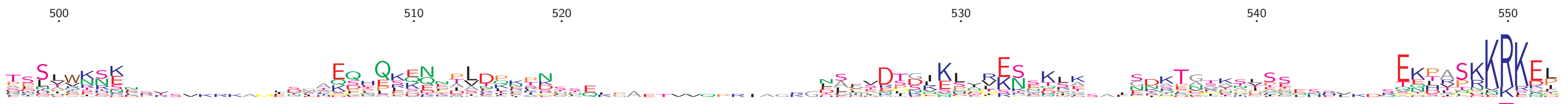

```
HsNBS1/NBN1                                                      TPSLWKNK.............EQHLSENEPVDTNSDN.................NLFTDTDLKSIVKNSASK..........SHAA.......EKLRSNKKREM   553
NP 038780.3 nibrin Mus musculus                                  GPSLWKSK.............E.HQSQNATLDREADT.................SSVGGMDIELNRKSPDRK..........PLPT.......ETLRPRKRKDV   552
XP 020944795.1 nibrin isoform X1 Sus scrofa                      TPSIWNNK.............EQHLSQNKPVEKKTD..................NLFADIDLKSTVKNSTRA.......MGVAVST.......EKLRSEKRKEI   551
XP 010586690.1 nibrin isoform X1 Loxodonta africana              TLSTWRHE.............EQNPFQNEPVDKKPD..................HVFTDTDLKSNVKNPTNA..........SLPP.......EKLTSKKRKEI   553
XP 028918726.1 nibrin Ornithorhynchus anatinus                   PASPCSSR.............ESQQLQKGPLTLEPKSS................DSFSRSNVEHDTEK.........RTAAGSLPE.......AESALKKRKKP   549
NP 989668.1 nibrin Gallus gallus                                 ASSAWNSE............KEQHGKGNN..............................IQLGRESGELA...SDKTDIKITFS.......ENPAPKKRKEL   544
XP 029866986.1 nibrin isoform X1 Aquila chrysaetos chrysaetos   ASSVWKSE............AQQHQKENNILAPNPN..................FALEDTGIKLMRESGKLA...SDKTDTKATSS.......EKHVPKKRKEL   559
XP 030122256.2 nibrin isoform X1 Taeniopygia guttata            TSLIRNTE............AKQHQKKNNILDPNAS..................FA.EDMSIKPMRESVKVT...SDRTNTETTSS.......ENHAPKRRKEL   557
XP 033921833.1 nibrin isoform X4 Melopsittacus undulatus        ASSVQNSE............AESYQKENNIPDLSPN..................FALDDTGIKLVRESAKLT...CDKTNTKTASS.......EKHASKKRKEL   559
XP 006266756.2 PREDICTED: nibrin isoform X1 Alligator mississippiensis  TSSTWKSK............IEQPQREQYTLDPKTN..................LLHIDTGIKCATESRKSE...SDKTAAKNISS.......TKPVSSKRKEQ   563
XP 008167725.1 nibrin Chrysemys picta bellii                     TSLMWKSK............VEQTQKEQFTLDLKTN..................LSPVDTSLKLAIESSKSE...KDKTVTKNVSS.......EKPASKKRKEL   594
XP 007441341.2 nibrin Python bivittatus                         PFLLWENS.............MDSQKVQCASKQGLN..................DSYMDSGKSLIMENNKLK.....QTGMESTSV.......EKPA..KRKEL   559
XP 013917592.1 PREDICTED: nibrin Thamnophis sirtalis             PFLLRENN.............VDSQKGQRAATQGVS..................DSNLDSRKSSIVENNKLK.....QTGMKSILL.......EMPASKKRKKI   554
XP 002934471.3 nibrin isoform X2 Xenopus tropicalis             TEALDKEED.............................................NFDSDLDMELAMSCDQIL......MGSNKVIP.......EDPTMKKRKVP   548
XP 018431775.1 PREDICTED: nibrin Nanorana parkeri                FSLPVTDS.............EMLSLDDVDLDKELN.....................VVPKEKPDLSNTRPK...GNNEASTSIV.......EKDASMKRKQP   557
XP 006008581.1 PREDICTED: nibrin isoform X1 Latimeria chalumnae  TTTLQKNS............TEKLQRKQCPSLIPSE..................PFVVEPKQKPSVRGENSGSAFNKSPEAVCVSS.......EKAASKKRKEP   567
NP 001014819.1 nibrin Danio rerio                                NRSHKTSSE....................................................................ETSLGQACGTG.......QNSSSKKRKEP   537
XP 030226503.1 nibrin Gadus morhua                               SSSAASRNP..........TGAQSPPGSEFDLFTRPMDTQ..............................................SRAGGVSHPSQ.......QGTLGRKRKEL   566
XP 007901387.1 PREDICTED: nibrin Callorhinchus milii             EPSVGRRDL..........DLTQFTQAERFSASFIIGSS...............TEGSKGNCEVVCKRETTG..INVPDKSIRLAD.......EVHRSNKRKGL   562
XP 032813356.1 nibrin isoform X2 Petromyzon marinus             PDNGTRGE...........DSHQPAPPKTTAGTPRTPAGELGA..........RG....................GNAEGVPAPSEQSH....TQTTTKKKKGF   596
XP 030831482.1 nibrin isoform X2 Strongylocentrotus purpuratus  RIETLGNKE..........AGSKPKQRQQGTLDAFRKPLE..............................KSEKPD...GKMKGRAGMEEESR...RVEDVNRVKEEP   587
NP 001246702.1 nbs, isoform D Drosophila melanogaster           DKHAVPAKRPV.......LSVASSDEEDEGDLFQFRKSPQKPAETVVQPRIAGKGNAPARISVVDFLEKSQAQ...EPAPVPPQLES.....QSQTQPRKRLRL   619
XP 026301749.1 nibrin isoform X1 Apis mellifera                SENLEIKKN.........ENIQKFEESKDSVVNKKIKLNE..................VNSEKENFLRNSKLK...EIKSNINNCQN......STQTLTDNKSDD   597
XP 005099806.1 PREDICTED: nibrin-like Aplysia californica       DDDVVNRGNRRSVKRKAMFSYDDEEEEKEENARPVKR.......................LGKKFVFETEEDS...NSTTAPAGDTELHEVKDSEEDISRERRDT   614
NP 010657.3 Xrs2p Saccharomyces cerevisiae S288C               KNREVKRVKST.......IVELKDEELSEEAINQLKN.................LAIVEPSNNLLRKSFDSE...GNKTSRTTEKWENSL...MEPEWHKRKNF   640
```

logo

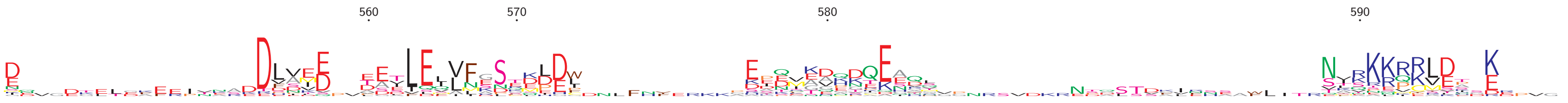

```
HsNBS1/NBN1                                                   D................DVAIE..DEVLEQLFKDTKPEL...........EIDVKVQKQEEDV..........................NVRKRPRMDI.E....  598
NP 038780.3 nibrin Mus musculus                               .................DLATE..EEVLEELLRSTKPEL...........AVQVKVEKQEADD..........................TIRKKPRMDA.E....  596
XP 020944795.1 nibrin isoform X1 Sus scrofa                   D................DLAIE..DEIFEQLFKNTKPEL...........KIEVEVQKQEEDV..........................NIRKRPRLDR.E....  596
XP 010586690.1 nibrin isoform X1 Loxodonta africana           D................DLAIE..DEVLEQLLKNTKPEL...........EIEMKVKKQEEDT..........................NIRKKPKLLI.E....  598
XP 028918726.1 nibrin Ornithorhynchus anatinus               D................DLALE..PDILQ.AIESFEHDC...........DSEMGEENQENGA..........................SVKKKQRLDA.K....  593
NP 989668.1 nibrin Gallus gallus                              D................DVSED..VETLEMVFESRDLDW...........EEQTANGDQEAQS..........................NKRKKRCLET.K....  589
XP 029866986.1 nibrin isoform X1 Aquila chrysaetos chrysaetos N................DLPED..TATLEIVFGSRDLDW...........EEQMADHDQEAQG..........................NTQKKICLEA.K....  604
XP 030122256.2 nibrin isoform X1 Taeniopygia guttata         D................DLSKD..TEALEIVFGSGDIDW...........EDQMTDHDQEAQS..........................SKRKKQCLEA.K....  602
XP 033921833.1 nibrin isoform X4 Melopsittacus undulatus     D................DLSED..TETLEIVFGSGDLDW...........EGQMADHDQEAQ.............................RKKRCLET.K....  601
XP 006266756.2 PREDICTED: nibrin isoform X1 Alligator mississippiensis D................DLIEE..TDTLEVVFGSKQLDW...........EDNVEDPGIEHQA..........................NTQKKRRLET.D....  608
XP 008167725.1 nibrin Chrysemys picta bellii                 G................DLVED..AATLELVFGSKELDW...........EAEMGDCGEEHGT..........................NMQKKRRLET.K....  639
XP 007441341.2 nibrin Python bivittatus                      E................DSVED..EASLELVFASQELDP...........EEDIGGQDEENAQ..........................NAKKKMKLDN.K....  604
XP 013917592.1 PREDICTED: nibrin Thamnophis sirtalis         E................DPVED..EASLELVFASQELDL...........EENIGEQDAKNTQ..........................NAKKKVKLDL.K....  599
XP 002934471.3 nibrin isoform X2 Xenopus tropicalis          D................DHVVE..ESDID.....SDEGI...........SANAEQNDTESNL..........................NKTKKRKLDS.K....  588
XP 018431775.1 PREDICTED: nibrin Nanorana parkeri           DN..............EDDVVE..ESDLE......NDEI...........SDQVKQKVPKSHS..........................VLVKRQRLDS.E....  598
XP 006008581.1 PREDICTED: nibrin isoform X1 Latimeria chalumnae N................DLDDD....LLLLAFESELLEG...........DADLTDQGTASVM..........................NVSRKRRMVP.E....  610
NP 001014819.1 nibrin Danio rerio                           EQ..DTLLGAEEPTAADDLEMS..LEELEFLMSDEMD.............EPPQTAANKKQRL.........ESGLTSKINSE......QLSNQQEVTESK....  606
XP 030226503.1 nibrin Gadus morhua                           EEVGDKELEKE...KADDLDME....ELESLMSLEMEDF..........GEPSRAAPDQQAQLVGNRSVDKRNPASTVAVEHS......STSKRPRVDP.RDPVG  647
XP 007901387.1 PREDICTED: nibrin Callorhinchus milii        D................DFNIQPVDGILE.......................DDAGPSREEWL..........................VISKRRKVEP.E....  597
XP 032813356.1 nibrin isoform X2 Petromyzon marinus          E................EFAME............................EPTEEGETQEGAGGP........................SAKRRRYADS......  627
XP 030831482.1 nibrin isoform X2 Strongylocentrotus purpuratus MD..EGETDFERIVERDDDDEE..AEEVE.......DDP...FHYERKKPKKTPEAGRTEERM..........................ESKAEQKVDGGKR...  649
NP 001246702.1 nbs, isoform D Drosophila melanogaster        E............................LLNESDSDDCDNLFNF....ADSKKKRKTQEAQR.........NDDSTDGLF........NFNSERPSDHDDE...  672
XP 026301749.1 nibrin isoform X1 Apis mellifera              I.........FEINNSKDENME.....IQIINSPCKDHF...........HTDINDKNCKIVQ..........................TLEENRKRSK.K....  646
XP 005099806.1 PREDICTED: nibrin-like Aplysia californica   S................RVVVD......SVVNDSEMDDI...........KPEVSAAPGD........................WL...SVATSVRVKP.E....  654
NP 010657.3 Xrs2p Saccharomyces cerevisiae S288C             K................TFVKV............................RPKSKAHKEEGKN.........NTQSSDFIRNAAFLITRNYVPLKKYSK.K....  687
```

logo

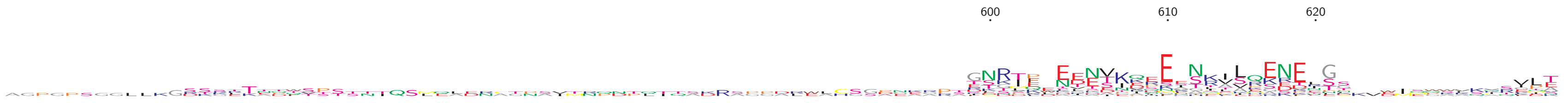

```
HsNBS1/NBN1                                                   .................................................................TNDTF.SDEA.VPESSKISQENEIG...............  621
NP 038780.3 nibrin Mus musculus                               .................................................................RNRPL.NGGS.EPESNSALQEDERE...............  619
XP 020944795.1 nibrin isoform X1 Sus scrofa                   .................................................................TNGTF.NDEA.IPERNKISQENEIG...............  619
XP 010586690.1 nibrin isoform X1 Loxodonta africana           .................................................................SNGMF.NDET.VSESNKVSQENEIG...............  621
XP 028918726.1 nibrin Ornithorhynchus anatinus               .................................................................ENNIP.EDKEIIQKNNRASEENELG...............  617
NP 989668.1 nibrin Gallus gallus                              .................................................................GSRTE.EGNTKQREENEMLRKEEVGS...........VLT  617
XP 029866986.1 nibrin isoform X1 Aquila chrysaetos chrysaetos .................................................................GSRIQ.EANVKQREENKI......................  621
XP 030122256.2 nibrin isoform X1 Taeniopygia guttata         .................................................................GSRIP.EVNVNQREENKVLKEEELGS...........VLT  630
XP 033921833.1 nibrin isoform X4 Melopsittacus undulatus     .................................................................RSRIQ.EVNVKQREENKILKEEVLGS...........VLT  629
XP 006266756.2 PREDICTED: nibrin isoform X1 Alligator mississippiensis .................................................................ENRIE.KENVTRNEGSRIL.ENEFGC...........VLT  635
XP 008167725.1 nibrin Chrysemys picta bellii                 .................................................................GKRIE.EENVIQEEANRILQENELGT...........VPT  667
XP 007441341.2 nibrin Python bivittatus                      .................................................................G.....EIST.KVEATRLLEKNEES............IPA  626
XP 013917592.1 PREDICTED: nibrin Thamnophis sirtalis         .................................................................ETTLR.NTET.KIEATRFLGKNEES............LPA  625
XP 002934471.3 nibrin isoform X2 Xenopus tropicalis          .................................................................IEGVK.ELTIKPHEIGTKAQAKTNTP..EMEPSVKK.EIE  624
XP 018431775.1 PREDICTED: nibrin Nanorana parkeri           .................................................................GDSGE......DLKPGLPARGNRTTL..WIQQAAQQSEQK  630
XP 006008581.1 PREDICTED: nibrin isoform X1 Latimeria chalumnae .................................................................GKKMKLEETTPAEEISTAKQSSENST.........IRKEE  641
NP 001014819.1 nibrin Danio rerio                           ...........GRKGEKNQQSSSSNIQSMQLDRAGPAVTNQDTQTQSKRSPPDLEAHSSANKGPTKNKTPELEEVKKEEVSFVVNSRPQNG..............  686
XP 030226503.1 nibrin Gadus morhua                           AGPGPSGGLLKGSTSTTTTTTTTTTQSLEAENCTNSIMRPNPLITADRAGGARWLCGGPEEAPAGTRSQEALAVKDENVSMVVDGSEALG...........GTA  741
XP 007901387.1 PREDICTED: nibrin Callorhinchus milii        .................................................................DLSLP.EKPSKAEFDHKLLHSDKRTS.............E  623
XP 032813356.1 nibrin isoform X2 Petromyzon marinus          .........................................................................................................  627
XP 030831482.1 nibrin isoform X2 Strongylocentrotus purpuratus ............SSKRTADWS..................................................DENDGESSRAQTSRENDGGG....MWKKKARIDV  688
NP 001246702.1 nbs, isoform D Drosophila melanogaster        ............DSRLTEPFVP.................................................ETESKKQSKYIVAPRRDRPKKVDISGWLSCSRLN  716
XP 026301749.1 nibrin isoform X1 Apis mellifera              .................................................................TCKTF.NENDIDEEFTISIRKK..................  667
XP 005099806.1 PREDICTED: nibrin-like Aplysia californica   .....................PS.................................CSGDGARRSFNRDVDTEDSERDGISGAVPADTHSD.........VQGLE  696
NP 010657.3 Xrs2p Saccharomyces cerevisiae S288C             .................................................................DTTTKWGTEENEDMFALTEMERFGSN..........TFMS  717
```

logo

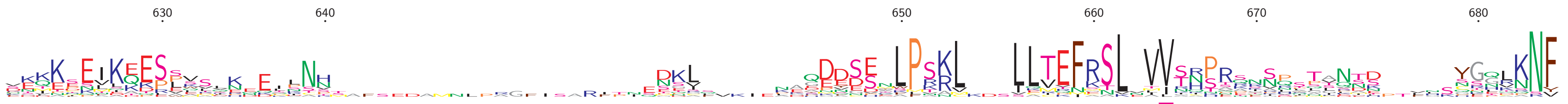

```
HsNBS1/NBN1                                                   ..KKRELKEDSLWSAK.EISNN......................DKL.......QDDSEMLPKKL...LLTEFRSL.VIKNSTSRNPSGINDD.....YGQLKNF  685
NP 038780.3 nibrin Mus musculus                               ..KKDELQTESWSTKH.EIANS......................DGL.......QDSSEELPRKL...LLTEFRSL.VVSNHNSTSRNLCVNE.....CGPLKNF  683
XP 020944795.1 nibrin isoform X1 Sus scrofa                   ..KKCELKKESLWSVK.EIPNN......................SEL.......EDEGEMLPRKV...LLTKFESL.VVSNSFSRNLSSINND.....YGELKNF  683
XP 010586690.1 nibrin isoform X1 Loxodonta africana           ..KKCENKEESLLSSKEELSNN......................DEL.......QDDGEKLPRKV...ILTEFRSL.VVTNSASRNPSLVDND.....YGQLKNF  686
XP 028918726.1 nibrin Ornithorhynchus anatinus               ..KTDEIKKKSPERVPEQQNNN......................ENR.......QDDSEVLPKKL...LLTEFRSL.VVGHTRARNSSTVRSD.....YGHRNNF  682
NP 989668.1 nibrin Gallus gallus                              LEDKSKIKEESSVSIRNKLINH......................NKL.......EDDSSRLPSKL...LLTEFRSL.VVSCPRSNSPTMRNTK.....CRGQNNF  684
XP 029866986.1 nibrin isoform X1 Aquila chrysaetos chrysaetos ...................LNH......................NKL.......QDDSSNLPSRL...LLTEFRSL.VVSHPRQNSQLTGNTS.....YGGKKNF  669
XP 030122256.2 nibrin isoform X1 Taeniopygia guttata         SEMKSNIKQESPV......LNH......................SKL.......QDDSSNLPSRL...LLTEFRSL.VVSHPRQKSHFPGNTS.....YGGKKNF  691
XP 033921833.1 nibrin isoform X4 Melopsittacus undulatus     PEMKSEFKEQSPV......LNH......................NKV.......QDDSSNLPSRL...LLTEFRSL.VVTRPRQNSQLTGNTS.....CGGKKNF  690
XP 006266756.2 PREDICTED: nibrin isoform X1 Alligator mississippiensis SEKENKIKQESSM......LSH......................DKL.......HDDSHSLPSKL...LLTEFRSL.VVSHPRPNAQITAKNN.....YGELKNY  696
XP 008167725.1 nibrin Chrysemys picta bellii                 SKKKIEIKQESSV......LNT......................DGL.......QDVSSNLPSRL...LLTEFRSL.VVSHPRPNSLVAAQIN.....YKQLNNF  728
XP 007441341.2 nibrin Python bivittatus                      KKQKEEMKEESPK......PNP.................................DDSSYLPSKL...LLMEFRSL.VVTQPRKIG.CPANSD.....YSHLNNF  682
XP 013917592.1 PREDICTED: nibrin Thamnophis sirtalis         ETQKEEIKEEPPE......PNP......................GKC.......GDVSSYLPNKL...LLMEFRSL.VVTQPRKIS.PQANSD.....YGHLSNF  685
XP 002934471.3 nibrin isoform X2 Xenopus tropicalis          VKTEFEVKKEPESPQK.ETKCV......................PDL.......AGESDALPSRL...LLTEFKSL.VVTRPVRNNNTTSNSS.....NENCQNF  690
XP 018431775.1 PREDICTED: nibrin Nanorana parkeri           VKTEPSIKQEPESLYE.DKKTVM.....................MTVQ....PKGENDDNFPSRL...LLTEFRTLVVVSRPATYSQYTANAK.....QGNGPNF  701
XP 006008581.1 PREDICTED: nibrin isoform X1 Latimeria chalumnae NELAESVKKERAA...............................SKAL....LAGEYDENLPTKL...LLIEFKSL.VVSRPRAFVQSTTSME.....QGQVKNY  702
NP 001014819.1 nibrin Danio rerio                           ......ISQTSEAVLKQEMRAST....................SNSG.......PKNDPDLPRKL...LQVQFMSL.TVNNSSRSRPGPLQTH.....NPNDKNV  749
XP 030226503.1 nibrin Gadus morhua                           RGQPMEVLEKPLKVFK...........................QDKI......ASEAEEGLPKRL...VLVEFKSL.MVTAPPRGKPQQTQG......PSSTKNF  803
XP 007901387.1 PREDICTED: nibrin Callorhinchus milii        VKQKTEILDPLAVSLN.ESNNH......................EKLF....NEAEYNEMLPSKL...LLTVFKTL..VNHTRQKIVPTSNAD.....NSQVKNF  691
XP 032813356.1 nibrin isoform X2 Petromyzon marinus          ....PPVKQEPVV.......................................AGDPEDVTAPTAL...LLCEFRSL.VLARPTASSHAGLPDTPTLLSGTLRRNF  685
XP 030831482.1 nibrin isoform X2 Strongylocentrotus purpuratus FSQEEEAAAESSQSQEDEPDSRSAFSEDAMNLPRGFISARIPIEDQVSVKIEKDYMQEEGLPSKL...IKVTNAAL.VIRKKRNPPRRAVQEL...GSGRQVKNF  786
NP 001246702.1 nbs, isoform D Drosophila melanogaster        DNIKSEIDADSVKM.............................ETSI.....KADPDEEQWLAAMKDSIEVRMCNLNIVIRSQEEVDASLEDS..VNKHGGRKNF  785
XP 026301749.1 nibrin isoform X1 Apis mellifera              ....................................................................SCVENSNVELITINEEQNNSNTLTK..FHSSFLRNRF  702
XP 005099806.1 PREDICTED: nibrin-like Aplysia californica   DDVKPQMEEEELLLDN.................AG.........NPL.......RNFCEVKMVNL...LARPIGSK.AVRNSSKGAPSTS........SNGLKNF  756
NP 010657.3 Xrs2p Saccharomyces cerevisiae S288C             DNINSNTIQKRSQALN.......................RFTNEDSS.....NEGEEDSFSFSRC.................................SGTAASV  761
```

logo

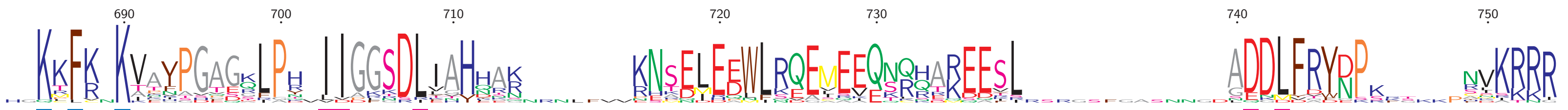

```
HsNBS1/NBN1                                                   ..KKFK.KVTYPGAGKLPH.IIGGSDLIAHHAR.......KNTELEEWLRQEMEVQNQHAKEESL.............ADDLFRYNP......YLKRRR  754
NP 038780.3 nibrin Mus musculus                               ..KKFK.KATFPGAGKLPH.IIGGSDLVGHHAR.......KNTELEEWLKQEMEVQKQQAKEESL.............ADDLFRYNP......NVKRR.  751
XP 020944795.1 nibrin isoform X1 Sus scrofa                   ..KKFK.KVAYPGAGKLPH.IIGGSDLIAYHAR.......KNTELEDWLKQEMEVQNQHAKEESL.............ADDLFRYNP......NVKRRR  752
XP 010586690.1 nibrin isoform X1 Loxodonta africana           ..KKFK.KVTFPGAGKLPH.IIGGSDLIAHHAQ.......KNRELEEWLRQEMEVQNQHAKEESL.............ADDLFRYNP......NVKRRR  755
XP 028918726.1 nibrin Ornithorhynchus anatinus               ..KKFK.KVAYPGAGKLPQ.IIGGSDLIAHHAR.......KNAELEEWLRQEMEIQSQRAKEESL.............ADDLFRYNP......NGKKRR  751
NP 989668.1 nibrin Gallus gallus                              ..KTFR.KVPYPGAGQLPY.IIGGSDLVAHQAR.......KNSELEEWLREELEEQNRRAREESL.............ADDLFRYDP......NVKRRR  753
XP 029866986.1 nibrin isoform X1 Aquila chrysaetos chrysaetos ..KMFK.KVAYPGAGQLPY.IIGGSDLIAHHAK.......KNSELEEWLRQEMEEQNRHAREESL.............ADDLFRYDP......NVKRRR  738
XP 030122256.2 nibrin isoform X1 Taeniopygia guttata         ..KMFK.KVAYPGAGQLPH.IIGGSDLIAHHAK.......KNSELEDWLRQEIEEQNRHAREESL.............ADDLFRYDP......NVKRR.  759
XP 033921833.1 nibrin isoform X4 Melopsittacus undulatus     ..KLFK.KVAYPGAGQLPY.IIGRSDLIAHQAK.......KNSELEEWLREEMEEQNRHAREESL.............ADDLFRYDP......NVKRRR  759
XP 006266756.2 PREDICTED: nibrin isoform X1 Alligator mississippiensis ..KKFK.KVAYPGVGQLPH.IIGGSDLIAHNAK.......RNSELEEWLRQEMEEQNRHEKEESL.............ADDLFRYDP......HVKRRR  765
XP 008167725.1 nibrin Chrysemys picta bellii                 ..KKFK.KVPYPGAGQLPH.IIGGSDLIGHHAK.......KNSQLEEWLRQEMEEQNRHAREESL.............ADDLFRYDP......NVKRRR  797
XP 007441341.2 nibrin Python bivittatus                      ..KKFK.KVAYPGAGRLPN.IIGGSDLIAHHAR.......RNSEMEDWLRQEMEEQSRQTREESL.............GDDLFRYDP......RIKRR.  750
XP 013917592.1 PREDICTED: nibrin Thamnophis sirtalis         ..KKFK.KIAYPGAGRLPN.IIGGSDLIAHHAR.......RNPDMEDWLRQEMEEQNCQTREESL.............ADDLFRYDP......RIKRR.  753
XP 002934471.3 nibrin isoform X2 Xenopus tropicalis          ..KKFK.KVAYPGAGSFPH.IIGGSDLIAHDRK.......RNAELEQWLRQEVEEQTQQAKEESL.............AEDLFRYNPK.....SNKRRR  760
XP 018431775.1 PREDICTED: nibrin Nanorana parkeri           ..KKFR.KTAFPGAGSFPH.IIGGSDLIAHDKK.......KNSEIEQWLRQEMEEQTQQAREQSL.............AEDLFRYNPK.....NMKKRR  771
XP 006008581.1 PREDICTED: nibrin isoform X1 Latimeria chalumnae ..KRFK.KVAYPGAEGLPH.IIGGSDLIAHQSK.......KNSELEEWLQQEMQEQSQRAREEFL.............SDDLFRYDPK.....SVKRRR  772
NP 001014819.1 nibrin Danio rerio                           ..KRFR.KKNVPGFDGLPK.IIGGSDLVAHNRS.......KHSELEEWLRQAAEEEKLNEREEYL.............GDDLFRYNP......RPAKKR  818
XP 030226503.1 nibrin Gadus morhua                           ..KCFC.KVRGSSTERLAR.LIGGGDLLAHNRG.......KNSELDDLLKDASEEEQQSRRDESL.............GDDLFRYNPS.....RLSKKR  873
XP 007901387.1 PREDICTED: nibrin Callorhinchus milii        ..KKFR.KVLYPGGEGLPR.IIGGTDLKAYQTK.......NNSEMEEWLRQEIEEQSQQAKDDIL.............ADDLFRYDPK.....SVKRRR  761
XP 032813356.1 nibrin isoform X2 Petromyzon marinus          ..KTFR.KAARGVTSTLPRVVIAFSDLYHHE.........EESDDHAWI.....EESQRTQEEVL.............ADNLFG...............  739
XP 030831482.1 nibrin isoform X2 Strongylocentrotus purpuratus ..KKFR.KAGYAGQSAIPR.IIGGNDLFTHYNN.......PSKDIEDMFNEEVRAEKKKDKQEVI.............AEKLFAFEPRRG..PASKRKR  859
NP 001246702.1 nbs, isoform D Drosophila melanogaster        ..KKFV.KTKNPHPQKR...IVALKSL....................................RL.............ADGMVT.........CV....  816
XP 026301749.1 nibrin isoform X1 Apis mellifera              HGKTFK.KVYN.........LIPKKRIT..................................................LDDMYVWNK......CDITNI  735
XP 005099806.1 PREDICTED: nibrin-like Aplysia californica   ..KKFR.KTEHAGTGKLPT.IIGGRDLEVYLGN.......RHKDIEDLFSVGLAEENRQRMEDKR.............NQDLFDWDSSKSKKPAVKKRR  831
NP 010657.3 Xrs2p Saccharomyces cerevisiae S288C             ..QPLKNKIFITDEDDLGD.IDDKSDRLNHRENNRNLFVVKEMNLRPNLSEECSKQSRHSRSATSRSRGSFGASNNGDGDDDDDDGPKFT...FKRRKG  854
```

X non-conserved
X similar
X ≥98% conserved

Supplementary Figure S2. Tissue distribution of MRN mutations of unknon origin.

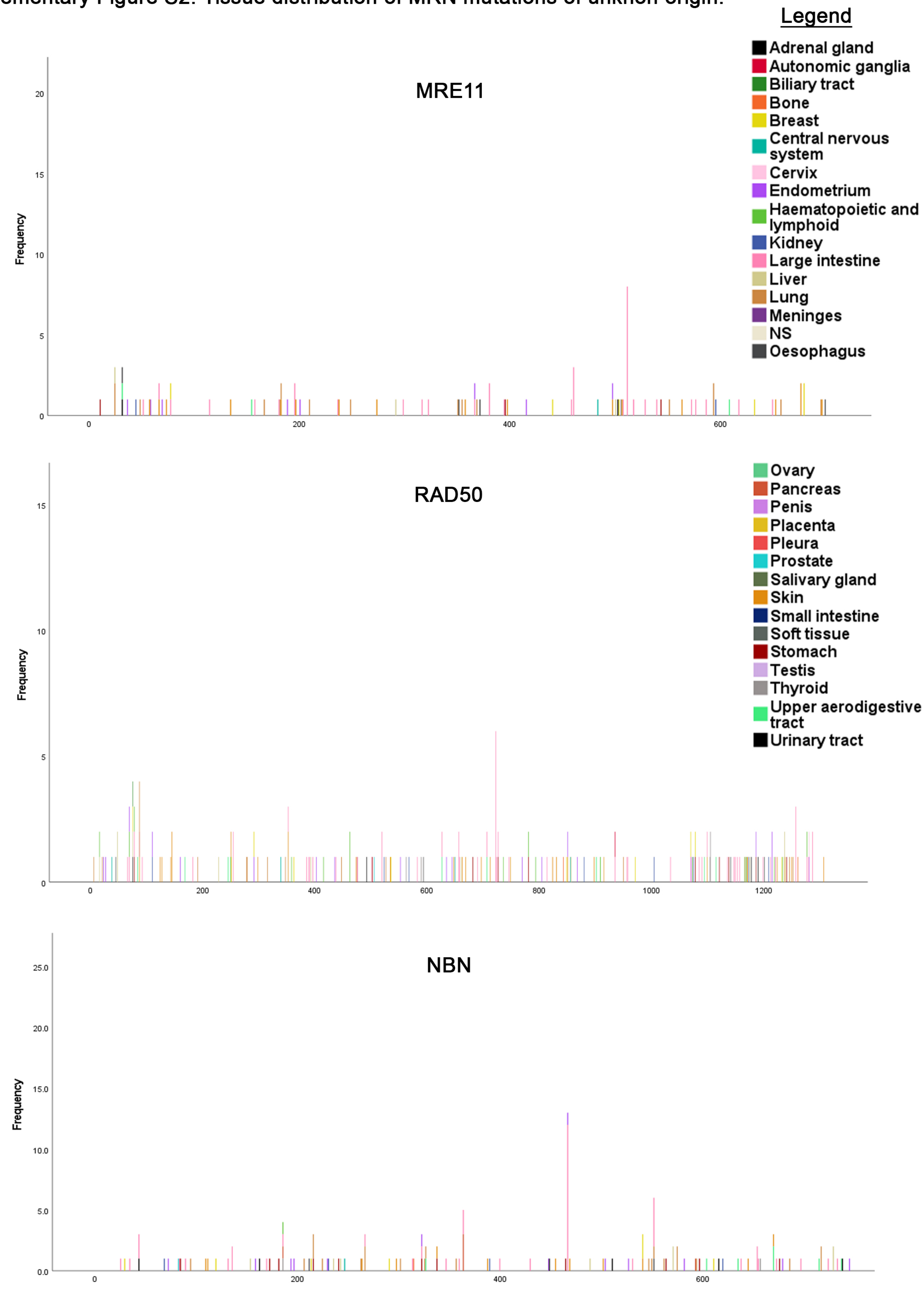

Supplement: Supplementary file 1 [file cancers-12-03794-s001.pdf]
